# A halogen bond-mediated highly active artificial chloride channel with high anticancer activity†
†Electronic supplementary information (ESI) available. See DOI: 10.1039/c8sc00602d


**DOI:** 10.1039/c8sc00602d

**Published:** 2018-03-15

**Authors:** Changliang Ren, Xin Ding, Arundhati Roy, Jie Shen, Shaoyuan Zhou, Feng Chen, Sam Fong Yau Li, Haisheng Ren, Yi Yan Yang, Huaqiang Zeng

**Affiliations:** a Institute of Bioengineering and Nanotechnology , 31 Biopolis Way, The Nanos , Singapore 138669 . Email: hqzeng@ibn.a-star.edu.sg; b College of Chemical Engineering , Sichuan University , Chengdu , China 610065; c NUS Environmental Research Institute , Department of Chemistry , National University of Singapore , 3 Science Drive 3 , Singapore 117543

## Abstract

Chloride-selective transmembrane carriers or channels might have possible uses in treating channelopathies or cancers. While chloride carriers have been extensively investigated, the corresponding chloride channels have remained limitedly studied. Moreover, all hitherto reported channel systems lack clearly definable and readily modifiable positions in their structures for the reliable construction and combinatorial optimization of their ion transport properties. As a result, the existing channels are limited by their large molecular weight, weak activity or low anion selectivity. In this report, we describe a readily accessible and robust monopeptide-based scaffold for the reliable construction of halogen bond-mediated artificial anion channels *via* directional assembly of electron-deficient iodine atoms, which create a transmembrane pathway for facilitating anion transport. The high intrinsic modularity of the backbone of the scaffold, which enables the rapid and combinatorial optimization of the transport activity and selectivity of channels, effectively delivers a highly active chloride channel **A10**. Such high activity in chloride transport subsequently leads to an excellent IC_50_ value of 20 μM toward inhibiting the growth of human breast cancer cells (BT-474), an anticancer activity that is even higher than that of the well-known anticancer agent cisplatin.

## Abstract 2

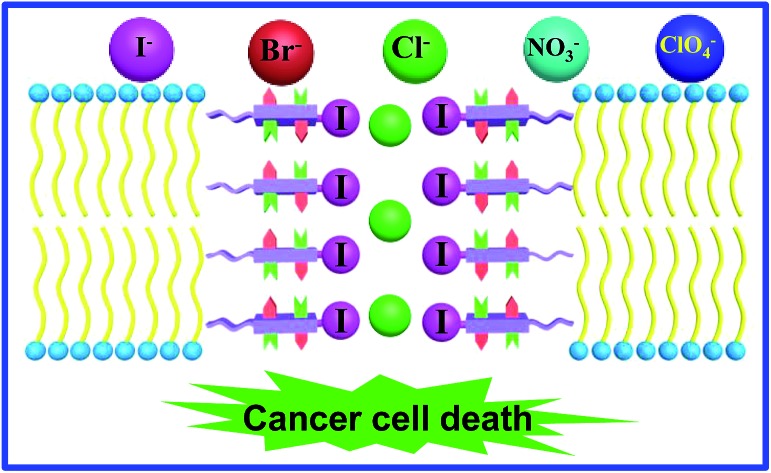
Modularly tunable monopeptidic scaffold enables rapid and combinatorial evolution of a halogen bond-mediated highly active chloride channel, exhibiting an excellent anticancer activity toward human breast cancer.

## Introduction

Tightly regulated by varying families of ion channels, precise maintenance of ion concentration gradients across biological membranes is crucial for many biological and cellular processes. Chloride is the most abundant anion in the human body due to its important roles in controlling the membrane potential, cell volume, cellular pH balance, secretion of transepithelial fluid and electrolytes, *etc.*^1^ Misregulation of chloride ions could lead to a variety of life-shortening ‘channelopathies’ including cystic fibrosis, Bartter syndrome and Dent's disease. Accordingly, significant research efforts have been made recently to coherently develop artificial chloride carriers^2^ or channels^3^ as possible replacements for natural chloride channels for channelopathies.^4^ On the other hand, artificial anion transporters might also disrupt pH gradients4f,5a,b or ion homeostasis5*c* that may induce cell death, and therefore may have medical applications as anticancer agents.4f,^5^

Recently, Matile *et al.* reported self-assembled chloride carriers2b,c or channels,3*i* elegantly exploiting halogen bonding interaction as the driving force for anion transport. Halogen bonds offer good bond strength and also good anion-induced directionality.^6^ Therefore, artificial anion transporters comprising simple structural motifs based on halogen–anion interactions may indeed offer a new horizon of prospects to comprehend complex chloride transport phenomena, often manifested in protein channels. Nevertheless, most documented artificial chloride channels purposefully harness various non-covalent interactions such as electrostatic, H-bonding, anion-π and halogen bonding forces. Channels that primarily rely on halogen bonds for facilitating anion transport still remain limitedly investigated with just one precedent.3*i* Such a rare occurrence presumably results from the lack of suitable molecular scaffolds for aligning electron-deficient iodine atoms into a well-defined one-dimensional (1D) array. Moreover, the majority of existing chloride channels3a–m suffer from relatively complex molecular structures and/or low selectivity in anion recognition, greatly restricting their application in drug discovery.

**Fig. 1:**
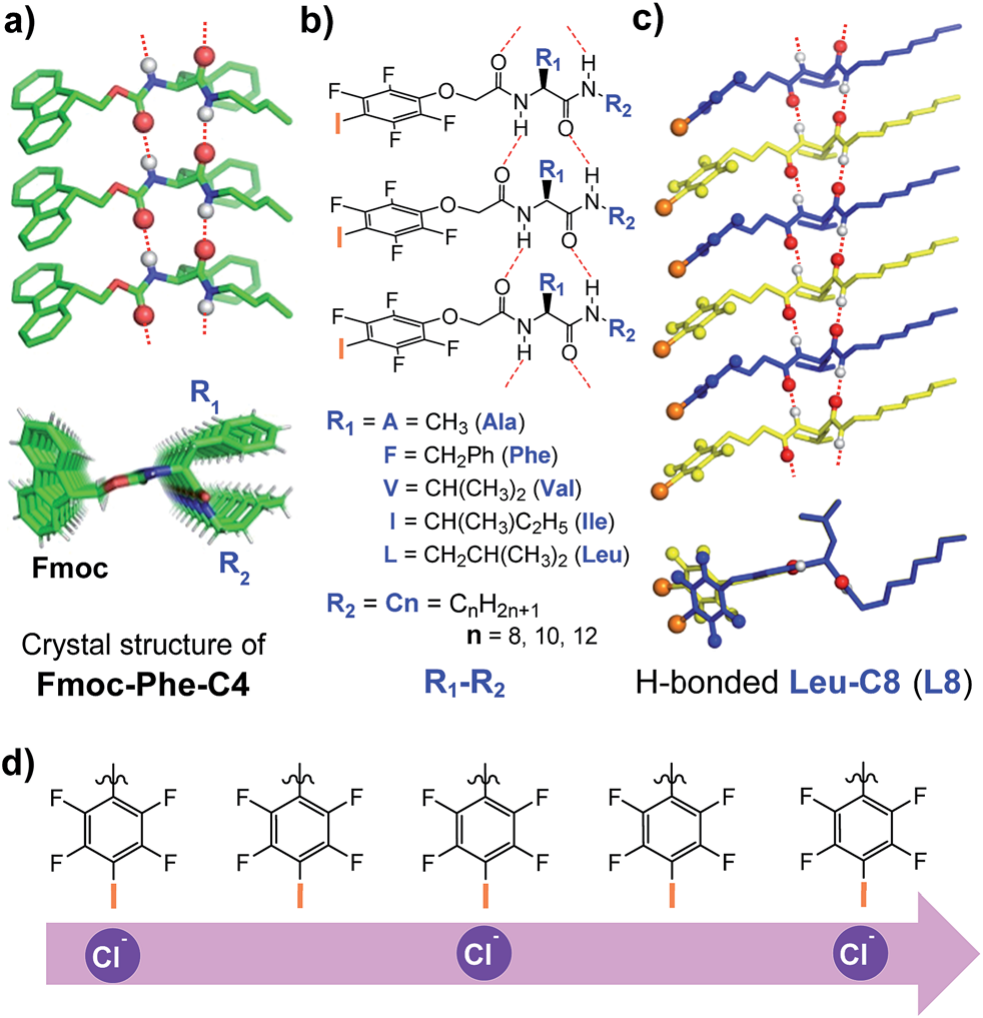
(a) Crystal structure of **F-Phe-C4**, illustrating the formation of the 1D columnar stack with directionally assembled side chains. (b) Molecular design of the chloride-transporting channel library with systematically tunable R_1_ and R_2_ groups. (c) Side and top views of the computationally optimized H-bonded 1D columnar ensemble formed from **L8**. (d) A possible multi-ion jumping mode responsible for chloride transport.

We recently described a novel class of readily accessible monopeptide-based scaffolds capable of self-assembling into 1D columnar structures through highly directional intermolecular H-bonding forces, subsequently enabling rapid room-temperature gelation of diverse types of crude oils (Fig. 1a).7a–c Revealed by the crystal structure of **F-Phe-C4** (Fig. 1a), one unique feature of this monopeptide-based scaffold is that the same type of side chains such as Fmoc, R_1_ and R_2_ are always aligned to the same side as a result of the high directionality of H bonds (Fig. 1a).7*a* On this basis, we envisioned that replacing the Fmoc group with a chloride-binding unit such as the tetrafluoroiodobenzyl group may lead to an interesting class of artificial anion channels with anion transport mediated by halogen bonds (Fig. 1b–d).3i,7d A high intrinsic modularity involving R_1_ and R_2_ groups in the backbone should allow for rapid and combinatorial optimization of transport activity and selectivity of channels. In this report, we demonstrate that this indeed is the case, with the identified chloride channel exhibiting a high activity in chloride transport with respect to many other types of anions. We further demonstrate that human breast cancer cells (BT-474) are sensitive to a sodium chloride concentration gradient across the membrane, and this feature can be utilized by artificial chloride channels to inhibit cancer cell growth for their potential uses in cancer chemotherapy.4f,^5^

## Results and discussion

### Combinatorial identification of highly active anion-transporting artificial channels

**Fig. 2:**
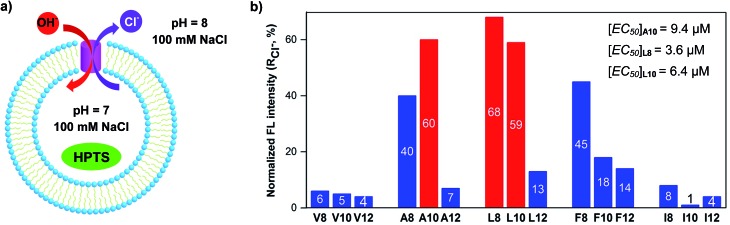
(a) Schematic illustration of the HPTS assay for the Cl^–^ transport study conducted using a pH gradient of 7 to 8 and the pH-sensitive dye HPTS entrapped inside LUVs. (b) Normalized transport activities (*R*_Cl^–^_) obtained over 5 min at 10 μM for all channel molecules. *R*_Cl^–^_ = (*I*_Cl^–^_ – *I*_0_)/(*I*_Triton_ – *I*_0_) wherein *I*_Cl^–^_ and *I*_0_ are the ratiometric values of *I*_460_/*I*_403_ before the addition of triton at *t* = 300 s, and *I*_Triton_ is the ratiometric value of *I*_460_/*I*_403_ at *t* = 300 s right after the addition of triton with internal/external buffers containing 100 mM NaCl. All the data were averaged over two measurements.

We synthesized a total of 15 possible channel molecules (five amino acids × three alkyl chains of different lengths, Fig. 1b) with the tetrafluoroiodobenzyl group as the chloride-binding and -transporting unit. Corroborated by the crystal structure of **F-Phe-C4** (Fig. 1a) and the computationally optimized structure of **L8** (Fig. 1c), the ability of these 15 molecules to pile up to form a H-bonded 1D structure can be convincingly demonstrated by the ability of **A10**, **L8** and **L10** to efficiently congeal in *n*-hexane *via* the formation of a 3D entangled fibrous network (ESI, Fig. S1†). The ion transport activities of the synthesized channels were then evaluated using the pH-sensitive HPTS (8-hydroxypyrene-1,3,6-trisulfonic acid) assay (Fig. 2 and S2†).^8^ In a typical experiment, large unilamellar vesicles (LUVs), encapsulating HPTS (100 μM) and NaCl (100 mM) at pH = 7.0, were diluted into the same buffer at pH 8.0 to generate a pH gradient across LUVs. After the addition of channel molecules, changes in the fluorescence intensity of HPTS were monitored over 5 min. Fig. 2b shows that, among all 15 channels tested at 10 μM, **A10**, **L8** and **L10** display the highest ion transport capacities with fractional activities (*R*_Cl^–^_) of 60%, 68% and 59%, respectively. The corresponding EC_50_ values, which are the concentrations required to reach 50% transmembrane activity, were determined *via* Hill analyses to be 9.4, 3.6 and 6.4 μM, respectively (Fig. S3†).

### Cl^–^/OH^–^ as the major transport species

**Fig. 3:**
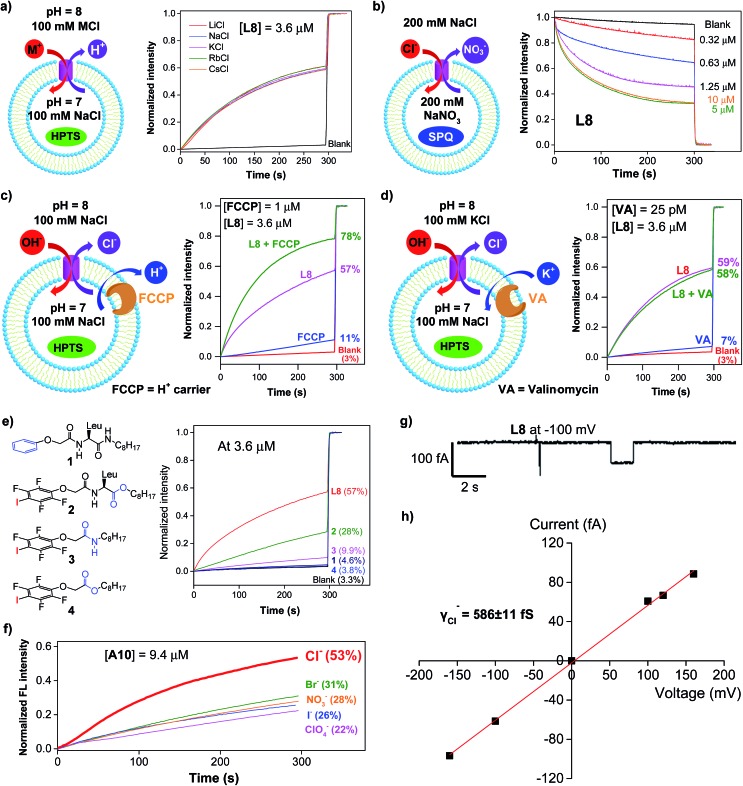
(a) Transport selectivity of alkali metal ions by **L8** (3.6 μM) obtained from the HPTS assay by varying the extravesicular MCl (M^+^ = Li^+^, Na^+^, K^+^, Rb^+^ and Cs^+^). (b) Concentration-dependent changes in the fluorescence intensity of the chloride-sensitive SPQ dye (*λ*_ex_ = 360 nm, *λ*_em_ = 430 nm) after the addition of **L8** at different concentrations. Inside LUV: 200 mM NaNO_3_ and 0.5 mM SPQ. Outside LUV: 200 mM NaCl. (c) Ion transport activities of **L8** (3.6 μM) determined in the absence or presence of proton transporter FCCP (1 μM), indicating a preferential transport of Cl^–^ over H^+^. (d) Ion transport activities of **L8** (3.6 μM) determined in the absence or presence of a potassium carrier, valinomycin (VA, 25 pM), indicating a preferential transport of Cl^–^ over OH^–^. (e) Ion transport activities of four control compounds **1–4** at 3.6 μM, suggesting a critical role of halogen bonds in mediating the transport of Cl^–^ and OH^–^. (f) Anion selectivity assayed for **A10** (For **L8** and **L10**, see Fig. S10†). (g) Single current trace of **L8** recorded at –100 mv with symmetric baths (*cis* chamber = *trans* chamber = 1 M KCl). (h) *I*–*V* curve for obtaining the Cl^–^ conductance (*γ*_Cl^–^_) of **L8**. All the data were averaged over two measurements.

Except for the presence of arrayed electron-deficient iodine atoms that can bind the Cl^–^ anion,2b,c,3i all peptide molecules carry no other readily accessible functional groups for strongly binding either cationic or anionic species. Further, varying extravesicular metal chloride salts from LiCl to CsCl produces near-identical changes in fluorescence intensity (Fig. 3a and S4†), suggesting the inability of **L8** or **A10** to transport any of the five alkali metal ions. These combined structural features and experimental evidence led us to believe that, rather than the H^+^/M^+^ antiport mechanism, either the OH^–^/Cl^–^ antiport (Fig. 2a) or H^+^/Cl^–^ symport mechanism may largely account for the observed increases in the fluorescence intensity of HPTS.

To deduce the most likely transport species, we first carried out the SPQ assay^9^ using a chloride-sensitive SPQ dye (6-methoxy-*N*-(3-sulfopropyl)quinolinium). As illustrated in Fig. 3b and S5,† the addition of **L8** or **A10** results in a rapid quenching of SPQ fluorescence in a concentration-dependent manner. These results suggest that the influx of Cl^–^ increases with increasing channel concentrations from 0.32 to 5 μM for **L8** and from 2.5 to 20 μM for **A10**, thereby establishing the Cl^–^ anion as one of the molecular species transported by both **L8** and **A10**.

Next, carbonyl cyanide 4-(trifluoromethoxy)phenylhydrazone (FCCP, a well-known proton carrier) was employed (Fig. 3c). Compared to the transport efficiencies of 11% for FCCP alone and of 57% for **L8** alone, an increased transport efficiency of 13% (*e.g.*, 78–57% – (11–3%)) for **L8** in the presence of FCCP indicates a cooperative action between **L8** and FCCP. In the case of **A10**, an even larger enhancement of 25% was observed (Fig. S6†). These results suggest that the transport rate of Cl^–^ is faster than that of H^+^.

The HPTS assay was then performed in the presence of valinomycin (VA, a K^+^-selective carrier, Fig. 3d) to compare the transport rate between OH^–^ and Cl^–^. For this assay carried out under iso-osmolar K^+^ (extravesicular) *vs.* Na^+^ (intravesicular) and pH gradient, the VA-mediated influx of K^+^ ions will induce an anion channel-mediated influx of either OH^–^ or Cl^–^ in order to maintain overall charge equality. If the influx of OH^–^ is faster than that of Cl^–^, an increase in fluorescence intensity is anticipated. Experimentally, valinomycin at 25 pM produces a small increment of fluorescence intensity (7%) compared to the blank (3%). In a similar way, the ion transport activities of **L8** (3.6 μM) in the presence and absence of valinomycin were found to be near-identical (58% *vs.* 59%), which is indicative of a preferential transport of Cl^–^ over OH^–^ (*e.g.*, Cl^–^ > OH^–^). A similar result was also seen for **A10** (Fig. S7†). Consistent with transport activities presented in Fig. 3a, these data also suggest that the H^+^/M^+^ antiport mechanism is unlikely for **L8**-mediated enhancement in fluorescence intensity.

A mere increase of 1.3% (4.6–3.3%, Fig. 3e) in fluorescence intensity at 3.6 μM for molecule **1**, carrying a simple benzene group, strongly suggests a lack of readily accessible functional groups in the H-bonded peptidic backbone for interacting with either anions or protons, and thus a minor role played by the backbone in mediating chloride transport. Instead, it is halogen bonds formed between anions (*e.g.*, Cl^–^ or OH^–^) and electron-deficient iodine atoms that cause efficient exchanges between Cl^–^ and OH^–^ anions across LUVs as observed for **L8** (57%), pointing to a high unlikelihood of having H^+^/Cl^–^ as the transport species. The herein assumed formation of halogen bonds I···Cl^–^ and I···OH^–^ was recently observed in their corresponding crystal structures2*c* and can be further supported by ^19^F NMR titration experiments involving titrating 0–20 equiv. of tetrabutylammonium chloride into a D_2_O-saturated CDCl_3_ solution containing **L8** at 1 mM (Fig. S8†). Relative to the internal standard (1,4-difluorobenzene, –120.50 ppm), increasing additions of up to 20 equiv. of TBACl led to increasing upfield shifts of up to 0.61 ppm in the chemical shift of ^19^F of **L8**, a fact that is consistent with earlier observations2*b* and indicates binding between the chloride anion and the acidic iodine atom.

**Fig. 4:**
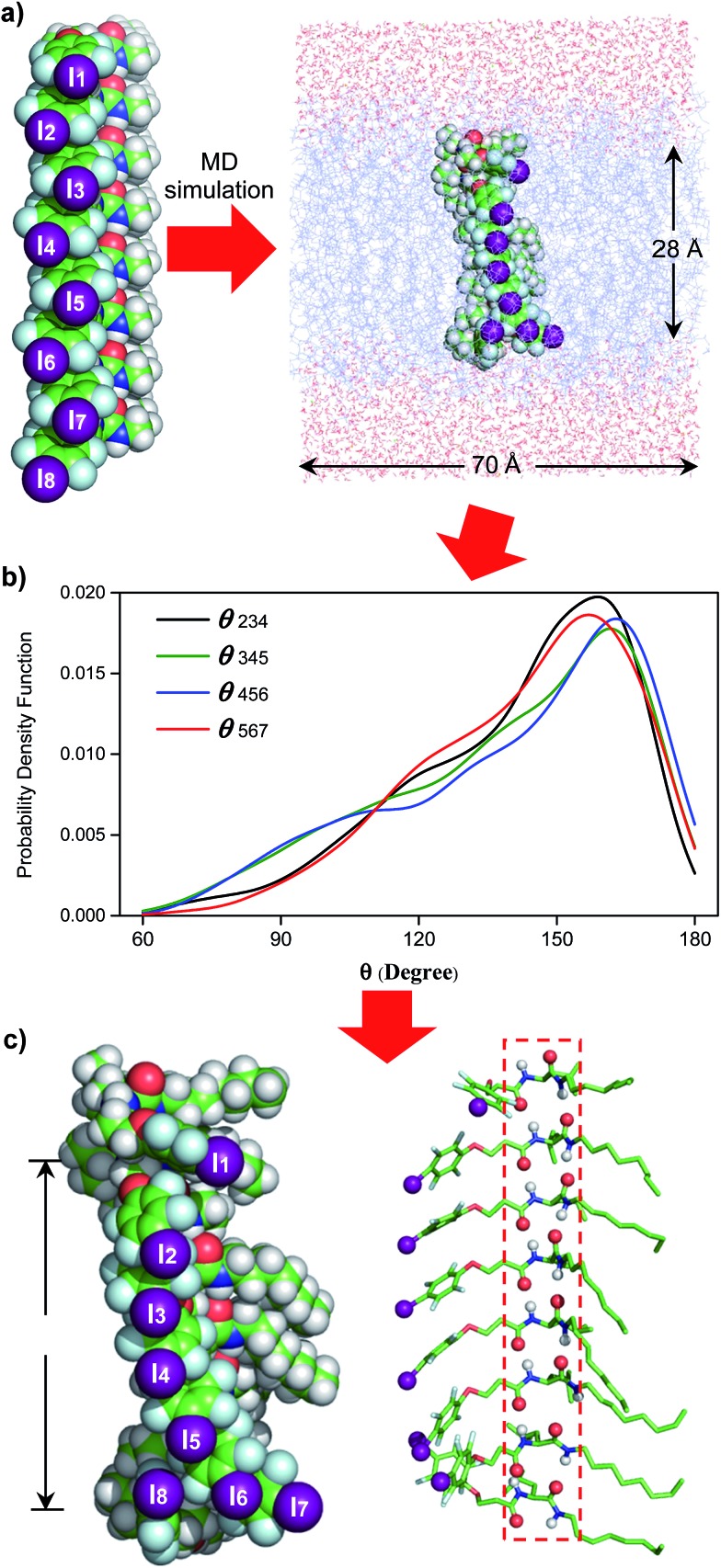
(a) Embedding computationally optimized H-bonded **L8** inside a simulation box of 70 Å (w) × 70 Å (w) × 74 Å (h), comprising 128 POPC molecules and 4794 water molecules. (b) Probability distribution patterns of *θ*_234_, *θ*_345_, *θ*_456_ and *θ*_567_ obtained after analysing 1000 structures. (c) A representative highly populated structure with the four *θ* angles approaching those having the highest probabilities shown in (b). POPC = 1-palmitoyl-2-oleoyl-*sn-glycero*-3-phosphocholine.

Additional comparisons among **L8** and **2–4** convincingly demonstrate the importance of (1) the co-existence of two amide bonds that likely allow for a tighter and more directional stacking of channel molecules (**L8***vs.***2**), (2) the side chain of leucine for more efficient stacking (**2***vs.***3**) and (3) the amide bond in forming a H-bonded structure for ordered spanning of the hydrophobic membrane region (**3***vs.***4**). These summative findings are also in accordance with the molecular dynamics (MD) simulation results (Fig. 4).

### Anion selectivity in chloride transport

With high activity exhibited by these chloride transporters, our subsequent investigations focused on evaluating and comparing anion selectivity for the most efficient anion channels formed by **A10**, **L8** and **L10**. For this purpose, both intra- and extravesicular anions were kept the same (100 mM NaX, X^–^ = Cl^–^, Br^–^, I^–^, NO_3_^–^ and ClO_4_^–^) with a proton gradient of pH 7 (inside) to pH 8 (outside). Data compiled in Fig. 3f, S9 and S10† show that **A10** with an EC_50_ value of 9.4 μM exhibits good anion selectivity in chloride transport, and channels **L8** and **L10** likely selectively transport non-chloride anions (Fig. S10†).

### Chloride transport through a channel mechanism

Single channel current traces for chloride transport, recorded in a planar lipid bilayer at various voltages including –100 mv in symmetric baths (*cis* chamber = *trans* chamber = 1 M KCl, Fig. 3g and S11†), unambiguously confirm that **L8**-mediated chloride transport occurs *via* a channel, rather than a carrier mechanism. On the basis of the fitted linear current–voltage (*I*–*V*) plot (Fig. 3h and S10†), the Cl^–^ conductance (*γ*_Cl^–^_) of **L8** was found to be 586 ± 11 fS.

To shed some light on the possible structural features of one-dimensionally aligned channel molecules in the lipid membrane, MD simulation using the CHARMM program,10a–e PME method10*f* and SHAKE algorithm10*g* was performed on **L8**. In particular, the H-bonded 1D structure, which consists of eight molecules of **L8** (528 atoms), was first computationally optimized using the COMPASS force field10*h* and then was embedded in a bilayer of 128 POPC molecules (17 152 atoms) solvated on two sides by 2 × 2397 water molecules (Fig. 4a). This leads to a simulation system of 32 062 atoms with a dimension of 70 Å (w) × 70 Å (w) × 74 Å (h). After equilibration steps, the production run was carried out for 30 ns. The last 20 ns trajectories with 1000 structural snapshots were used for analysing both the hydrophobic thickness of the POPC membrane and the distribution probability of angle *θ* (*e.g.*, the angle formed by the three immediately adjacent iodine atoms such as iodine atoms 2–4, Fig. 4a and b) using a probability density function.10*i*

To estimate the hydrophobic membrane thickness, the *Z*-coordinates along the membrane normal of all 254 ester O-atoms from 64 POPC molecules located either at the top or on the bottom layers were averaged. The separation distance between the two averaged *Z*-coordinates of O-atoms from the top and bottom layers was calculated with deduction of a van der Waals diameter of 3.1 Å for the O-atom. The same calculation was performed for all 1000 structural snapshots to derive the probability distribution of the hydrophobic membrane thickness (Fig. S12†). From these computations, 633 structures have a hydrophobic thickness of 27 to 29 Å, and the average thickness over 1000 structures is 28.1 Å, a value that could be corroborated by the experimentally and computationally determined values of 27.1 and 27.8 Å, respectively, for the POPC membrane.^11^

Embedding eight molecules of **L8** in the lipid membrane shows that the first and eighth molecules are not aligned well with the central six molecules (Fig. 4a and c). The most highly populated angles for *θ*_234_, *θ*_345_, *θ*_456_ and *θ*_567_ are 159°, 162°, 163° and 157°, respectively, with a representative structure, closely capturing these angles, shown in Fig. 4c. In this highly populated structure, the largest intermolecular separation distance along the *Z* axis among the eight iodine atoms occurs between the 1^st^ and 7^th^ iodine atoms (25 Å). Consistent with the intermolecular separation of 5.0 Å in **Fmoc-Phe-C4** in the solid state (Fig. 1a)7*a* and of 4.9 Å in the computationally optimized H-bonded **L8** (Fig. 1c), this separation distance of 25 Å suggests that six or seven molecules might be sufficient to span the hydrophobic core distance of 28.1 Å.

Through these MD simulations, the most important point to note is that, regardless of angle *θ* of varying magnitudes, all eight molecules of **L8** remain H-bonded to each other in all 1000 structures surveyed (Fig. 4c). This demonstrates the reliability of intermolecular H-bonds in linking six or seven anion-binding molecules together to form a self-assembled 1D pathway, which spans the hydrophobic membrane region to facilitate highly efficient anion transport across the membrane.

### High activity in chloride transport

The use of halogen bonds to mediate anion transport was first explored by Matile,2b,c,3i with compounds **5** (EC_50_ = 3.1 μM)2*c* and **8** (EC_50_ = 0.88 μM in terms of chloride binding units)3*i* as the most active carrier and channel molecules among their own categories, respectively. These EC_50_ values, including those of **6** (260 μM) and **7** (68 μM) presented in Table 1, were determined using cholesterol-free LUVs with a total lipid concentration of 31.3 μM, while our assay conditions contain lipid and cholesterol at concentrations of 73.9 and 36.9 μM, respectively. For a fair comparison, we have therefore re-determined the EC_50_ values of **A10**, **L8** and **L10** using Matile's conditions (100 mM NaCl, 10 mM HEPES, pH gradient from 7 to 8, and the total lipid concentration without cholesterol = 31.3 μM).2b,c,3i

**Table 1:** EC_50_ values of channels **A10**, **L8** and **L10** as well as other halogen bond-mediated chloride carriers (**5–7**) or channels (**8**)^*a*^

	**A10**	**L8**	**L10**	**5** ^*b*^	**6** ^*c*^	**7** ^*d*^	**8** ^*e*^
EC_50_ (μM)	2.37	0.39	0.93	3.6	260	68	0.88
*n* value^*f*^	3.5	3.6	3.1	3.3	3.6	1.9	0.8

^*a*^Determined using Matile's conditions (100 mM NaCl, 10 mM HEPES, pH gradient from 7 to 8, and the total lipid concentration without cholesterol = 31.25 μM; see ref. 2b, c and 3i).

^*b*^EC_50_ value from ref. 2c is 3.1 μM with a Hill coefficient of 3.3.

^*c*^From ref. 2c.

^*d*^From ref. 2b.

^*e*^From ref. 3i with the value normalized by the authors on the basis of eight halogen-binding units contained in channel **8**.

^*f*^Hill coefficient.

To confirm that we are able to produce LUVs with properties similar to those prepared by Matile and his co-workers under the same assay conditions, we have performed Hill analysis on commercially available compound **5**, and obtained an EC_50_ value of 3.6 μM with a Hill coefficient of 3.3 (Fig. S13†). These values are similar or identical to the reported values of EC_50_ (3.1 μM) and the Hill coefficient (3.3).2*c* Under these assay conditions, the EC_50_ values of **A10**, **L8** and **L10** were 
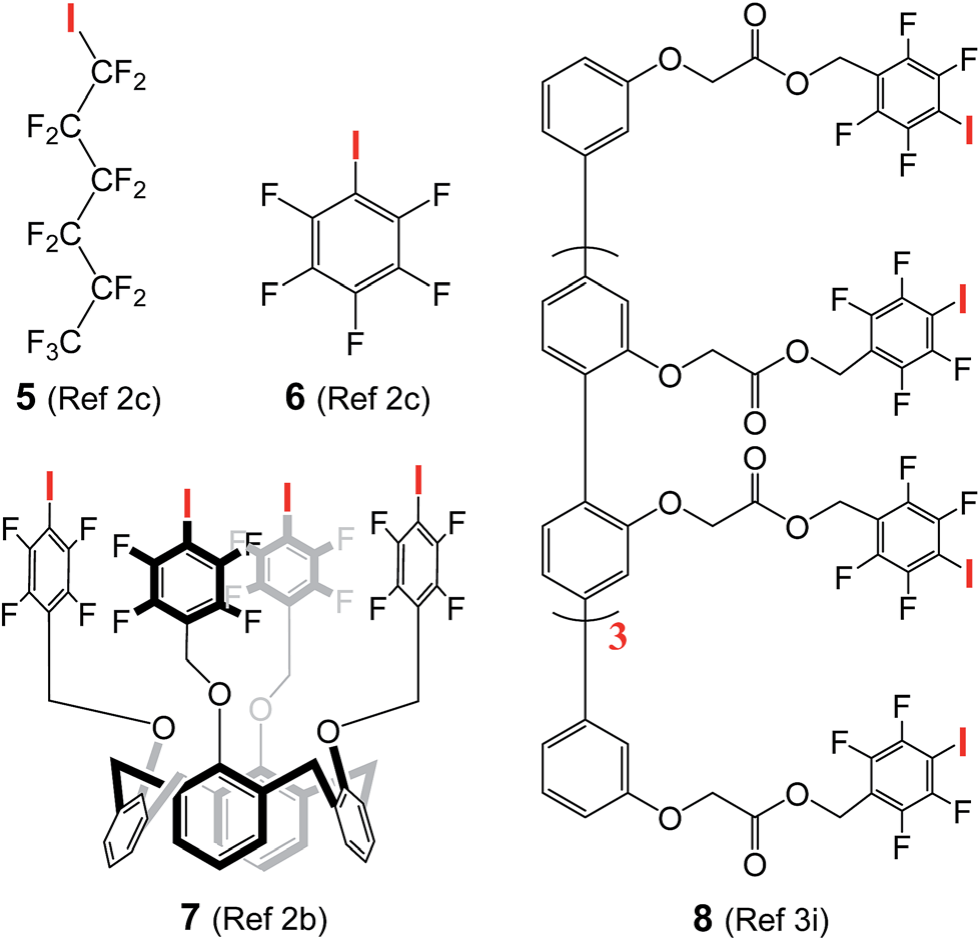
determined to be 2.37, 0.39 and 0.93 μM (Table 1 and Fig. S13†), respectively. The most active **L8** is 8.2 and 1.2 times more active than carrier **5** (3.6 μM) and unimolecular channel molecule **8** (0.88 μM in terms of effective chloride-binding units). When normalized based on the molecular weight, the ion transport activity of **L8** is about 6.5 and 1.6 times those of **5** and **8**, respectively. That **L8** is more potent than unimolecular channel **8** is a clear indication of the excellent ability of the non-covalently associated peptidic scaffold in inducing linearly arrayed iodine atoms into a conformation that is more conducible to chloride transport than the rod-like scaffold in **8**.

In addition, chloride carrier **5**, when evaluated under our assay conditions containing 33 mol% cholesterol that makes membrane less fluid,^12^ shows a moderate transport activity of 38% at 40 μM, and such moderate activity remains unchanged even when the concentration increases to 100 μM (Fig. S14†). These values suggest the ion transport activity of **5** to be at least 10 times lower than that of **L8** having an EC_50_ value of 3.6 μM in the cholesterol-containing environment.

### High anticancer activity

**Fig. 5:**
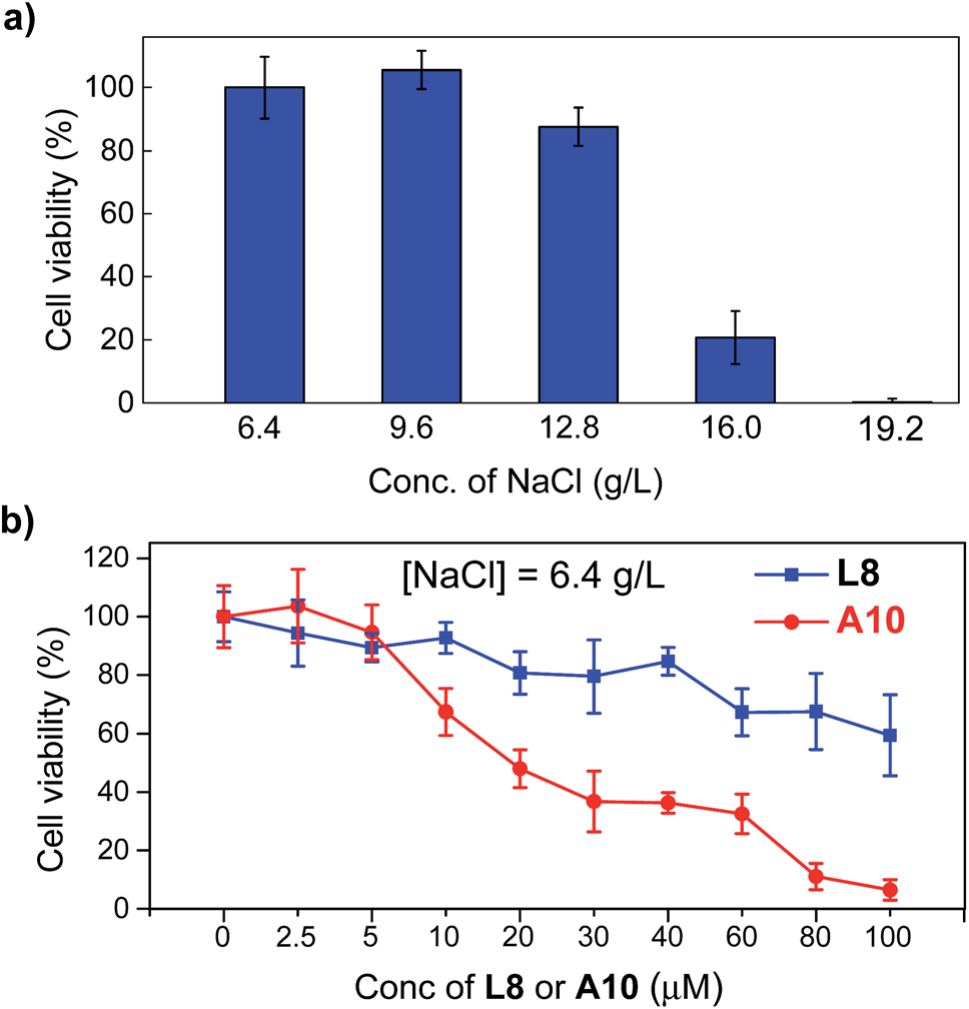
(a) Sensitivity of human breast cancer cells BT-474 toward five different concentrations of NaCl from 6.4 to 19.2 g mL^–1^ after 24 h. (b) Viabilities of BT-474 cells in the presence of various concentrations of **L8** and **A10** at [NaCl] = 6.4 g L^–1^.

The above verified highly efficient chloride transport through a channel mechanism displayed by these artificial anion channels prompted us to examine the possibility of their uses in cancer chemotherapy.4f,^5^ Human breast cancer cells (BT-474, obtained from the American Type Culture Collection, USA) were cultured in Dulbecco's Modified Eagle Medium in the presence of up to 19.2 g L^–1^ NaCl (6.4 g L^–1^ is the typical concentration for cell growth) with concentrations of **L8** or **A10** varying from 0 to 100 μM (Fig. 5 and S15†). The viabilities of cells, determined after culturing the cells for 1 day at 37 °C with 5% CO_2_ in the absence of channel molecules, show that BT-474 cells are sensitive to the sodium chloride concentration gradient (Fig. 5a). That is, compared to cell viability at [NaCl] = 6.4 g L^–1^, 12, 79 and 99% fewer cells survive at [NaCl] of 12.8, 16.0 and 19.2 g L^–1^, respectively. Continued testing shows that **A10** is considerably more potent than **L8** in inhibiting cell growth across all three different concentrations of NaCl (*e.g.*, 6.4, 9.6 and 12.8 g L^–1^) with an IC_50_ value of 20 μM for **A10** at [NaCl] = 6.4 g L^–1^ (>100 μM for **L8**, Fig. 5b and S15†). For comparison, highly effective anticancer agent cisplatin has an IC_50_ value of 37 μM against the same BT-474 cells.^13^ Assuming all **A10** molecules associate to form channels with each channel comprising six or more such molecules, the IC_50_ value in terms of effective channel concentration is lower than 3.3 μM.

## Conclusions

In conclusion, we have developed a class of artificial chloride channels with high activity and good selectivity *via* directional self-assembly of polarized electron-deficient iodine atoms. While these linearly arrayed acidic iodine atoms, which form a series of chloride-binding and -transporting sites, are responsible for facilitating chloride transport likely *via* a multi-ion jumping mode, the high modularity of the monopeptide backbone enables rapid optimization of the transport activity and selectivity of channels in a combinatorial format. The identified channels **A10**, **L8** and **L10** all turn out to be very active with the best EC_50_ values for chloride transport reaching 0.39 μM (1.2 mol% relative to lipid) and 3.6 μM (3.2 mol% relative to lipid/cholesterol) in cholesterol-free and -containing LUVs. In particular, the highly active **A10** exhibits not only a fractional transport activity for chloride much better than other monovalent anions including bromide, but also an excellent inhibitory activity toward human breast cancer cells with an IC_50_ value of 20 μM. Further refinement to enhance the anion selectivity and cytotoxicity of channels toward cancer cells is possible and expected.

## Conflicts of interest

There are no conflicts to declare.

## Supplementary Material

Supplementary information
