# Supplementary material for: A halogen bond-mediated highly active artificial chloride channel with high anticancer activity
Source: Chem Sci. 2018 Mar 15;9(17):4044–51. doi: 10.1039/c8sc00602d (PMC5935034; doi:10.1039/c8sc00602d)
Supplement: Supplementary file 1 [file SC-009-C8SC00602D-s001.pdf]

Electronic Supplementary Material (ESI) for Chemical Science.
This journal is © The Royal Society of Chemistry 2018

# Supporting Information

## A halogen bond-mediated highly selective and active artificial chloride channel with high anticancer activity

Changliang Ren,[a] Xin Ding,[a] Arundhati Roy,[a] Jie Shen,[a] Shaoyuan Zhou,[b] Feng Chen,[a] Sam Fong Yau Li,[c] Haisheng Ren,[b] Yi Yan Yang[a] and Huaqiang Zeng[a,]*

[a] Institute of Bioengineering and Nanotechnology, 31 Biopolis Way, The Nanos, Singapore 138669

[b] College of Chemical Engineering, Sichuan University, Chengdu, China 610065

[c] NUS Environmental Research Institute, Department of Chemistry, 3 Science Drive 3, National University of Singapore, Singapore 117543

**General Remarks** …………S2

**Synthetic Scheme and Chemical Structures of Channel Library** …………S3

**Experimental Procedures and Compound Characterizations** …………S3

**SEM Images of Fibers Formed by A10, L8 and L10** …………S11

**Ion Transport Study and the $EC_{50}$ Measurement using HPTS Assay** …………S12

**Cation Selectivity using HPTS Assay** …………S15

**Ion Transport Mechanism by SPQ Assay** …………S16

**Ion Transport Mechanism by FCCP Assay** …………S18

**Ion Transport Mechanism by Valinomycin Assay** …………S19

**$^{19}$F NMR Titration Experiments with TBACl** …………S20

**Anion Selectivity Using HPTS Assay** …………S21

**Single Channel Current Measurement in Planar Lipid Bilayers** …………S23

**Dynamic Hydrophobic Membrane Thickness of POPC Membrane** …………S24

**$EC_{50}$ Values for A10, L8 and L10 using Cholesterol-Free LUVs** …………S26

**$EC_{50}$ Determination for 5 using Cholesterol-Containing LUVs** …………S28

**Determination of Cancer Cell Viability via MTT Assay** …………S29

**$^{1}$H and $^{13}$C NMR Spectra** …………S30

## General Remarks

All the reagents were obtained from commercial suppliers and used as received unless otherwise noted. Aqueous solutions were prepared from MilliQ water. The organic solutions from all liquid extractions were dried over anhydrous $Na_2SO_4$ for a minimum of 15 minutes before filtration. Flash column chromatography was performed using pre-coated 0.2 mm silica plates from Selecto Scientific. Chemical yield refers to pure isolated substances. $^{1}H$, $^{13}C$ and $^{19}F$ NMR spectra were recorded on a Bruker ACF-400 spectrometer. For $^{1}H$ NMR, the solvent signal of $CDCl_3$ was referenced at $\delta$ = 7.26 ppm. Coupling constants ($J$ values) are reported in Hertz (Hz). $^{1}H$ NMR data are recorded in the order: chemical shift value, multiplicity (s, singlet; d, doublet; t, triplet; q, quartet; m, multiplet; br, broad), number of protons that gave rise to the signal and coupling constant, where applicable. $^{13}C$ spectra are proton-decoupled and recorded on Bruker ACF400 (400 MHz). The solvent, $CDCl_3$, was referenced at $\delta$= 77 ppm. For $^{19}F$ NMR, chemical shifts were referenced against 1,4-diflurobenzen at -120.50 ppm. $CDCl_3$ (99.8%-Deuterated) and $D_2O$ (99.9%-Deuterated) were purchased from Aldrich and used without further purification. Mass spectra were acquired with Shimazu LCMS-2010EV. Scanning electron microscopy (SEM) images were obtained on a JEOL JSM-7400F electron microscope (5 kV). Single channel current measurements in planar lipid bilayers were carried out using Planar Lipid Bilayer Workstation (Warner Instruments, Hamden, CT).

## Synthetic Scheme and Chemical Structures of Channel Library

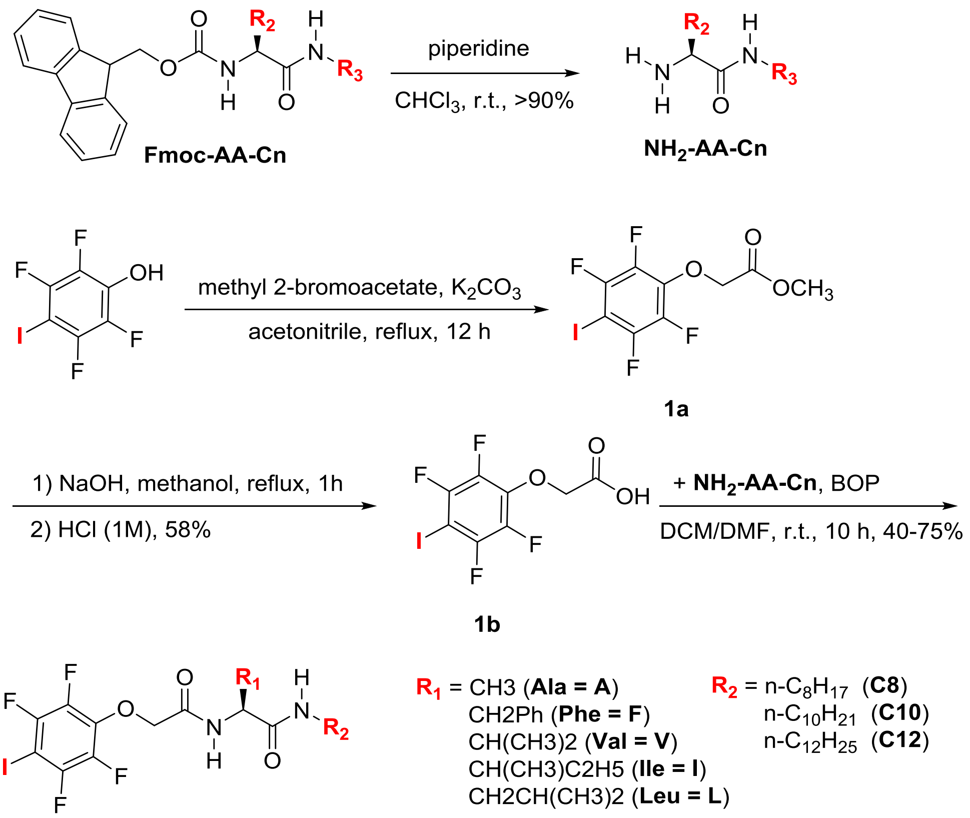


# Experimental Procedures and Compound Characterizations

**For synthesis of starting materials Fmoc-AA-Cn,** see: Ren, C. L.; Ng, G. H. B.; Wu, H.; Chan, K.-H.; Shen, J.; Teh, C.; Ying, J. Y.; Zeng , H. Q. ***Chem. Mater.*, 2016**, ***28***, 4001-4008

## $NH_2$-Phe-C8

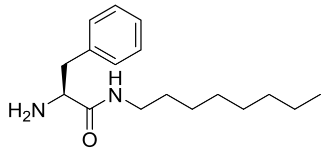

**$NH_2$-Phe-C8**

To a solution of **Fmoc-Phe-C8** (2.0 g, 4 mmol) in $CHCl_3$ (20 mL) was added piperidine (2.0 mL), and reaction was allowed to stir at room temperature for 12 h. The solvent was then removed in *vacuo* and the crude product was purified by flash column chromatography (MeOH:$CH_2Cl_2$ = 1:20, v:v) to afford compound **$NH_2$-Phe-C8** as a pale yellow solid. Yield: 1.0 g, 91%. $^1$H NMR (400 MHz, $CDCl_3$) δ 7.40 – 7.18 (m, 6H), 3.64 (dd, $J$ = 9.2, 4.3 Hz, 1H), 3.36 – 3.22 (m, 3H), 2.73 (dd, $J$ = 13.7, 9.2 Hz, 1H), 1.81 (s, 2H), 1.56 – 1.44 (m, 2H), 1.34 – 1.26 (m, 10H), 0.90 (dd, $J$ = 8.7, 5.0 Hz, 3H). $^{13}$C NMR (100 MHz, $CDCl_3$) δ 173.91, 137.90, 129.34, 128.70, 126.81, 56.45, 41.00, 39.16, 31.83, 29.56, 29.29, 29.23, 26.96, 22.68, 14.15. MS-ESI: calculated for $[M+Na]^+$ ($C_{17}H_{28}ON_2Na$)：*m/z* 299.21, found: *m/z* 299.10.

**2-(2,3,5,6-tetrafluoro-4-iodophenoxy)acetic acid (1b)**

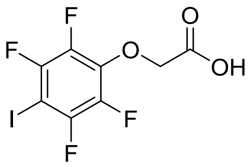

**1b**

2,3,5,6-tetrafluoro-4-iodophenol (4.87 g, 16.7 mmol) was dissolved in acetonitrile (80 mL) to which anhydrous $K_2CO_3$ (4.61 g, 33.4 mmol) and methyl 2-bromoacetate (2.38 mL, 25.1 mmol) were added. The mixture was heated under reflux for 12 hours. The reaction mixture was then filtered and the solvent was removed in *vacuo*. The residue was dissolved in $CH_2Cl_2$ (150 mL), washed with water (3 x 100 mL), and dried over anhydrous $Na_2SO_4$. Removal of $CH_2Cl_2$ in *vacuo* gave the crude product **1a**, which was directly used in the next step without further purification. **1a** was dissolved in methanol (80 mL) to which 1M NaOH (22.5 mL, 22.5 mmol) was added. The mixture was heated under reflux for 1 hour and then the reaction solvent was removed in *vacuo* to yield a white solid, which was dissolved in water and neutralized with 1 M HCl (40 mL) to yield crude product **1b**, which was subjected to column purification (ethyl acetate:n-hexane = 1:20, v/v) to yield compound **1b** as a white solid. Yield: 3.39 g, 58%. $^1$H NMR (400 MHz, $CDCl_3$) δ 9.31 (s, 1H), 4.92 (t, *J* = 0.9 Hz, 2H). $^{13}$C NMR (100 MHz, $CDCl_3$) δ 173.52, 147.32 (dddd, *J* = 244.4, 13.2, 6.5, 4.3 Hz), 141.36 - 141.11 (m), 138.86 – 138.61 (m), 136.53 (tt, *J* = 12.3, 3.2 Hz), 68.73 (t, *J* = 4.0 Hz), 64.78 (t, *J* = 28.1 Hz). MS-ESI: calculated for $[M-H]^-$($C_8H_2O_3F_4I$): *m/z* 348.90, found: *m/z* 348.80.

**F8**

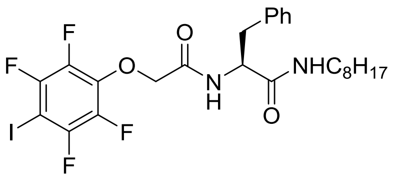

**F8**

**1b** (312 mg, 1.0 mmol), **$NH_2$-Phe-C8** (276 mg, 1.0 mmol) and BOP (486 mg, 1.1 mmol) were dissolved in $CH_2Cl_2$/DMF (8 mL:2 mL) to which diisopropylamine (0.39 ml, 2.2 mmol) was added. The reaction mixture was stirred for 10 hours at room temperature. Solvent was removed in *vacuo* and the crude product was dissolved in $CH_2Cl_2$ (30 mL), and washed with water (2 x 40 mL), which was recrystallized from acetonitrile to to yield compound **F8** as a white solid. Yield: 395 mg, 65%. $^1$H NMR (400 MHz, $CDCl_3$) δ 7.36 – 7.25 (m, 6H), 5.50 (s, 1H), 4.77 – 4.50 (m, 3H), 3.20 – 2.98 (m, 4H), 1.32 – 1.13 (m, 12H), 0.88 (t, *J* = 6.9 Hz, 3H). $^{13}$C NMR (100 MHz, $CDCl_3$) δ 169.70, 166.57, 147.32 (dddd, *J* = 245.2, 13.0, 6.3, 4.2 Hz), 141.53 - 141.29 (m), 139.03 – 138.78 (m), 136.65 (tt, *J* = 12.3, 3.2 Hz), 129.27, 128.77, 127.20, 72.60, 72.57, 72.53, 65.90 (t, *J* = 28.1 Hz), 54.58, 39.65, 38.72, 31.80, 29.25, 29.19, 29.18, 26.77, 22.67, 14.14. MS-ESI: calculated for $[M+H]^+$ ($C_{25}H_{30}O_3N_2F_4I$): *m/z* 609.12, found: *m/z* 609.10.

**Preparation of channel molecules follows the same synthetic procedure as F8.**

**F10**

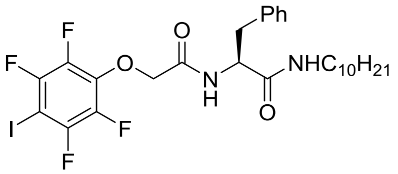


F10

$^1$H NMR (400 MHz, $CDCl_3$) δ 7.44 – 7.25 (m, 6H), 5.51 (t, *J* = 5.5 Hz, 1H), 4.76 – 4.57 (m, 3H), 3.28 – 2.96 (m, 4H), 1.34 – 1.10 (m, 16H), 0.86 (t, *J* = 6.9 Hz, 3H). $^{13}$C NMR (100 MHz, $CDCl_3$) δ 169.70, 166.57, 147.32 (dddd, *J* = 245.2, 13.0, 6.3, 4.2 Hz), 141.53 - 141.28 (m), 139.03 – 138.76 (m), 136.65 (tt, *J* = 12.3, 3.2 Hz), 136.34, 129.27, 128.90, 128.77, 127.19, 72.59, 72.56, 72.53, 65.90 (t, *J* = 28.1 Hz), 54.57, 39.65, 38.73, 31.92, 29.57, 29.52, 29.34, 29.24, 26.78, 22.72, 14.17. MS-ESI: calculated for $[M+H]^+$ ($C_{27}H_{34}O_3N_2F_4I$): *m/z* 637.16, found: *m/z* 637.15.

**F12**

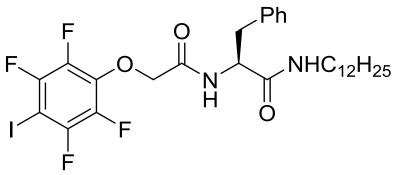


F12

$^1$H NMR (400 MHz, $CDCl_3$) δ 7.38 – 7.22 (m, 5H), 5.56 (t, *J* = 5.7 Hz, 1H), 4.74 – 4.55 (m, 3H), 3.24 – 2.97 (m, 4H), 1.32 – 1.11 (m, 16H), 0.88 (t, *J* = 6.9 Hz, 3H). $^{13}$C NMR (100 MHz, $CDCl_3$) δ 169.71, 166.57, 147.33 (dddd, *J* = 245.2, 13.0, 6.3, 4.2 Hz), 141.53 - 141.31 (m), 139.03 – 138.78 (m), 136.65 (tt, *J* = 12.3, 3.2 Hz), 129.27, 128.76, 127.19, 72.58, 72.55, 72.52, 65.89 (t, *J* = 28.1 Hz), 54.56, 39.65, 38.74, 31.95, 29.69, 29.67, 29.62, 29.57, 29.53, 29.49, 29.39, 29.25, 26.78, 22.73, 14.18. MS-ESI: calculated for $[M+H]^+$ ($C_{29}H_{38}O_3N_2F_4I$): *m/z* 665.19, found: *m/z* 665.20.

**A8**

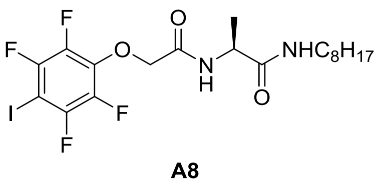


A8

$^1$H NMR (400 MHz, CDCl3) δ 7.37 (d, *J* = 7.5 Hz, 1H), 6.27 (q, *J* = 5.5, 4.8 Hz, 1H), 4.67 (d, *J* = 1.9 Hz, 2H), 4.54 (p, *J* = 7.0 Hz, 1H), 3.25 (qd, *J* = 7.1, 3.2 Hz, 2H), 1.53 – 1.42 (m, 5H), 1.34 – 1.18 (m, 10H), 0.86 (t, *J* = 6.3 Hz, 3H). $^{13}$C NMR (100 MHz, $CDCl_3$) δ 171.28, 166.61, 147.33 (dddd, *J* = 245.3, 13.0, 6.2, 4.2 Hz), 141.60 - 141.36 (m), 139.10 – 138.85 (m), 136.75 (tt, *J* = 12.3, 3.2 Hz), 72.69, 72.66, 72.63, 65.97 (t, *J* = 28.1 Hz), 48.63, 39.75, 31.81, 29.44, 29.24, 29.22, 26.87, 22.66, 18.57, 14.13. MS-ESI: calculated for $[M+H]^+$ ($C_{19}H_{26}O_3N_2F_4I$): *m/z* 533.09, found: *m/z* 533.05.

**A10**

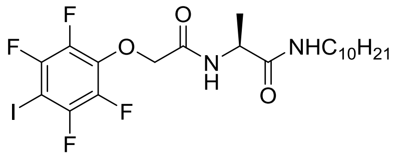

A10

$^1$H NMR (400 MHz, $CDCl_3$) δ 7.38 (d, *J* = 7.6 Hz, 1H), 6.30 (t, *J* = 5.7 Hz, 1H), 4.66 (d, *J* = 1.9 Hz, 2H), 4.54 (p, *J* = 7.0 Hz, 1H), 3.24 (tdd, *J* = 7.1, 5.6, 3.6 Hz, 2H), 1.46 (dd, *J* = 20.1, 7.0 Hz, 5H), 1.25 (d, *J* = 11.7 Hz, 14H), 0.86 (t, *J* = 6.8 Hz, 3H). $^{13}$C NMR (100 MHz, $CDCl_3$) δ 171.28, 166.60, 147.34 (dddd, *J* = 245.4, 13.2, 6.4, 4.3 Hz), 141.61 - 141.36 (m), 139.11 – 138.86 (m), 136.74 (tt, *J* = 12.3, 3.2 Hz), 72.66, 72.63, 72.59, 66.01 (t, *J* = 28.1 Hz), 48.61, 39.73, 31.91, 29.57, 29.43, 29.33, 29.29, 26.88, 22.71, 18.56, 14.16. MS-ESI: calculated for $[M+H]^+$ ($C_{21}H_{30}O_3N_2F_4I$): *m/z* 561.12, found: *m/z* 561.10.

**A12**

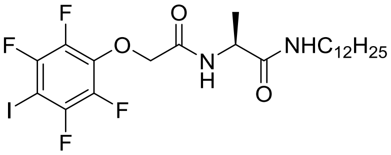

A12

$^1$H NMR (400 MHz, CDCl3) δ 7.30 (d, *J* = 7.7 Hz, 1H), 6.08 (t, *J* = 5.5 Hz, 1H), 4.67 (s, 2H), 4.52 (q, *J* = 7.1 Hz, 1H), 3.26 (ddt, *J* = 10.7, 7.4, 3.9 Hz, 2H), 1.53 – 1.44 (m, 5H), 1.30 - 1.20 (m, 18H), 0.87 (t, *J* = 6.7 Hz, 3H). $^{13}$C NMR (100 MHz, $CDCl_3$) δ 171.18, 166.61, 148.68, 148.63, 148.61, 148.57, 147.24 (dddd, *J* = 245.4, 13.2, 6.4, 4.3 Hz), 141.60 - 141.35 (m), 139.10 – 138.85 (m), 136.75, (tt, *J* = 12.3, 3.2 Hz), 72.71, 72.67, 72.64, 65.96 (t, *J* = 28.1 Hz), 48.63, 39.75, 31.94, 29.68, 29.66, 29.62, 29.57, 29.45, 29.39, 29.29, 26.87, 22.73, 18.43, 14.18. MS-ESI: calculated for $[M+H]^+$ ($C_{23}H_{34}O_3N_2F_4I$): *m/z* 589.16, found: *m/z* 589.10.

**I8**

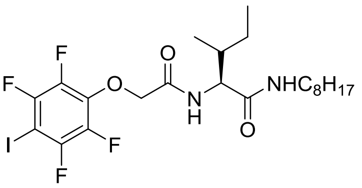

I8

$^1$H NMR (400 MHz, $CDCl_3$) δ 7.24 (s, 1H), 5.86 (t, *J* = 5.7 Hz, 1H), 4.77 – 4.62 (m, 2H), 4.27 (dd, *J* = 8.9, 7.1 Hz, 1H), 3.41 – 3.12 (m, 2H), 1.93 (dd, *J* = 6.5, 3.3 Hz, 1H), 1.53 (dtd, *J* = 14.7, 9.1, 7.2, 3.7 Hz, 3H), 1.31 – 1.10 (m, 11H), 0.97 – 0.84 (m, 9H). $^{13}$C NMR (100 MHz, $CDCl_3$) δ 170.14, 166.69, 146.88 (dddd, *J* = 245.3, 13.1, 6.4, 4.3 Hz), 141.59 - 141.36 (m), 139.09 – 138.85 (m), 136.81 (tt, *J* = 12.3, 3.2 Hz), 72.76, 72.72, 72.69, 65.91 (t, *J* = 28.1 Hz), 57.61, 39.66, 37.33, 31.80, 29.47, 29.22, 26.90, 25.01, 22.66, 15.43, 14.13, 11.30. MS-ESI: calculated for $[M+H]^+$ ($C_{22}H_{32}O_3N_2F_4I$): *m/z* 575.14, found: *m/z* 575.10.

**I10**

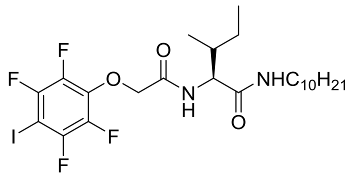


**I10**

$^{1}$H NMR (400 MHz, $CDCl_3$) δ 7.28 (d, *J* = 8.9 Hz, 1H), 6.01 (t, *J* = 5.8 Hz, 1H), 4.77 – 4.62 (m, 2H), 4.29 (dd, *J* = 8.9, 7.2 Hz, 1H), 3.39 – 3.12 (m, 2H), 1.92 (ddt, *J* = 10.2, 6.9, 3.5 Hz, 1H), 1.59 – 1.46 (m, 3H), 1.28 - 1.09 (m, 15H), 0.96 – 0.83 (m, 9H). $^{13}$C NMR (100 MHz, $CDCl_3$) δ 170.18, 166.70, 147.33 (dddd, *J* = 245.3, 13.1, 6.4, 4.3 Hz), 141.59 - 141.34 (m), 139.09 - 138.84 (m), 136.81 (tt, *J* = 12.3, 3.2 Hz), 72.72, 72.69, 72.65, 65.88 (t, *J* = 28.1 Hz), 57.59, 39.67, 37.34, 31.94, 29.67, 29.61, 29.57, 29.45, 29.39, 29.27, 26.91, 25.01, 22.73, 15.42, 14.17, 11.29. MS-ESI: calculated for $[M+H]^+$ ($C_{24}H_{36}O_3N_2F_4I$): *m/z* 603.17, found: *m/z* 603.15.

**I12**

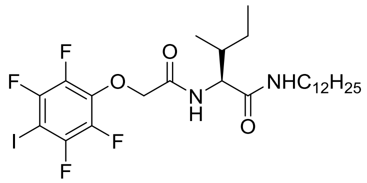


**I12**

$^{1}$H NMR (400 MHz, $CDCl_3$) δ 7.28 (s, 1H), 5.91 (t, *J* = 5.7 Hz, 1H), 4.77 – 4.60 (m, 2H), 4.28 (dd, *J* = 8.9, 7.1 Hz, 1H), 3.26 (ddt, *J* = 36.9, 13.0, 6.3 Hz, 2H), 1.98 – 1.86 (m, 1H), 1.52 (dddd, *J* = 18.9, 14.1, 9.0, 5.2 Hz, 3H), 1.42 – 1.04 (m, 19H), 1.00 – 0.86 (m, 9H). $^{13}$C NMR (100 MHz, $CDCl_3$) δ 170.13, 166.69, 147.77 (dddd, *J* = 245.3, 13.1, 6.4, 4.3 Hz), 141.60 - 141.35 (m), 139.09 – 138.85 (m), 136.81 (tt, *J* = 12.3, 3.2 Hz), 72.79, 72.76, 65.92 (t, *J* = 28.1 Hz), 57.62, 39.67, 37.32, 31.96, 31.91, 29.74, 29.56, 29.48, 29.40, 29.33, 29.26, 26.91, 25.02, 22.72, 15.44, 14.17, 11.31. MS-ESI: calculated for $[M+H]^+$ ($C_{26}H_{40}O_3N_2F_4I$): *m/z* 631.20, found: *m/z* 631.20.

**L8**

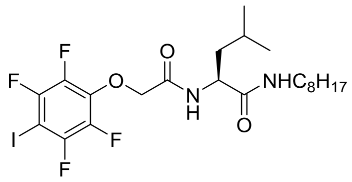


**L8**

$^{1}$H NMR (400 MHz, $CDCl_3$) δ 7.13 (d, *J* = 8.5 Hz, 1H), 6.21 (s, 1H), 4.76 – 4.59 (m, 2H), 4.49 (td, *J* = 8.3, 6.0 Hz, 1H), 3.34 – 3.13 (m, 2H), 1.77 – 1.56 (m, 3H), 1.49 (dt, *J* = 14.1, 7.0 Hz, 2H), 1.32 – 1.21 (m, 10H), 0.94 (t, *J* = 6.0 Hz, 6H), 0.87 (t, *J* = 6.3 Hz, 3H). $^{13}$C NMR (100 MHz, $CDCl_3$) δ 171.03, 166.77, 147.33 (dddd, *J* = 245.2, 13.0, 6.4, 4.3 Hz), 141.58 - 141.33 (m), 139.08 – 138.83 (m), 136.73 (tt, *J* = 12.3, 3.2 Hz), 72.66, 72.63, 72.59, 65.97 (t, *J* = 28.1 Hz), 51.49, 41.20, 39.69, 31.80, 29.41, 29.22, 26.87, 24.81, 22.82, 22.67, 22.28, 14.13. MS-ESI: calculated for $[M+H]^+$ ($C_{22}H_{32}O_3N_2F_4I$): *m/z* 575.14, found: *m/z* 575.10.

**L10**

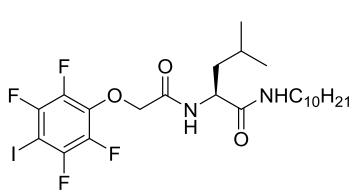

L10

$^{1}$H NMR (400 MHz, $CDCl_3$) δ 7.05 (d, *J* = 8.5 Hz, 1H), 5.95 (s, 1H), 4.77 – 4.60 (m, 2H), 4.54 – 4.33 (m, 1H), 3.26 (h, *J* = 6.7 Hz, 2H), 1.81 – 1.62 (m, 3H), 1.49 (d, *J* = 7.2 Hz, 2H), 1.28 (d, *J* = 7.0 Hz, 14H), 0.96 (d, *J* = 5.3 Hz, 6H), 0.87 (t, *J* = 6.7 Hz, 3H). $^{13}$C NMR (100 MHz, $CDCl_3$) δ 170.94, 166.79, 147.25 (dddd, *J* = 245.3, 13.1, 6.4, 4.3 Hz), 141.56 - 141.35 (m), 139.09 – 138.85 (m), 136.71 (tt, *J* = 12.3, 3.2 Hz), 72.72, 72.68, 72.65, 66.04 (t, *J* = 28.1 Hz), 51.49, 41.09, 39.71, 31.92, 29.56, 29.43, 29.34, 29.27, 26.86, 24.81, 22.83, 22.72, 22.27, 14.17. MS-ESI: calculated for $[M+H]^+$ ($C_{24}H_{36}O_3N_2F_4I$)： *m/z* 603.17, found: *m/z* 603.15.

**L12**

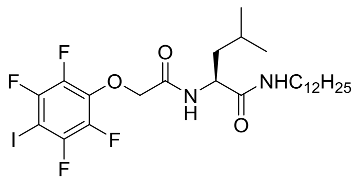

L12

$^{1}$H NMR (400 MHz, $CDCl_3$) δ 7.07 (d, *J* = 8.6 Hz, 1H), 6.02 (d, *J* = 6.8 Hz, 1H), 4.67 (dd, *J* = 9.3, 3.9 Hz, 2H), 4.53 – 4.36 (m, 1H), 3.24 (dt, *J* = 12.2, 6.2 Hz, 2H), 1.80 – 1.50 (m, 6H), 1.26 (d, *J* = 16.5 Hz, 18H), 0.99 – 0.89 (m, 6H), 0.86 (t, *J* = 4.1 Hz, 3H). $^{13}$C NMR (100 MHz, $CDCl_3$) δ 170.92, 166.75, 147.32 (dddd, *J* = 245.3, 13.1, 6.4, 4.3 Hz), 141.59 - 141.32 (m), 139.06 – 138.85 (m), 136.69 (tt, *J* = 12.3, 3.2 Hz), 72.66, 66.01 (t, *J* = 28.1 Hz), 51.47, 41.09, 39.69, 31.92, 29.64, 29.57, 29.39, 29.25, 26.85, 24.79, 22.82, 22.71, 22.25, 14.15. MS-ESI: calculated for $[M+H]^+$ ($C_{26}H_{40}O_3N_2F_4I$)： *m/z* 631.20, found: *m/z* 631.20.

**V8**

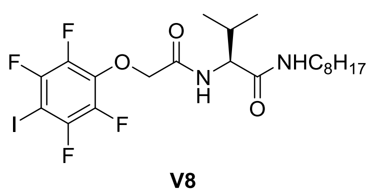

V8

$^{1}$H NMR (400 MHz, $CDCl_3$) δ 7.29 (s, 1H), 5.93 (d, *J* = 5.9 Hz, 1H), 4.80 – 4.58 (m, 2H), 4.25 (dd, *J* = 8.9, 6.9 Hz, 1H), 3.36 – 3.11 (m, 2H), 2.26 – 2.07 (m, 1H), 1.49 (q, *J* = 7.2 Hz, 2H), 1.34 – 1.20 (m, 10H), 0.98 (dd, *J* = 6.8, 3.7 Hz, 6H), 0.87 (t, *J* = 6.3 Hz, 3H). $^{13}$C NMR (100 MHz, $CDCl_3$) δ 170.12, 166.77, 147.51 (dddd, *J* = 245.2, 13.0, 6.3, 4.2 Hz), 141.60 - 141.35 (m), 139.10 – 138.85 (m), 136.83 (tt, *J* = 12.3, 3.2 Hz), 72.76, 72.73, 72.70, 65.93 (t, *J* = 28.1 Hz), 58.45, 39.66, 31.79, 31.14, 29.48, 29.21, 26.90, 22.66, 19.23, 18.19, 14.13. MS-ESI: calculated for $[M+H]^+$ ($C_{21}H_{30}O_3N_2F_4I$)： *m/z* 6561.12, found: *m/z* 561.10.

**V10**

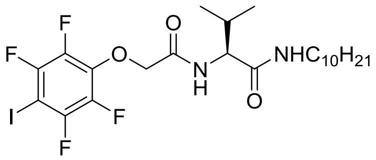


V10

$^{1}$H NMR (400 MHz, $CDCl_3$) δ 7.30 (s, 1H), 5.99 (t, *J* = 5.7 Hz, 1H), 4.79 – 4.61 (m, 2H), 4.25 (dd, *J* = 8.9, 7.0 Hz, 1H), 3.41 – 3.13 (m, 2H), 2.28 – 2.11 (m, 1H), 1.49 (q, *J* = 7.1 Hz, 2H), 1.26 (d, *J* = 11.6 Hz, 14H), 0.98 (dd, *J* = 6.8, 3.6 Hz, 6H), 0.88 (t, *J* = 6.3 Hz, 3H). $^{13}$C NMR (100 MHz, $CDCl_3$) δ 170.13, 166.77, 147.33 (dddd, *J* = 245.2, 13.0, 6.3, 4.2 Hz), 141.60 - 141.35 (m), 139.09 – 138.85 (m), 136.83 (tt, *J* = 12.3, 3.2 Hz), 72.75, 72.72, 72.69, 65.91 (t, *J* = 28.1 Hz), 58.44, 39.67, 31.91, 31.16, 29.56, 29.48, 29.33, 29.26, 26.91, 22.71, 19.23, 18.20, 14.16. MS-ESI: calculated for $[M+H]^+$ ($C_{23}H_{34}O_3N_2F_4I$): *m/z* 589.16, found: *m/z* 589.15.

**V12**

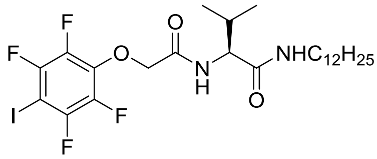


V12

$^{1}$H NMR (400 MHz, $CDCl_3$) δ 7.29 (d, *J* = 8.9 Hz, 1H), 5.99 (s, 1H), 4.77 – 4.61 (m, 2H), 4.25 (dd, *J* = 8.9, 7.0 Hz, 1H), 3.40 – 3.10 (m, 2H), 2.24 – 2.10 (m, 1H), 1.50 (t, *J* = 7.0 Hz, 2H), 1.26 (d, *J* = 18.2 Hz, 18H), 0.98 (dd, *J* = 6.8, 3.6 Hz, 6H), 0.87 (t, *J* = 6.3 Hz, 3H). $^{13}$C NMR (100 MHz, $CDCl_3$) δ 170.13, 166.78, 147.39 (dddd, *J* = 245.2, 13.0, 6.3, 4.2 Hz), 141.60 - 141.35 (m), 139.10 – 138.85 (m), 136.83 (tt, *J* = 12.3, 3.2 Hz), 72.76, 72.72, 72.69, 65.92 (t, *J* = 28.1 Hz), 58.45, 39.67, 31.94, 31.14, 29.67, 29.61, 29.56, 29.48, 29.38, 29.26, 26.91, 22.73, 19.23, 18.20, 14.17. MS-ESI: calculated for $[M+H]^+$ ($C_{25}H_{38}O_3N_2F_4I$): *m/z* 617.19, found: *m/z* 617.15.

**1**

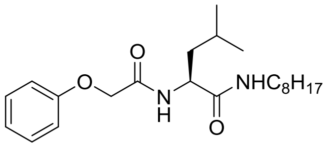


1

$^{1}$H NMR (400 MHz, $CDCl_3$) δ 7.27 – 7.23 (m, 1H), 6.95 (dd, *J* = 10.6, 4.2 Hz, 2H), 6.85 (dd, *J* = 8.7, 0.9 Hz, 2H), 6.26 (s, 1H), 4.50 – 4.35 (m, 3H), 3.24 – 3.06 (m, 2H), 1.72 - 1.60 (m, 1H), 1.57 – 1.48 (m, 2H), 1.45 - 1.37 (m, 2H), 1.25 - 1.15 (m, 10H), 0.90 – 0.76 (m, 9H). $^{13}$C NMR (100 MHz, $CDCl_3$) δ 171.30, 168.38, 157.04, 129.80, 122.22, 114.68, 67.10, 51.33, 41.03, 39.65, 31.81, 29.44, 29.24, 29.23, 26.89, 24.75, 22.86, 22.67, 22.21, 14.14. MS-ESI: calculated for $[M+H]^+$ ($C_{22}H_{37}O_3N_2$): *m/z* 377.28, found: *m/z* 377.20.

**2**

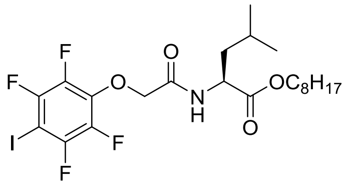

**2**

$^{1}$H NMR (400 MHz, $CDCl_3$) δ 7.06 (d, *J* = 8.4 Hz, 1H), 4.78 – 4.57 (m, 3H), 4.13 (td, *J* = 6.7, 0.7 Hz, 2H), 1.73 – 1.60 (m, 5H), 1.38 – 1.23 (m, 10H), 0.96 (d, *J* = 6.2 Hz, 6H), 0.87 (t, *J* = 6.9 Hz, 3H). $^{13}$C NMR (101 MHz, $CDCl_3$) δ 172.35, 166.43, 147.36 (dddd, *J* = 245.2, 13.0, 6.3, 4.3 Hz), 141.76 – 141.26 (m), 139.29 – 138.81 (m), 136.84 (tt, *J* = 12.4, 3.4 Hz), 72.83, 72.80, 72.77, 65.95 (t, *J* = 28.1 Hz), 65.67, 50.56, 41.65, 31.78, 29.18, 29.16, 28.49, 25.84, 24.94, 22.78, 22.66, 22.03, 14.12. MS-ESI: calculated for $[M+H]^+$ ($C_{22}H_{31}O_4NF_4I$): *m/z* 576.12, found: *m/z* 576.15.

**3**

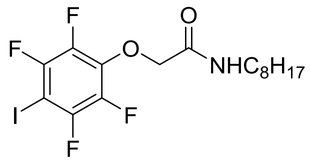

**3**

$^{1}$H NMR (400 MHz, $CDCl_3$) δ 6.68 (s, 1H), 4.67 (s, 2H), 3.36 (dd, *J* = 13.2, 7.0 Hz, 2H), 1.61 – 1.55 (m, 2H), 1.29 (dd, *J* = 12.8, 6.0 Hz, 10H), 0.88 (t, *J* = 6.9 Hz, 3H). $^{13}$C NMR (100 MHz, $CDCl_3$) δ 166.52, 147.44 (dddd, *J* = 245.3, 13.0, 6.3, 4.2 Hz), 141.80 – 141.40 (m), 139.12 - 138.94 (m), 136.88 - 136.75 (m), 73.06, 73.02, 72.99, 65.91 (t, *J* = 28.1 Hz), 39.24, 31.79, 29.44, 29.23, 29.21, 26.85, 22.67, 14.13. MS-ESI: calculated for $[M+Na]^+$ ($C_{16}H_{20}O_3NF_4INa$): *m/z* 484.04, found: *m/z* 484.05.

**4**

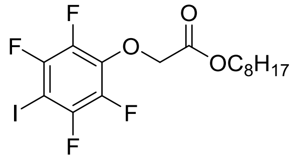

**4**

$^{1}$H NMR (400 MHz, CDCl3) δ 4.85 (s, 2H), 4.18 (t, J = 6.7 Hz, 2H), 1.69 – 1.61 (m, 2H), 1.31 – 1.21 (m, 10H), 0.88 (t, J = 6.9 Hz, 3H). $^{13}$C NMR (100 MHz, CDCl3) δ 167.91, 147.34 (dddd, *J* = 245.2, 13.0, 6.3, 4.3 Hz), 141.46 – 141.33 (m), 139.01 - 138.80 (m), 136.89 (tt, *J* = 12.4, 3.4 Hz), 69.37, 69.33, 69.29, 65.94, 64.25 (t, *J* = 28.1 Hz), 31.78, 29.18, 29.16, 28.45, 25.79, 22.67, 14.13. MS-ESI: calculated for $[M+H]^+$ ($C_{16}H_{20}O_3F_4I$): *m/z* 463.04, found: *m/z* 462.85.

## SEM Images of Fibers Formed by **A10**, **L8** and **L10**

The nanofibers formed by **A10**, **L8** and **L10** were prepared by dissolving 2.5% w/v (mg/100 μL) of samples in hot n-hexane and subsequently cooling to room temperature under ambient condition. A small amount of as-formed nanofiber was placed on copper tape attached aluminum stub, and allowed to dry overnight under ambient conditions. Later, sample was sputter-coated with a thin layer of Pt, and subjected to SEM observation on a JEOL JSM-7400F electron microscope.

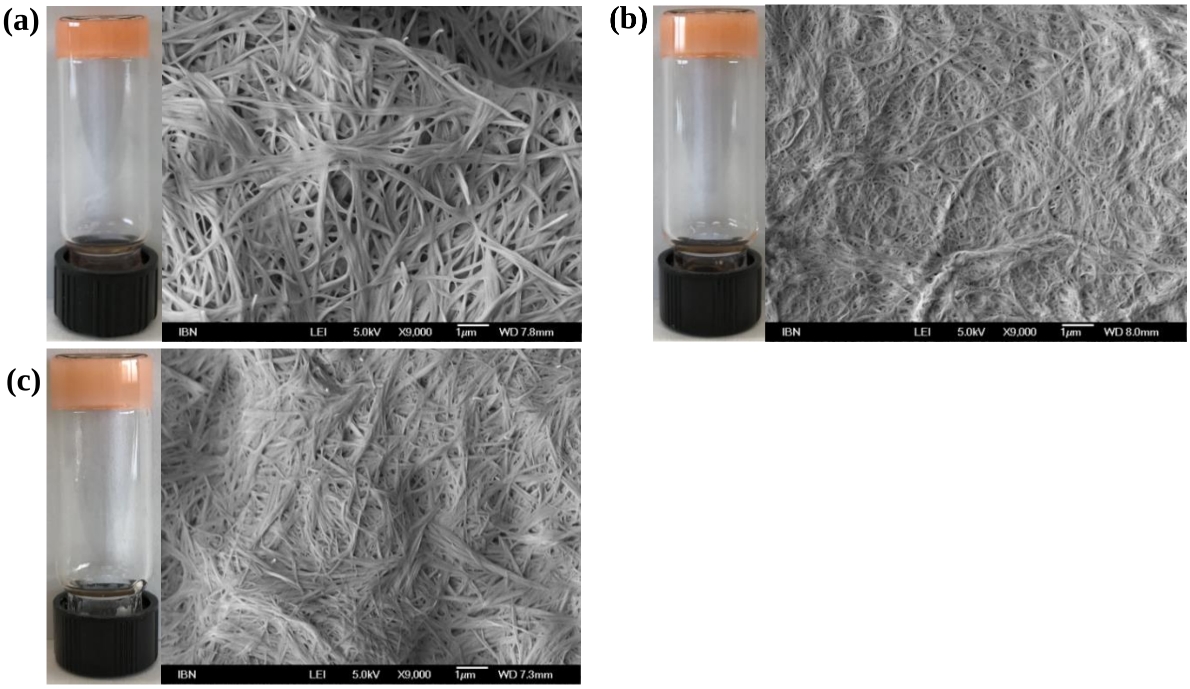


**Figure S1**. SEM micrographs of gels and as-formed nanofibers of (a) **A10**, (b) **L8** and **L10** in n-hexane. (n-Hexane was dyed with 0.002% red Sudan III for clarity in visualization)

## Ion Transport Study and $EC_{50}$ Measurements using HPTS Assay

Egg yolk L-α-phosphatidylcholine (EYPC, 0.6 ml, 25 mg/mL in $CHCl_3$, Avanti Polar Lipids, USA) and cholesterol (3.8 mg) were dissolved in $CHCl_3$ (10 mL). The mixed solvents were removed under reduced pressure at room temperature. After drying the resulting film under high vacuum overnight at room temperature, the film was hydrated with 4-(2-hydroxyethyl)-1-piperazine-ethane sulfonic acid (HEPES) buffer solution (1.5 mL, 10 mM HEPES, 100 mM NaCl, pH = 7.0) containing a pH sensitive dye 8-hydrox-ypyrene-1,3,6-trisulfonic acid (HPTS, 0.1 mM) in thermostatic shaker-incubator at 37 $^{o}$C for 2 hours to give a milky suspension. The mixture was then subjected to 8 freeze-thaw cycles: freezing in liquid $N_2$ for 30 seconds and heating at 37 $^{o}$C for 1.5 minutes. The vesicle suspension was extruded through polycarbonate membrane (0.1 μm) to produce a homogeneous suspension of large unilamellar vesicles (LUVs) of about 150 nm in diameter with HPTS encapsulated inside. The suspension of LUVs was dialyzed for 16 hours with gentle stirring (300 r/min, 4$^{o}$C) using membrane tube (MWCO = 10,000) against the same HEPES buffer solution (300 mL, without HPTS) for 6 times to remove the unencapsulated HPTS to yield LUVs with lipids at a concentration of 13 mM.

The HPTS-containing LUV suspension (10 μL, 13 mM in 10 mM HEPES buffer containing 100 mM NaCl at pH = 7.0) was added to a HEPES buffer solution (1.75 mL, 10 mM HEPES, 100 mM NaCl at pH= 8.0) to create a pH gradient for ion transport study. A solution of channel molecules in DMSO was then injected into the suspension under gentle stirring. Upon the addition of channels, the emission of HPTS was immediately monitored at 510 nm with excitations at both 460 and 403 nm recorded simultaneously for 300 seconds using fluorescence spectrophotometer (Hitachi, Model F-7100, Japan) after which time an aqueous solution of Triton X-100 (30 μL, 20% v/v) was immediately added to achieve the maximum change in fluorescence dye emission. The final transport trace was obtained as a ratiometric value of $I_{460}/I_{403}$ and normalized based on the ratiometric value of $I_{460}/I_{403}$ after addition of triton using the following equation (S1).

$$I_f = [(I_t - I_0)/(I_1 - I_0)] \qquad \text{(S1)}$$

where, $I_f$ = Fractional emission intensity, $It$ = Fluorescence intensity at time $t$, $I_1$ = Fluorescence intensity after addition of Triton X-100, and $I_0$ = Initial fluorescence intensity .

The fractional changes $R_X^-$ was calculated for each curve using the normalized value of $I_{460}/I_{403}$ at 300 seconds before the addition of triton, referring to the ratio of blank as 0 and that of triton as 1. Fitting the fractional transmembrane activity $R_X^-$ vs channel concentration using the Hill equation: $Y=1/(1+(EC_{50}/[C])^n)$ gave the Hill coefficient $n$ and $EC_{50}$ values.

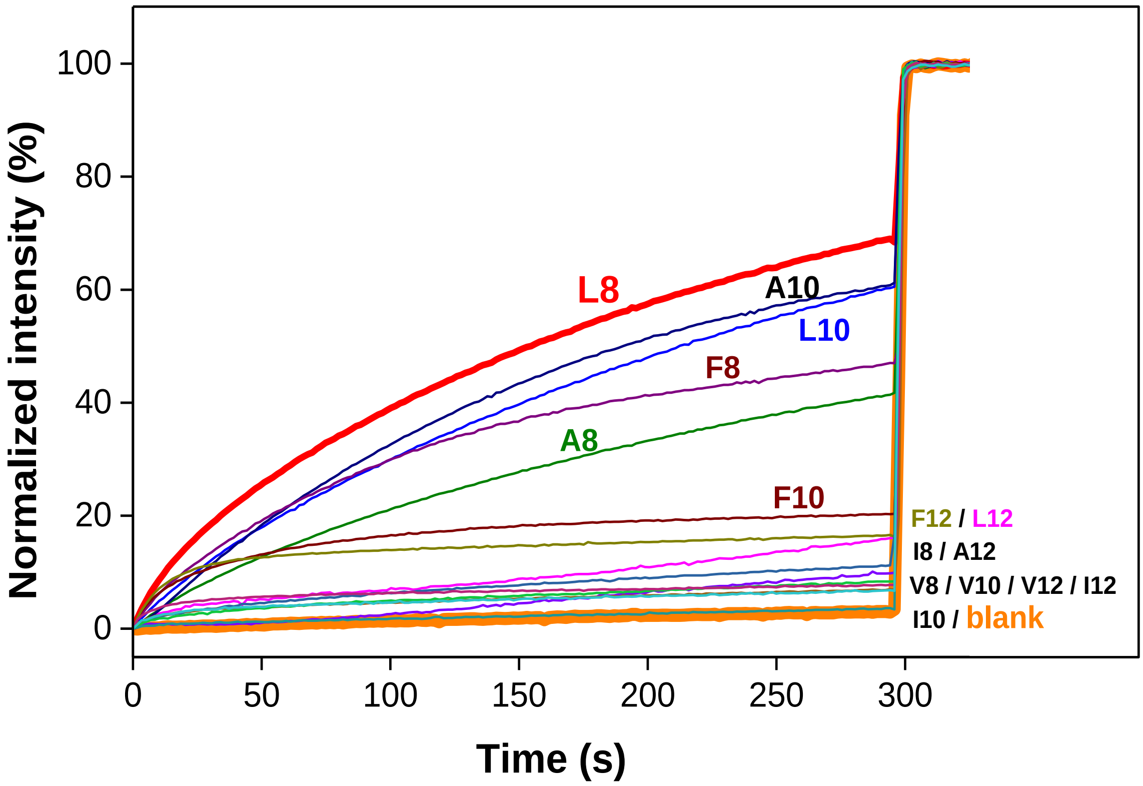


**Figure S2**. $Cl^-$ transport curves at a channel concentration of 10 μM using HPTS assay, illustrating progressively enhanced transport of $Cl^-$ ions upon fine-tuning $R_1$ and $R_2$ groups.

### $EC_{50}$ Determination for **L8**, **L10** and **A10**

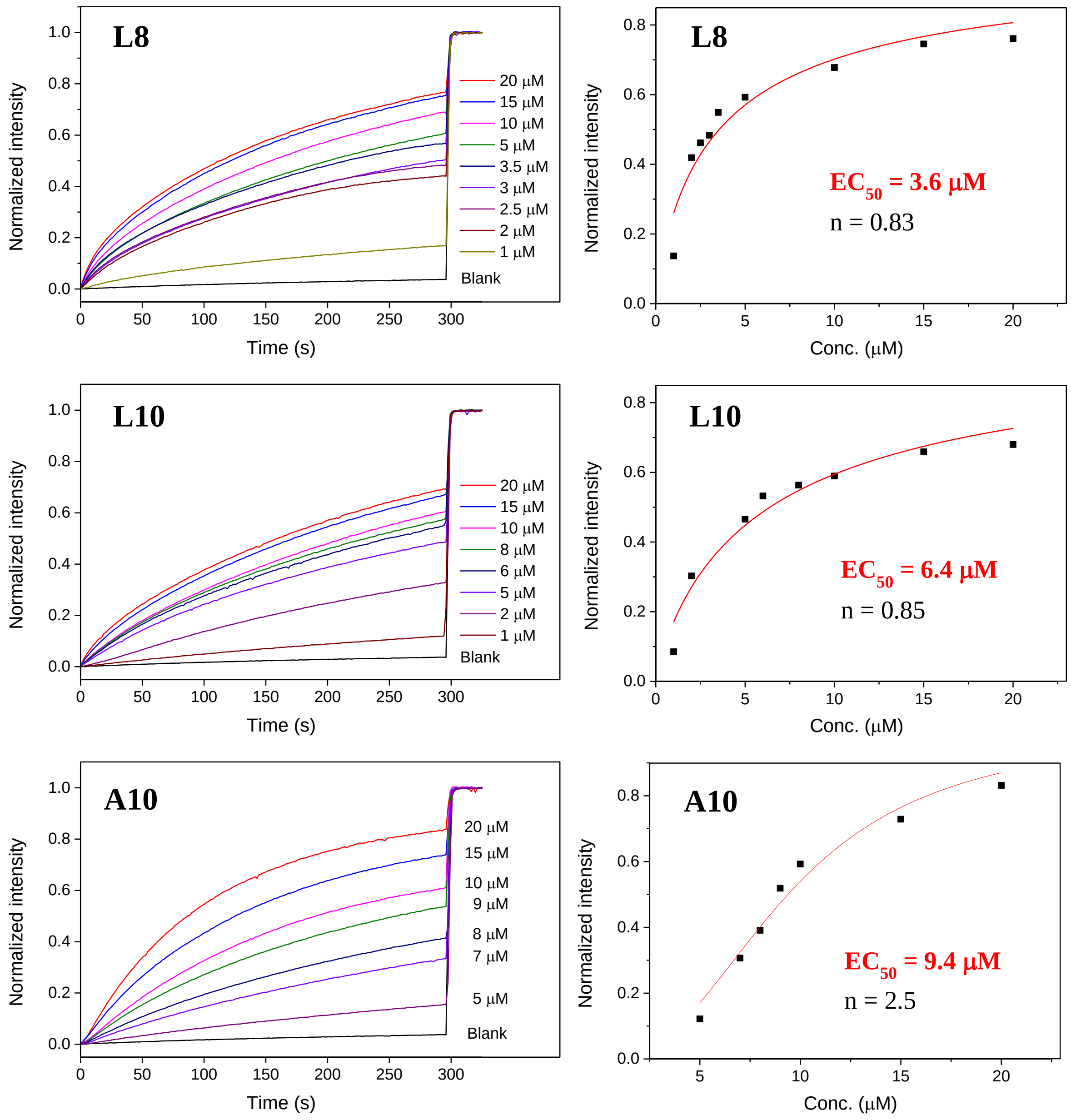


**Figure S3**. Determination of $EC_{50}$ values using the ratiometric values of I460/I403 at different concentrations as a function of time for **L8**, **L10** and **A10** towards $Cl^-$.

**Cation Selectivity using HPTS Assay**

The HPTS-containing LUV suspension (10 μL, 13 mM in 10 mM HEPES buffer containing 100 mM NaCl at pH = 7.0) was added to a HEPES buffer solution (1.75 mL, 10 mM HEPES, 100 mM MCl at pH = 8.0, where $M^{n+}$= $Li^+$, $Na^+$, $K^+$, $Rb^+$, and $Cs^+$) to create a pH gradient for ion transport study. A solution of channel molecule **L8** or **A10** at a final concentration of 3.6 μM or 9.4 μM ($EC_{50}$) in DMSO was then injected into the suspension under gentle stirring. Upon the addition of channels, the emission of HPTS was immediately monitored at 510 nm with excitations at both 460 and 403 nm recorded simultaneously for 300 seconds using fluorescence spectrophotometer (Hitachi, Model F-7100, Japan) after which time an aqueous solution of Triton X-100 (30 μL, 20% v/v) was immediately added to achieve the maximum change in dye fluorescence emission. The final transport trace was obtained as a ratiometric value of $I_{460}/I_{403}$ and normalized based on the ratiometric value of $I_{460}/I_{403}$ after addition of triton.

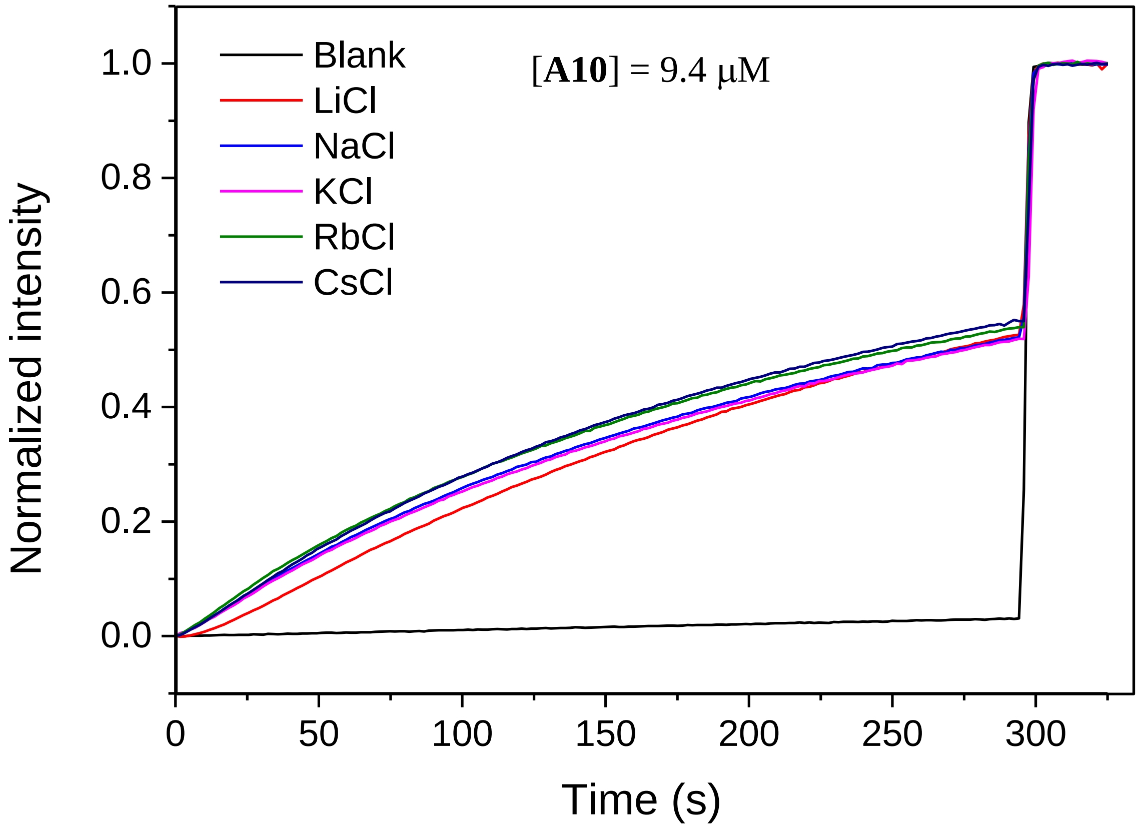


**Figure S4**. Ion transport activities toward $Li^+$, $Na^+$, $K^+$, $Rb^+$ and $Cs^+$ for **A10** at 9.4 μM, indicating the minimal involvement of cations in the pH equilibrium process.

## Ion Transport Mechanism by SPQ Assay

Egg yolk L-α-phosphatidylcholine (EYPC, 0.6 ml, 25 mg/mL in $CHCl_3$, Avanti Polar Lipids, USA) and cholesterol (3.8 mg) were dissolved in $CHCl_3$ (10 mL). The mixed solvents were removed under reduced pressure at room temperature. After drying the resulting film under high vacuum overnight at room temperature, the film was hydrated $NaNO_3$ solution (1.5 mL, 200 mM) containing a $Cl^-$-sensitive dye 6-methoxy-N-(3-sulfopropyl)quinolinium (SPQ) (0.5 mM) in thermostatic shaker-incubator at 37 °C for 2 hours to give a milky suspension. The mixture was then subjected to 8 freeze-thaw cycles: freezing in liquid $N_2$ for 30 seconds and heating at 37 °C for 1.5 minutes. The vesicle suspension was extruded through polycarbonate membrane (0.1 μm) to produce a homogeneous suspension of large unilamellar vesicles (LUVs) of about 150 nm in diameter with SPQ encapsulated inside. The suspension of LUVs was dialyzed for 16 hours with gentle stirring (300 r/min, 4°C) using membrane tube (MWCO = 10,000) against the same $NaNO_3$ buffer solution (200 mM, without SPQ) for 6 times to remove the unencapsulated SPQ to yield LUVs with lipids at a concentration of 13 mM.

The SPQ-containing LUV suspension (10 μL, 13 mM in 200 mM $NaNO_3$) was added to a NaCl solution (1.75 mL, 200 mM) to create an extravesicular chloride gradient. A solution of channel molecule in DMSO at different concentrations was then injected into the suspension under gentle stirring. Upon the addition of channels, the emission of SPQ was immediately monitored at 430 nm with excitations at 360 nm for 300 seconds using fluorescence spectrophotometer (Hitachi, Model F-7100, Japan) after which time an aqueous solution of Triton X-100 (30 μL, 20% v/v) was immediately added to completely destruct the chloride gradient. The final transport trace was obtained by normalizing the fluorescence intensity using the following equation.

$$I_f = [(I_t - I_1)/(I_0 - I_1)]$$

where, $I_f$ = Fractional emission intensity, $It$ = Fluorescence intensity at time $t$, $I_1$ = Fluorescence intensity after addition of Triton X-100, and $I_0$ = Initial fluorescence intensity .

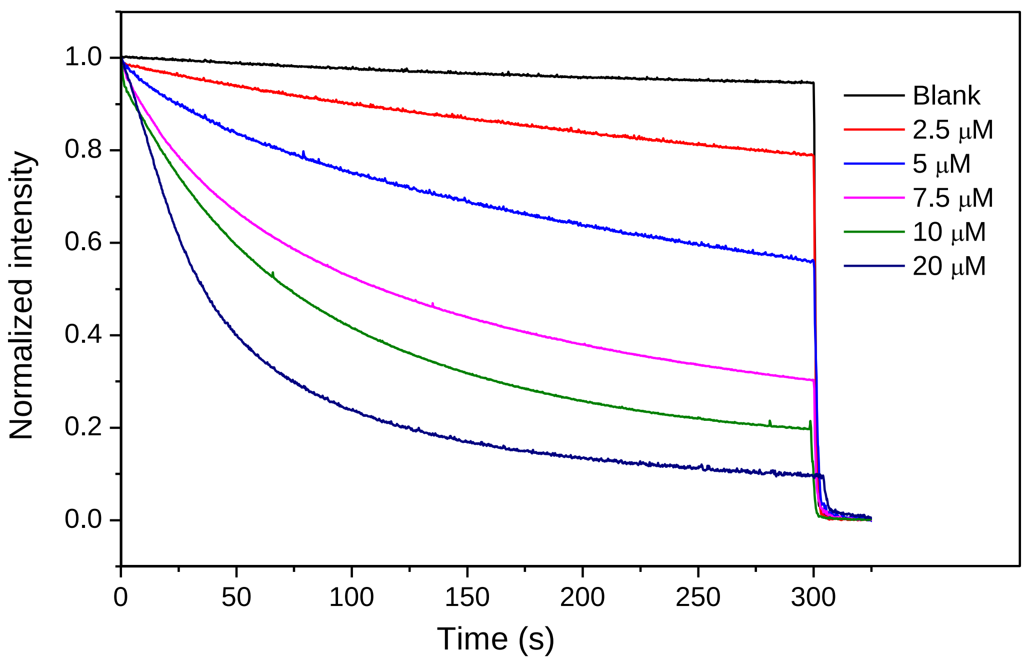


**Figure S5**. Fluorescence intensity change of SQP ($\lambda_{ex}$ = 360 nm, $\lambda_{em}$ = 430 nm) after addition of **A10** at different concentrations. Inside LUV: 200 mM $NaNO_3$, 0.5 mM SPQ. Outside LUV: 200 mM NaCl.

## Ion Transport Mechanism by FCCP Assay

The HPTS-containing LUV suspension (10 μL, 13 mM in 10 mM HEPES buffer containing 100 mM NaCl at pH = 7.0) was added to a HEPES buffer solution (1.75 mL, 10 mM HEPES, 100 mM NaCl) to create a pH gradient for ion transport study. A solution of carbonyl cyanide-4-(trifluoromethoxy)phenylhydrazone (FCCP) (1 μM) and channel molecule **A10** (9.4 μM) or **L8** (3.6 μM) in DMSO was then injected into the suspension under gentle stirring at 20 s and 70 s, respectively. Upon the addition of channels, the emission of HPTS was immediately monitored at 510 nm with excitations at both 460 and 403 nm recorded simultaneously for 300 seconds using fluorescence spectrophotometer (Hitachi, Model F-7100, Japan) after which time an aqueous solution of Triton X-100 (30 μL, 20% v/v) was immediately added to achieve the maximum change in dye fluorescence emission. The final transport trace was obtained as a ratiometric value of $I_{460}/I_{403}$ and normalized based on the ratiometric value of $I_{460}/I_{403}$ after addition of triton.

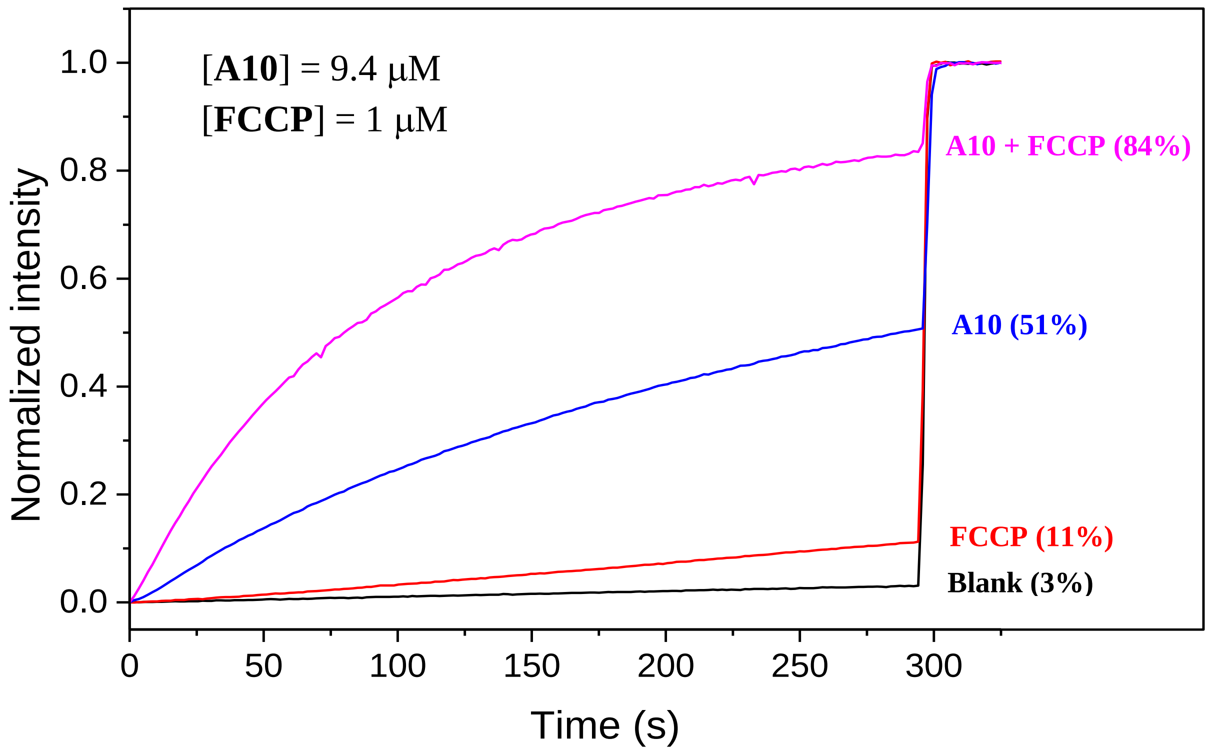


**Figure S6**. Ion transport activities of **A10** (9.4 μM) determined in the absence and in the presence of FCCP (1 μM).

## Ion Transport Mechanism by Valinomycin Assay

The HPTS-containing LUV suspension (10 μL, 13 mM in 10 mM HEPES buffer containing 100 mM NaCl at pH = 7.0) was added to a HEPES buffer solution (1.75 mL, 10 mM HEPES, 100 mM NaCl) to create a pH gradient for ion transport study. A solution of valinomycin (VA) (25 pM) and channel molecule **A10** (9.4 μM) or **L8** (3.6 μM) in DMSO was then injected into the suspension under gentle stirring at 20 s and 70 s, respectively. Upon the addition of channels, the emission of HPTS was immediately monitored at 510 nm with excitations at both 460 and 403 nm recorded simultaneously for 300 seconds using fluorescence spectrophotometer (Hitachi, Model F-7100, Japan) after which time an aqueous solution of Triton X-100 (30 μL, 20% v/v) was immediately added to achieve the maximum change in dye fluorescence emission. The final transport trace was obtained as a ratiometric value of $I_{460}/I_{403}$ and normalized based on the ratiometric value of $I_{460}/I_{403}$ after addition of triton.

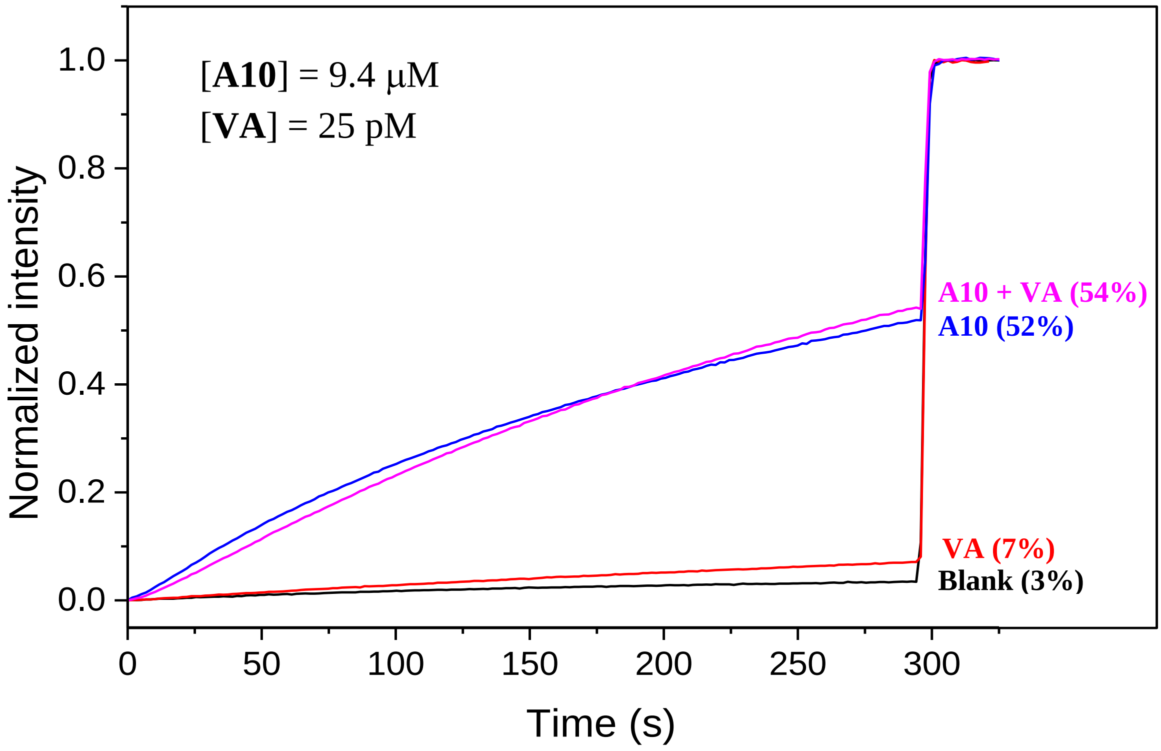


**Figure S7**. Ion transport activities of **A10** (9.4 μM) determined in the absence and in the presence of valinomycin (25 pM).

## $^{19}$F NMR Titration Experiments with TBACl

A $D_2O$-saturated $CDCl_3$ was prepared by mixing 10 mL $CDCl_3$ with 0.6 mL $D_2O$ under votexing conditions for 3 min. This $D_2O$-saturated $CDCl_3$ was used to prepare solutions containing tetrabutylammonium chloride (TBACl) and **L8**. In the $^{19}$F NMR titration experiments, 0 – 20 equiv of TABCl was titrated into a $CDCl_3$ solution containing **L8** at 1 mM at room temperature with 1,4-diflurobenzene (-120.50 ppm, J. Am. Chem. Soc., 2013, 135, 4648) used as the internal standard.

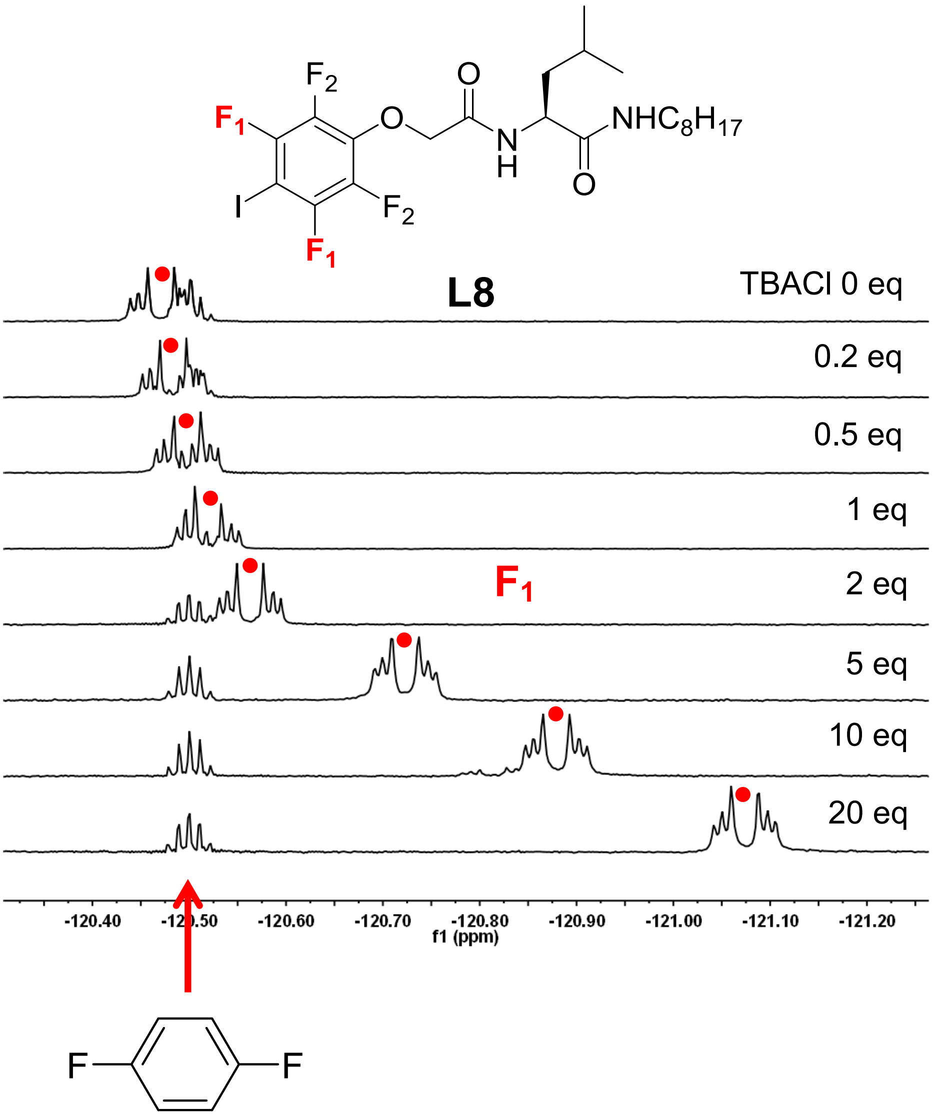


**Figure S8**. $^{19}$F NMR titration experiments involving titrating 0 – 20 equiv. of TBACl into a $D_2O$-saturated $CDCl_3$ containing **L8** at 1 mM. An overall change of 0.61 ppm was observed. 1,4-diflurobenzene (-120.50 ppm) was used as the internal standard.

## Anion Selectivity Using HPTS Assay

The HPTS-containing LUV suspension (10 μL, 13 mM in 10 mM HEPES buffer containing 100 mM NaX at pH = 7.0) was added to a HEPES buffer solution (1.75 mL, 10 mM HEPES, 100 mM NaX, where $X^-$= $Cl^-$, $Br^-$, $I^-$, $NO_3^-$ and $ClO_4^-$ at pH= 8.0) to create a pH gradient for ion transport study. A solution of channel molecule **L8**, **L10** or **A10** at a final concentration of 3.6 μM, 6.4 μM or 9.4 μM ($EC_{50}$) in DMSO was then injected into the suspension under gentle stirring. Upon the addition of channels, the emission of HPTS was immediately monitored at 510 nm with excitations at both 460 and 403 nm recorded simultaneously for 300 seconds using fluorescence spectrophotometer (Hitachi, Model F-7100, Japan) after which time an aqueous solution of Triton X-100 (30 μL, 20% v/v) was immediately added to achieve the maximum change in dye fluorescence emission. The final transport trace was obtained as a ratiometric value of $I_{460}/I_{403}$ and normalized based on the ratiometric value of $I_{460}/I_{403}$ after addition of triton.

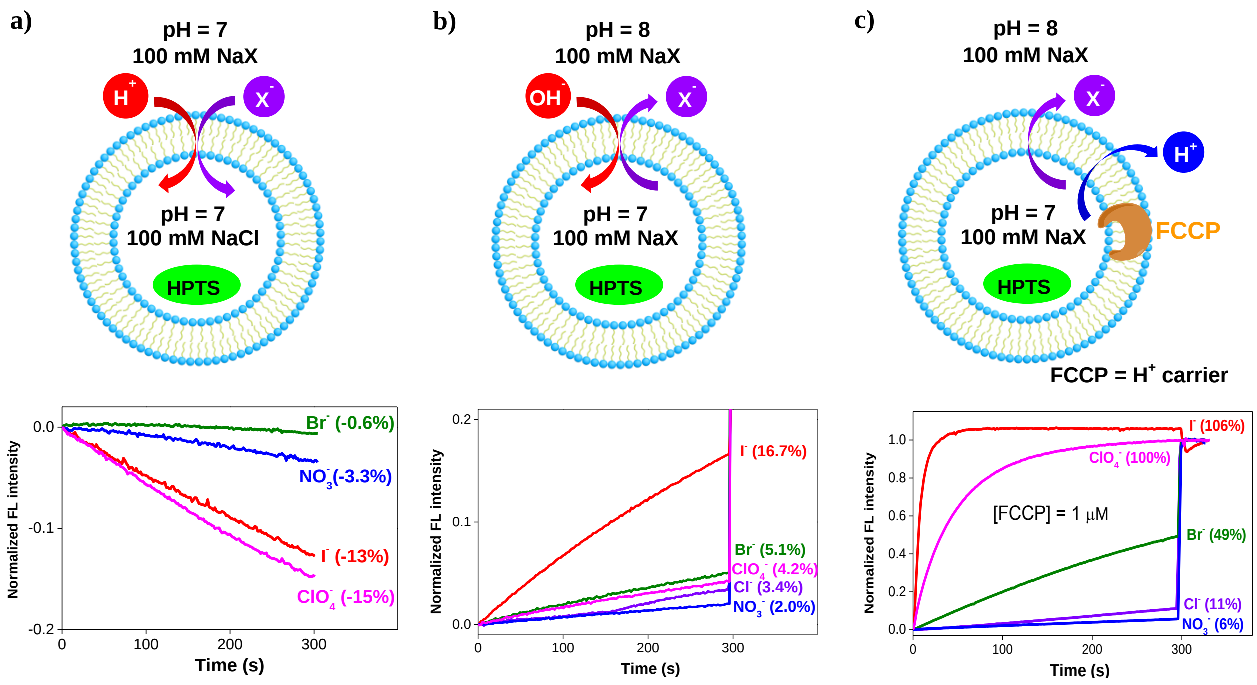


**Figure S9**. Background permeability measured for different anions under two different assay conditions in the absence of channel molecules (a and b) and in the presence of FCCP (c). For Figure S9a, higher permeability of such as iodide ions (relative to chloride) brings protons into LUVs and so lowers down the intravesicular pH. Based on Figure S9b, it can be seen that iodide has higher background membrane permeability than other anions. By using FCCP, which is a proton carrier, it can be further seen that FCCP-mediated proton efflux increase the background permeability of anions to hugely different extents. This might suggest a positive cooperativity between proton efflux and anion's hydrophobicity, and such positive cooperativity takes place to the largest extent for $I^-$, $ClO^-$ and $Br^-$. These experiments therefore suggest that while higher signal observed for $Cl^-$ (Fig. 6a) definitely indicates higher selectivity for $Cl^-$, larger signals seen for $I^-$, $ClO^-$ and $Br^-$ (Fig. 6b and 6c) can't be used to unambiguously confirm the higher selectivity of these anions over $Cl^-$.

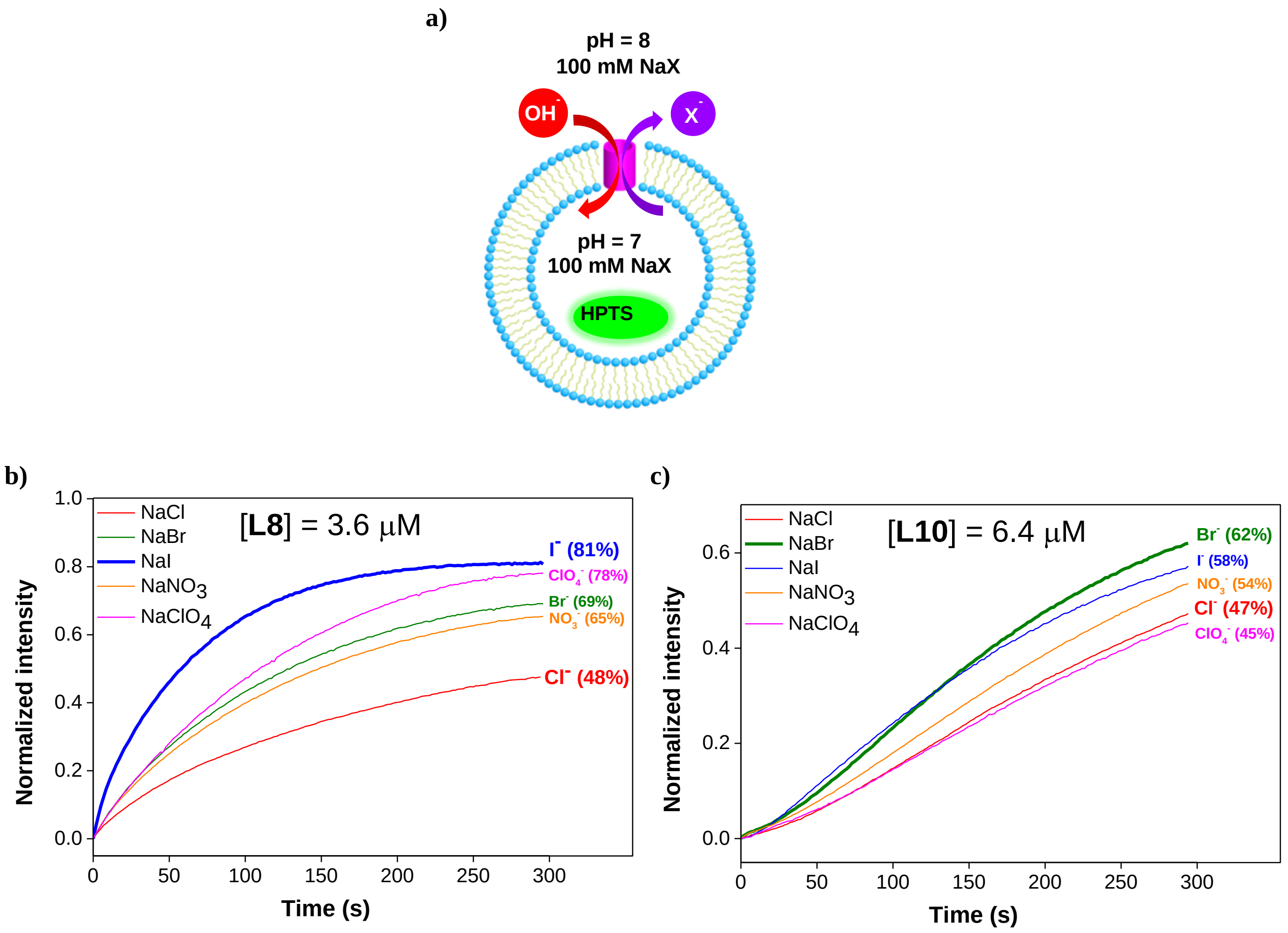


**Figure S10**. Anion transport activities using the conditions shown in a) and mediated by (b) **L8** at 3.6 μM and (c) **L10** at 6.4 μM for $Cl^-$, $Br^-$, $I^-$, $NO_3^-$ and $ClO_4^-$ and determined by the HPTS assay with both intra- and extravesicular kept as the same type of salts NaX (100 mM, $X^-$= $Cl^-$, $Br^-$, $I^-$, $NO_3^-$ and $ClO_4^-$) after background subtraction.

## Single Channel Current Measurement in Planar Lipid Bilayers

The chloroform solution of 1,2-diphytanoyl-*sn*-glycero-3-phosphocholine (diPhyPC, 10 mg/ml, 20 uL) was evaporated using nitrogen gas to form a thin film and re-dissolved in *n*-decane (8 uL). 0.2 μL of this *n*-decane solution was injected into the aperture (diameter = 200 um) of the Delrin® cup (Warner Instruments, Hamden, CT) with the *n*-decane removed using nitrogen gas. In a typical experiment for conductance measurement, both the chamber (*cis* side) and Delrin cup (*trans* side) were filled with an aqueous KCl solution (1.0 M, 1.0 mL). Ag-AgCl electrodes were inserted into the two solutions with the *cis* chamber grounded. Planar lipid bilayer was formed by painting 0.3 μL of the lipid-containing *n*-decane solution around the *n*-decane-pretreated aperture. Successful formation of planar lipid bilayers can be established with a capacitance value ranging from 80-120 pF. Samples in THF (0.3-1.0 μL) were added to the *cis* compartment to reach a final concentration of around $10^{-8}$ M and the solution was stirred for a few min until a single current trace appeared. These single channel currents were then measured using a Warner BC-535D bilayer clamp amplifier, collected by PatchMaster (HEKA) with a sample interval at 5 kHz and filtered with an 8-pole Bessel filter at 1 kHz (HEKA). The data were analysed by FitMaster (HEKA) with a digital filter at 100 Hz. Plotting current traces vs voltages yielded both potassium conduction rate ($\gamma_{K+}$).

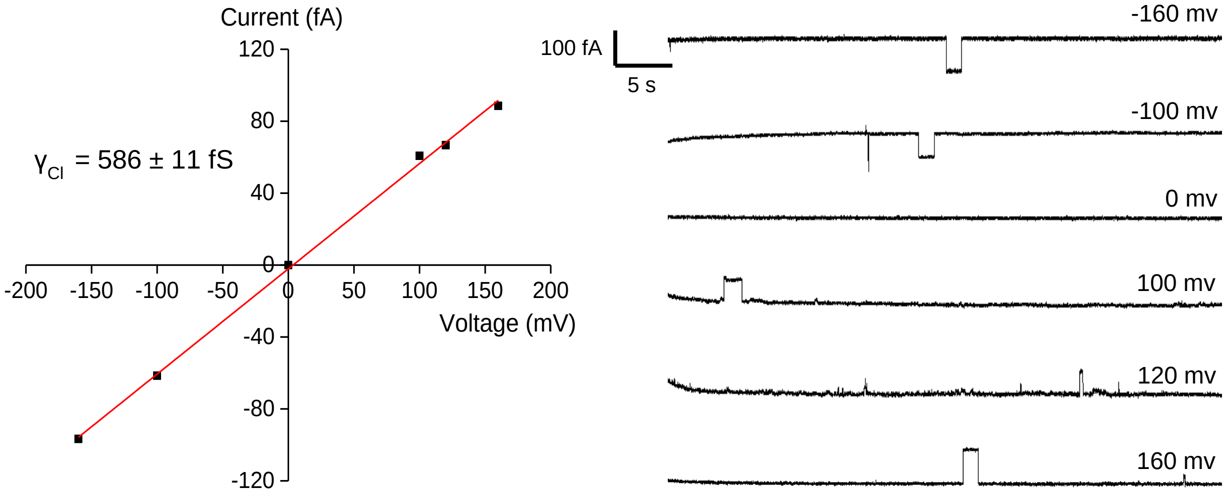


**Figure S11**. Determination of chloride conduction rate ($\gamma_{Cl^-}$) for **L8** using a linear I-V curve with the corresponding single current traces. Please note that, to roughly estimate the channel's conduction rate ($\gamma_{K+}$ = I/V), one single data point plus origin is sufficient. This is because single channel current traces are carried out in symmetrical baths with both solutions on the two sides of lipid bilayer being equal in concentration (e.g., 1 M KCl). Therefore, unless the channels are highly polarized along the channel axis, origin (e.g., 0 current at 0 voltage) is the point all I-V curves must cross. An I-V curve having five data points is used here to derive a more accurate conduction rate. The channel's open probability appears to be quite low, which is consistent with a dynamically self-assembled structure of **L8** and the fact that the well-known gramicidin channel has an opening probability of about 3%.

## Dynamic Hydrophobic Membrane Thickness of POPC Membrane

### System Setup:

Membrane builder in CHARM-GUI[1] is used to build the initial structure. The protocol comprises six steps as described by Jo et al,[2] which are sequentially performed in the following order: objects reading, objects orientation, system size determination, building of lipid bilayer, assembly of lipid bilayer, and system equilibrium. In this work, the H-bonded structure, consisting of eight molecules of **L8** (528 atoms), is placed in the center of the membrane made up of 128 molecules of 1-palmitoyl-2-oleoyl-sn-glycero-3-phosphocholine (POPC) and a total of 17152 atoms. The membrane is then placed in a box of 70Å x 70 Å in width and 74 Å in height. 4794 water molecules are placed on the top side and bottom side of the membrane (2397 each side). Counter KCl ions were added to produce an ion concentration of 0.15 M.

### MD simulation

The simulation used the CHARMM36 (C36) force filed[3] for lipids, CHARMM General Force Field (CGenFF)[3] for the repeating unit of L8 and the CHARMM TIP3P water model[4]. The periodic boundary condition (PBC) were employed and the particle mesh Ewald (PME) method[5] was used for long-range electrostatic interactions. The simulation time step was set to 2 fs in conjunction with the SHAKE algorithm[6] to constrain the covalent bonds involving hydrogen atoms. The constructed system is first relaxed through molecular mechanics (MM) minimization of 20000 steps, then heated to 303.15 K using 50 ps NPT molecular dynamics (MD) simulations, and finally equilibrated using 200 ps NPT MD simulations. During MD simulations, the pressure was maintained at 1 bar. After equilibration steps, the production run of simulation was performed for 30 ns and the last 20 ns trajectories with 1000 structures were used for analyzing.

The distributions for the concerned angles and distances were analyzed using probability density function (PDF) $f(x,\mu,\sigma)$[7]

$$f(x,\mu,\sigma)=\frac{1}{\sigma\sqrt{2\pi}}\exp\left[-\frac{(x-\mu)^2}{2\sigma^2}\right] \quad (1)$$

here $x$ is random variable. $\mu$ and $\sigma$ are mean and the standard deviation, respectively. This function describes the relative likelihood for this random variable to take on a given value, whose integral across an interval gives the probability.

(1) (a) Wu, E. L.; Cheng, X.; Jo, S.; Rui, H.; Song, K. C.; Davila-Contreras, E. M.; Qi, Y.; Lee, J.; Monje-Galvan, V.; Venable, R. M.; Klauda, J. B.; Im, W. *J. Comput. Chem.* **2014**, *35*, 1997; (b) Jo, S.; Kim, T.; Iyer, V. G.; Im, W. *J. Comput. Chem.* **2008**, *29*, 1859.

(2) Jo, S.; Lim, J. B.; Klauda, J. B.; Im, W. *Biophysical journal* **2009**, *97*, 50.

(3) Vanommeslaeghe, K.; Hatcher, E.; Acharya, C.; Kundu, S.; Zhong, S.; Shim, J.; Darian, E.; Guvench, O.; Lopes, P.; Vorobyov, I.; MacKerell, A. D. *Journal of Computational Chemistry* **2010**, *31*, 671.

(4) Jorgensen, W. L.; Chandrasekhar, J.; Madura, J. D.; Impey, R. W.; Klein, M. L. *J Chem Phys* **1983**, *79*, 926.

(5) Essmann, U.; Perera, L.; Berkowitz, M. L.; Darden, T.; Lee, H.; Pedersen, L. G. *J Chem Phys* **1995**, *103*, 8577.

(6) Martyna, G. J.; Tobias, D. J.; Klein, M. L. *J Chem Phys* **1994**, *101*, 4177.

(7) Bopege, D. N.; Petrowsky, M.; Fleshman, A. M.; Frech, R.; Johnson, M. B. *J Phys Chem B* **2012**, *116*, 71.

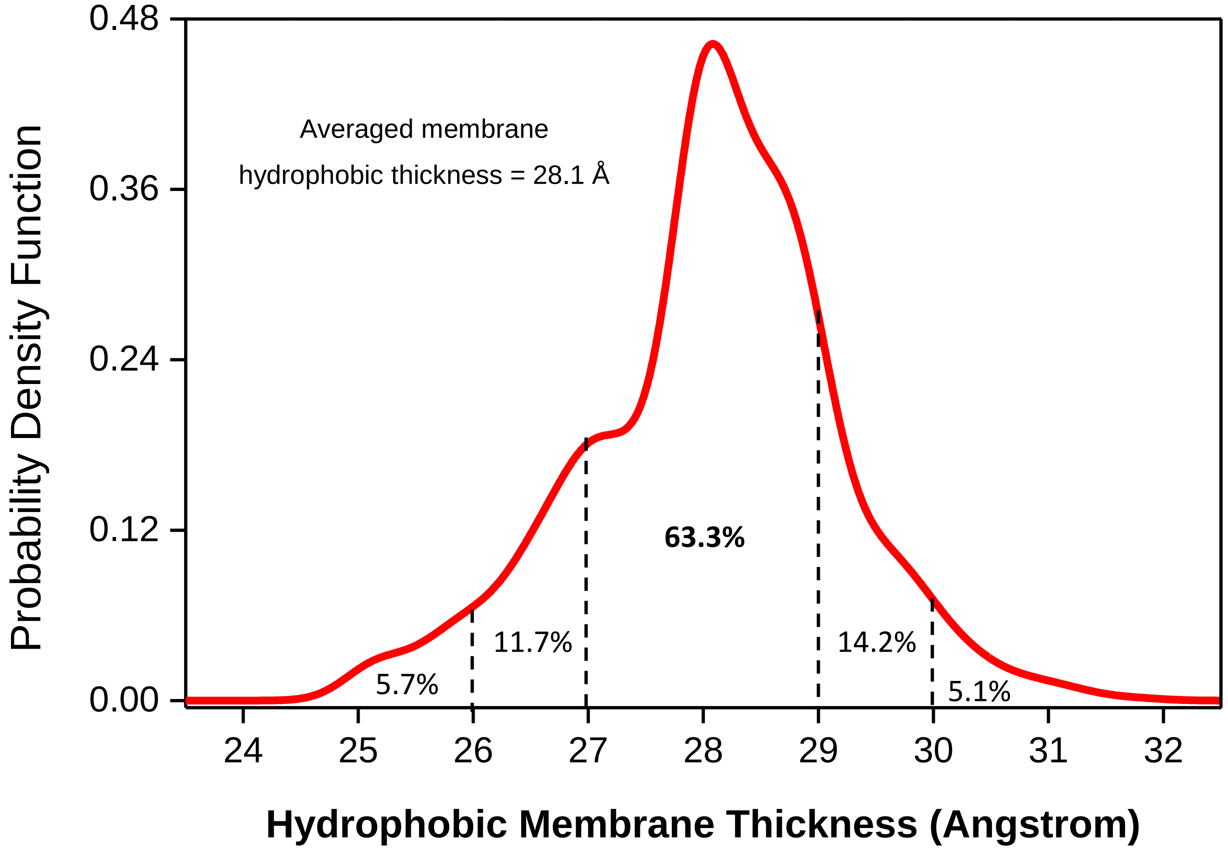


**Figure S12.** Dynamic distribution of hydrophobic membrane thickness for POPC lipids. This graph was obtained after analyzing 1000 structural snapshots. In other words, 63.3% means that there are 633 structures having a thickness falling with 27-29 Å.

**$EC_{50}$ Values for A10, L8 and L10 using Cholesterol-Free LUVs (Matile's Condition, see *J. Am. Chem. Soc.* 2013, *135*, 5302)**

Egg yolk L-α-phosphatidylcholine (EYPC, 1 ml, 25 mg/mL in $CHCl_3$, Avanti Polar Lipids, USA) and MeOH (1 mL) were mixed in a round-bottom flask. The mixed solvents were removed under reduced pressure at 40 $^{o}$C. After drying the resulting film under high vacuum overnight at room temperature, the film was hydrated with 4-(2-hydroxyethyl)-1-piperazine-ethane sulfonic acid (HEPES) buffer solution (1 mL, 10 mM HEPES, 100 mM NaCl, pH = 7.0) containing a pH sensitive dye 8-hydrox-ypyrene-1,3,6-trisulfonic acid (HPTS, 1 mM) at room temperature for 60 minutes to give a milky suspension. The mixture was then subjected to 12 freeze-thaw cycles: freezing in liquid $N_2$ for 1 minute and heating at 37 $^{o}$C in water bath for 1.5 minutes. The vesicle suspension was extruded through polycarbonate membrane (0.1 μm) to produce a homogeneous suspension of large unilamellar vesicles (LUVs) of about 120 nm in diameter with HPTS encapsulated inside. The unencapsulated HPTS dye was separated from the LUVs by using size exclusion chromatography (stationary phase: Sephadex G-50, GE Healthcare, USA, mobile phase: HEPES buffer with 100 mM NaCl) and diluted with the mobile phase to yield 12.8 mL of 2.5 mM lipid stock solution.

The HPTS-containing LUV suspension (25 μL, 2.5 mM in 10 mM HEPES buffer containing 100 mM NaCl at pH = 7.0) was added to a HEPES buffer solution (1.93 mL, 10 mM HEPES, 100 mM NaCl at pH = 8.0) to create a pH gradient for ion transport study. A solution of channel molecules in DMSO was then injected into the suspension under gentle stirring. Upon the addition of channel molecules, the emission of HPTS was immediately monitored at 510 nm with excitations at both 460 and 403 nm recorded simultaneously for 300 seconds using fluorescence spectrophotometer (Hitachi, Model F-7100, Japan) after which time an aqueous solution of Triton X-100 (30 μL, 20% v/v) was immediately added to achieve the maximum change in fluorescence dye emission. The final transport trace was obtained as a ratiometric value of $I_{460}/I_{403}$ and normalized based on the aforementioned equation S1.

The fractional changes $R$ was calculated for each curve using the normalized value of $I_{460}/I_{403}$ at 300 seconds before the addition of triton, referring to the ratio of blank as 0 and that of triton as 1. Fitting the fractional transmembrane activity $R$ vs channel concentration using the Hill equation: $Y=1/(1+ (EC_{50}/[C])^{n})$ gave the Hill coefficient $n$ and $EC_{50}$ values.

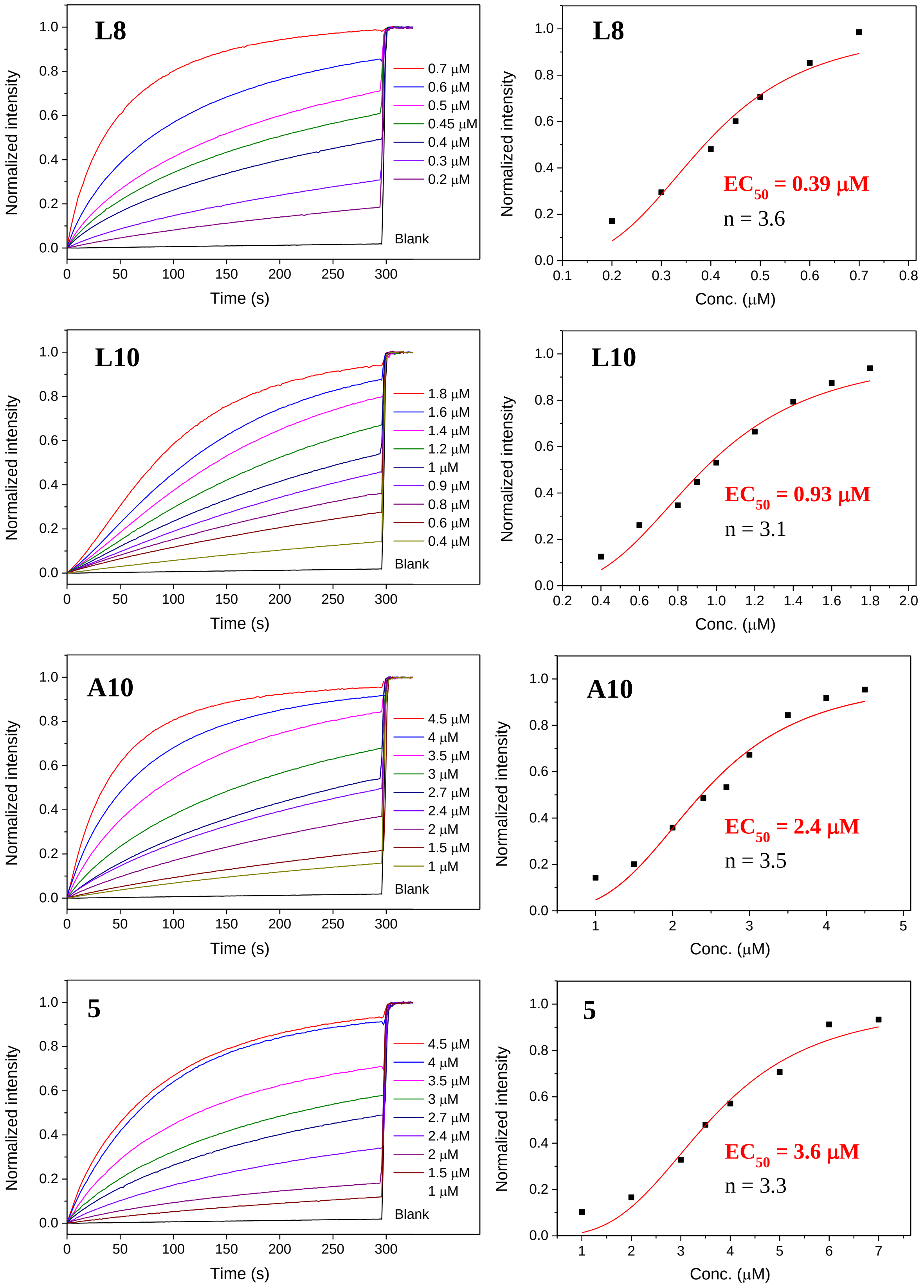


**Figure S13**. Determination of $EC_{50}$ values for chloride transport using the ratiometric values of I460/I403 at different concentrations as a function of time for L8, L10, A10 and 5 under matile's Condition (*J. Am. Chem. Soc.* 2013, *135*, 5302).

### $EC_{50}$ Determination for 5 using Cholesterol-Containing LUVs

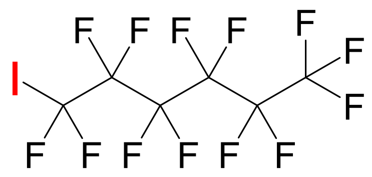


**5**

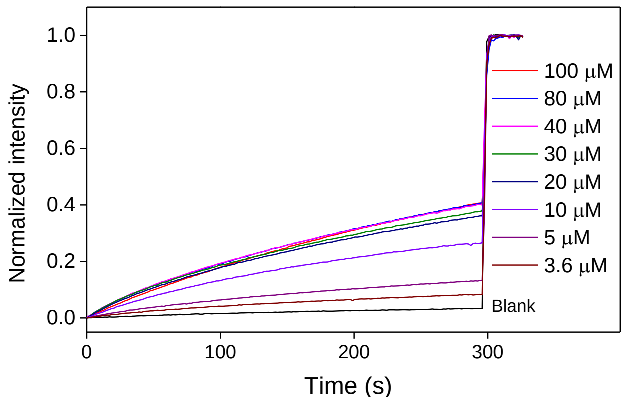


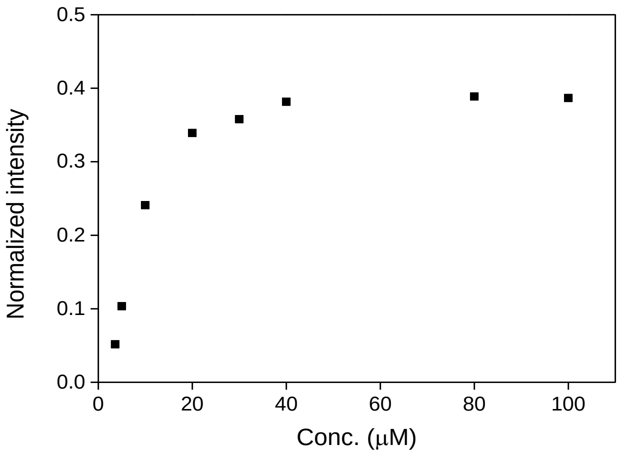


**Figure S14**. Under our assay conditions using cholesterol-containing LUVs, carrier **5**, which is highly active in the absence of cholesterol, exhibits a quite moderate activity of 38% across 40 - 100 μM. From these curves, $EC_{50}$ value can't be determined, but can be estimated to be much larger than 40 μM.

## Determination of Cancer Cell Viability via MTT Assay

The mitochondria toxicity of the channels was examined by MTT ((3-(4,5-dimethylthiazol-2-yl)-2,5-diphenyl tetrazolium) assay using human breast cancer cell lines BT-474 (ATCC). The BT-474 cells were cultured in Dulbecco's Modified Eagle Medium (DMEM, Gibco), which was supplemented with 10% fetal bovine serum (FBS, Invitrogen) and 1% penicillin/streptomycin (Gibco) at 37 ˚C with 5% $CO_2$. Upon reaching ~80-90% cell confluency, the cells were seeded in 96-well plates with cell density of $1 \times 10^4$ cells/well in 100 µL of DMEM, and incubated at 37 ˚C with 5% $CO_2$ for 24 or 72 h. The DMEM was then removed and the chloride-transporting channels of various concentrations (0-200 µM, 100 µL) in DMEM were added in the cell-containing 96-well plates. After incubation for 24 or 72 h, the medium containing peptides were removed and replaced with 100 µL of fresh DMEM and 20 µL of MTT solution at 5 mg/mL in PBS. After incubation for 4 h, the DMEM and MTT were removed and 150 µL of DMSO were added. The plates were shaken for 5 min to thoroughly dissolve the purple formazan crystals. The absorbance of the 96-well plates recorded at 570 nm were used to calculate the cell viability using the following formula: Viability (%) = [(O.D.$_{570nm}$ of the treated cells – O.D.$_{570nm}$ of the blank well without cells)/(O.D.$_{570nm}$ of untreated cells – O.D.$_{570nm}$ of blank well without cells)] $\times$ 100.

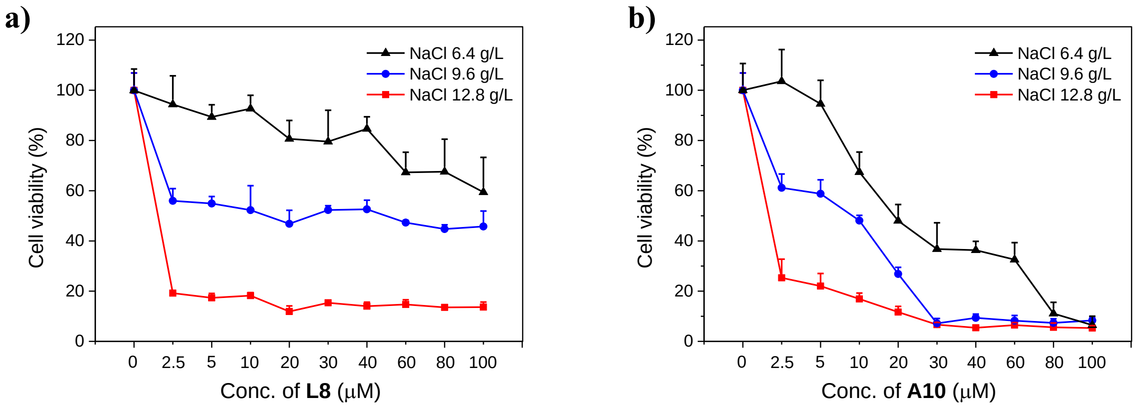


**Figure S15.** Cell viabilities of breast cancer cells BT-474 cultured in media containing different concentrations of NaCl and **L8** or **A10** after 72 h, respectively.

## $^{1}H$ NMR and $^{13}C$ NMR

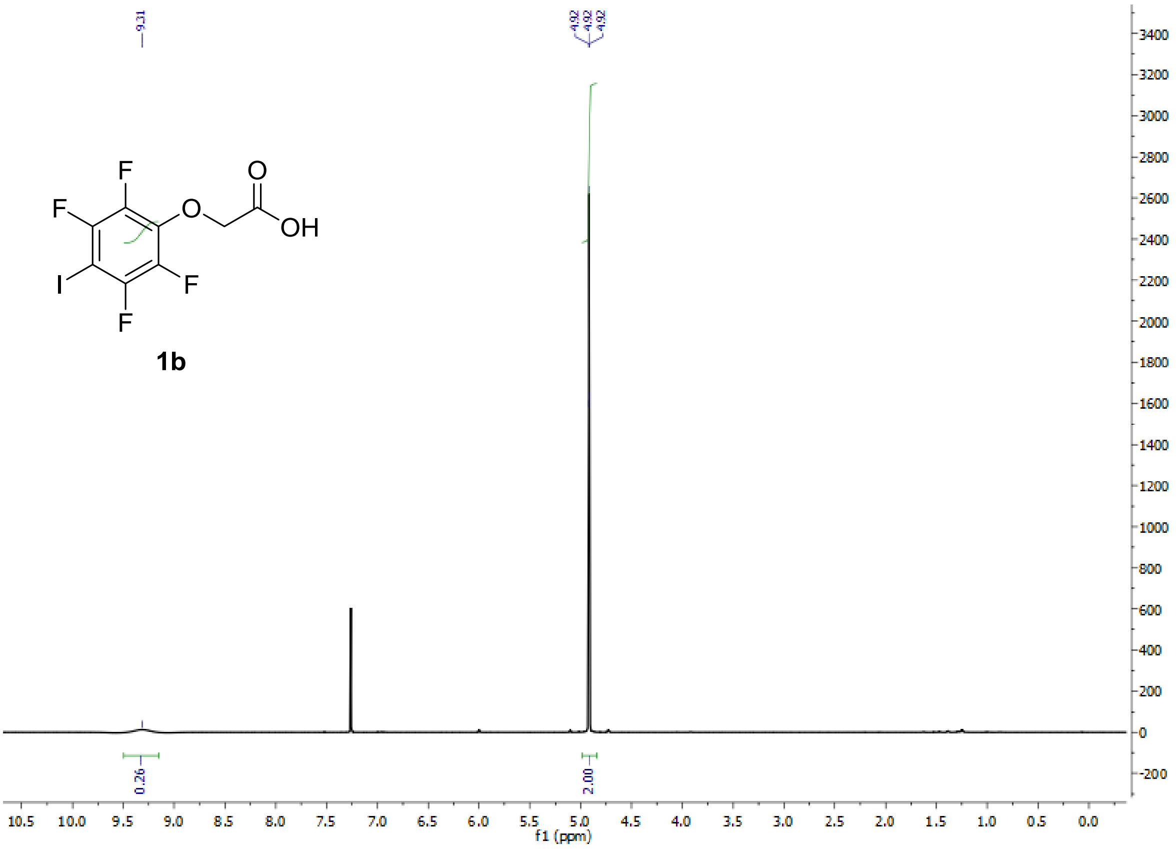


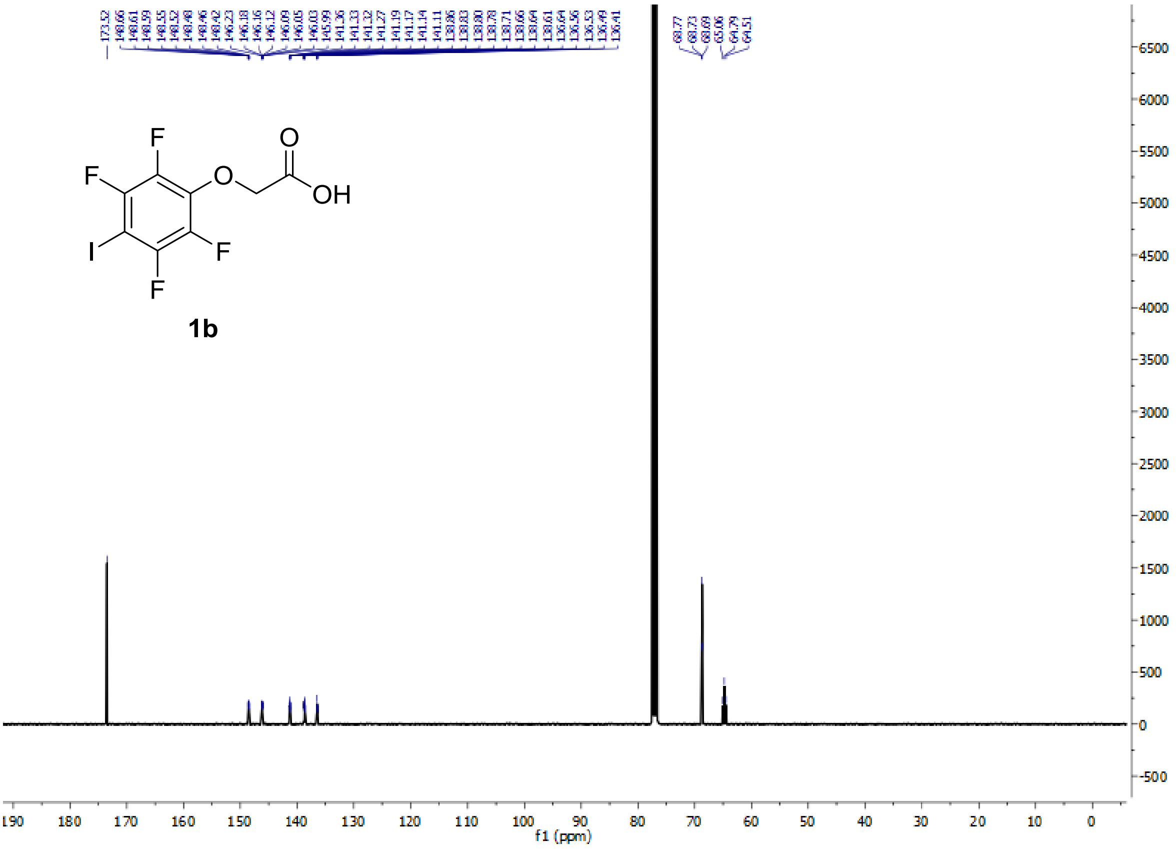

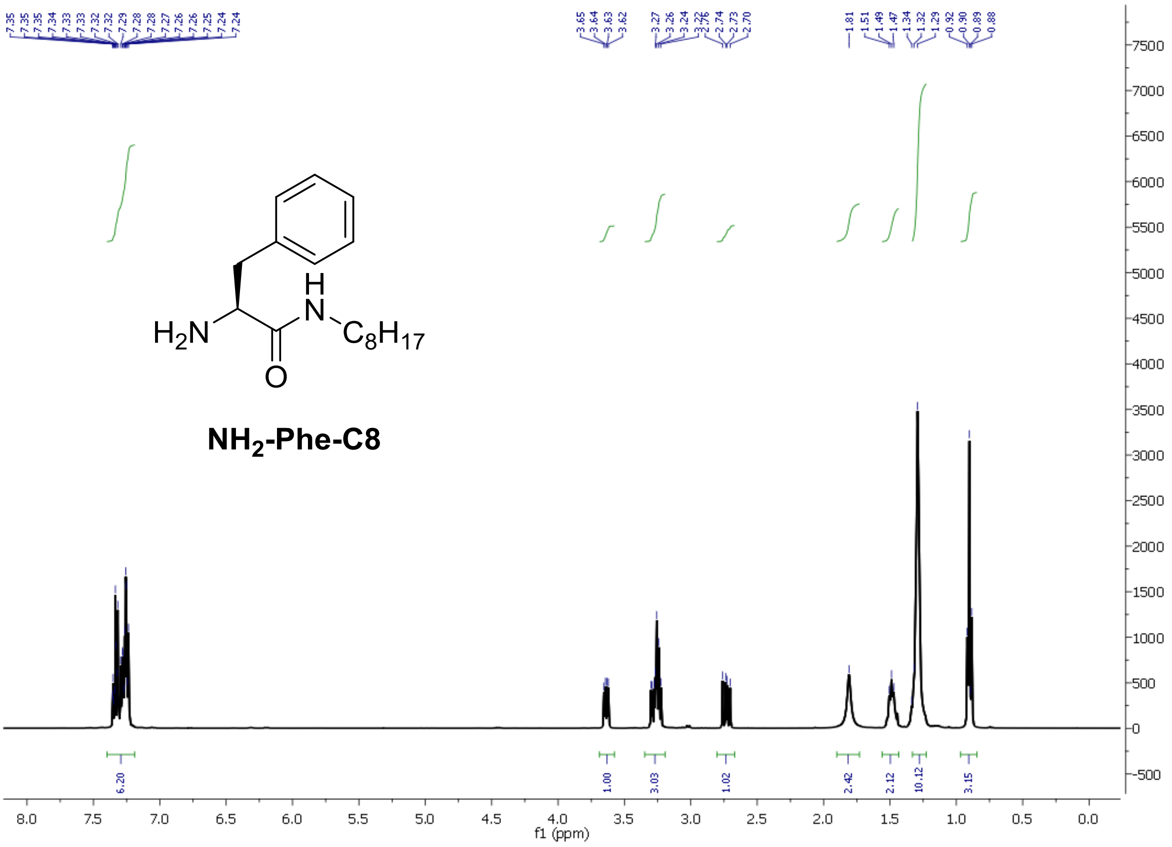
NH2-Phe-C8
f1 (ppm)

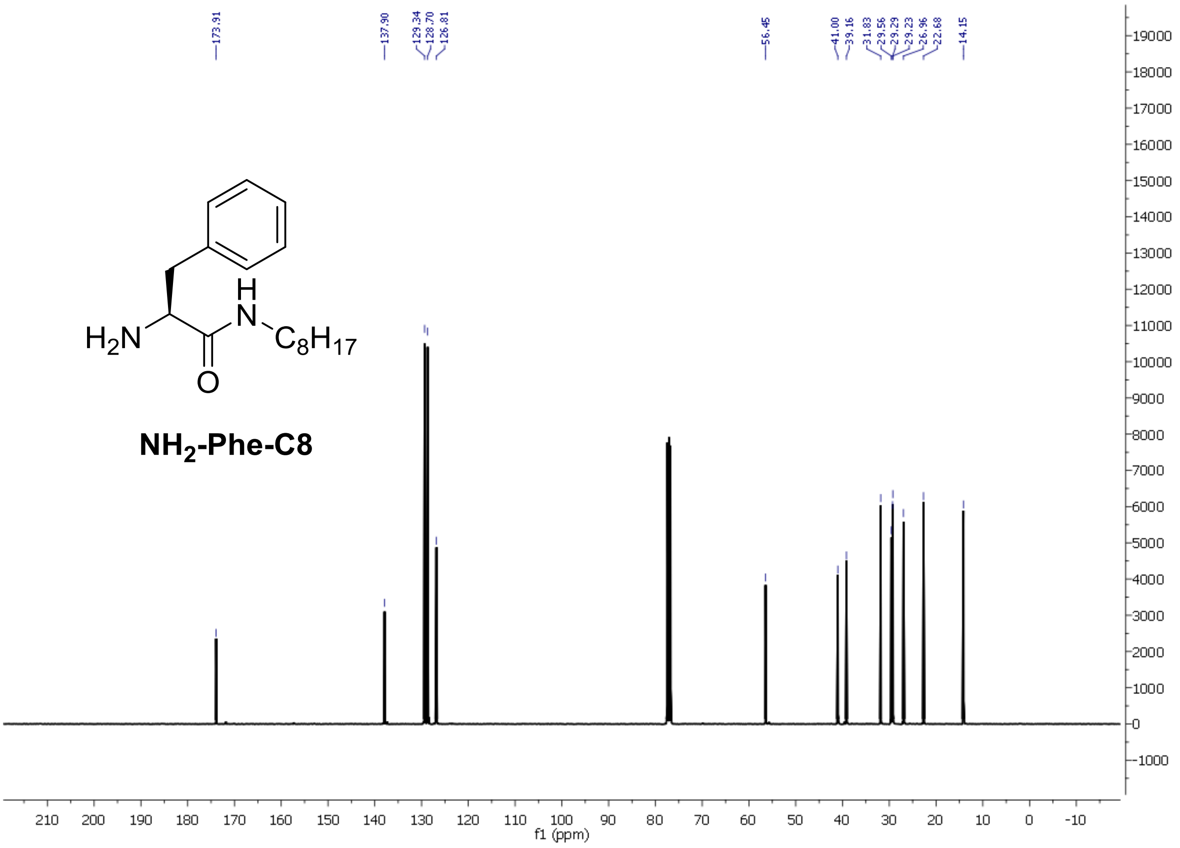
NH2-Phe-C8
f1 (ppm)

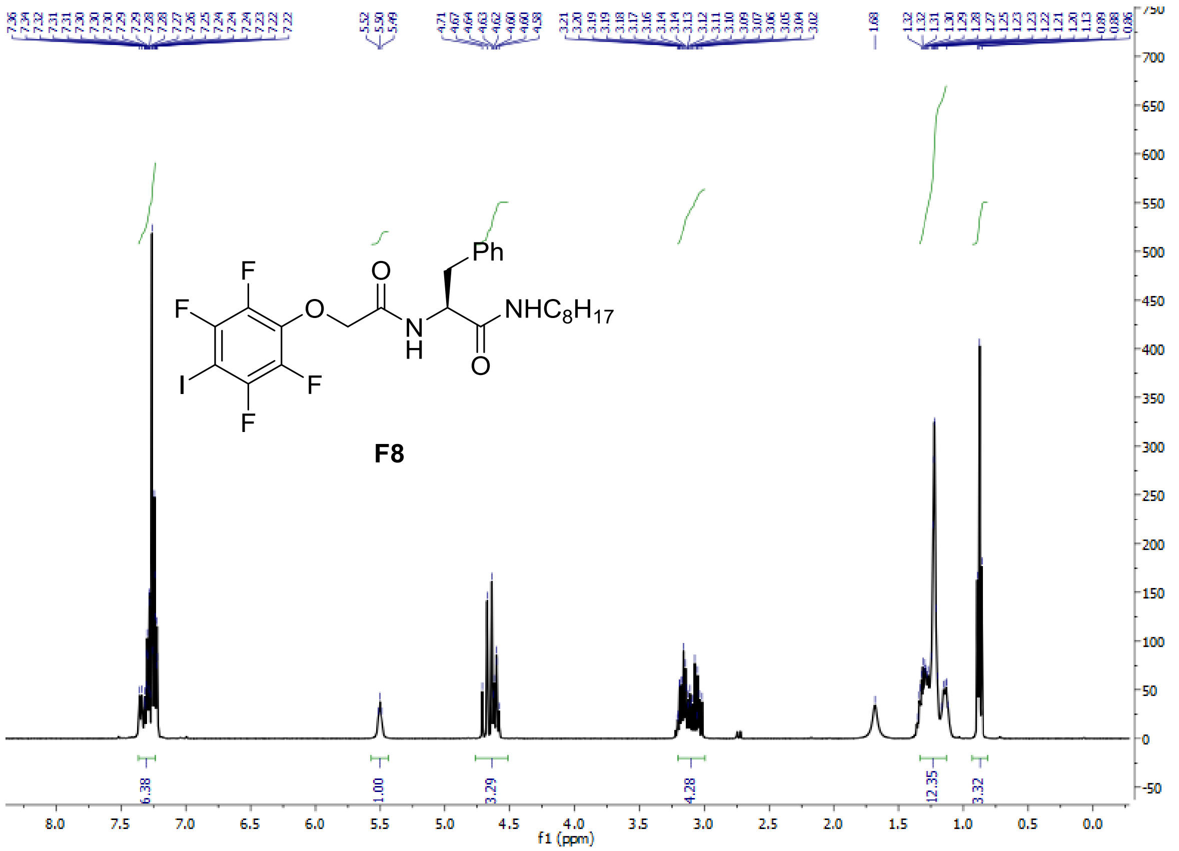
7.36 7.34 7.32 7.31 7.31 7.30 7.30 7.30 7.29 7.29 7.28 7.28 7.27 7.26 7.25 7.24 7.24 7.24 7.23 7.22 7.22
5.52 5.50 5.49
4.71 4.67 4.64 4.63 4.62 4.62 4.60 4.60 4.58
3.21 3.20 3.19 3.19 3.18 3.17 3.16 3.14 3.14 3.13 3.12 3.11 3.10 3.09 3.07 3.06 3.05 3.04 3.02
1.68
1.32 1.32 1.31 1.30 1.29 1.28 1.27 1.25 1.23 1.23 1.22 1.21 1.20 1.13 0.89 0.88 0.86
F
F
O
O
Ph
N
H
NHC8H17
I
F
F
O
F8
6.38
1.00
3.29
4.28
12.35
3.32
f1 (ppm)

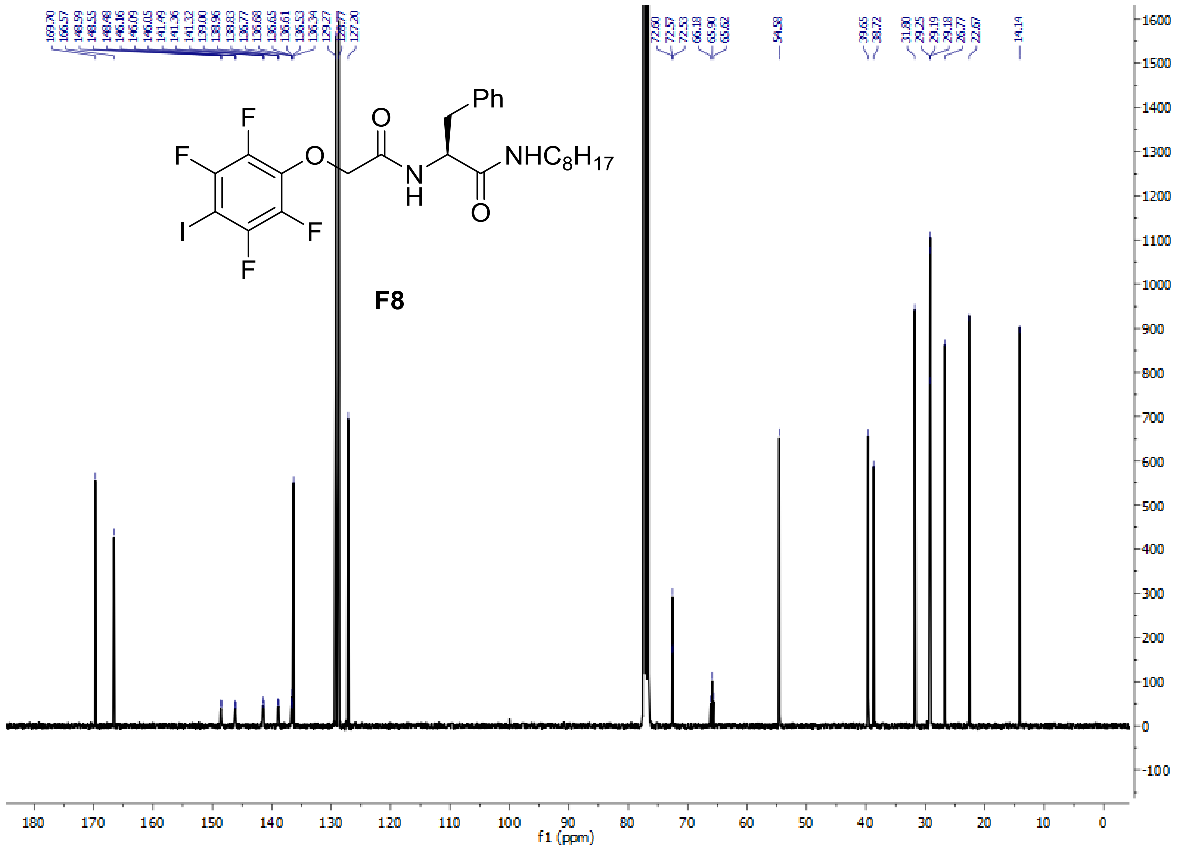
169.70 166.57 148.59 148.55 148.48 146.16 146.09 146.05 141.49 141.36 141.32 139.00 138.96 138.83 136.77 136.68 136.65 136.61 136.53 136.34 129.27 128.77 127.20
72.60 72.57 72.53 66.18 65.90 65.62
54.58
39.65 38.72
31.80 29.25 29.19 29.18 26.77 22.67
14.14
F
F
O
O
Ph
N
H
NHC8H17
I
F
F
O
F8
f1 (ppm)

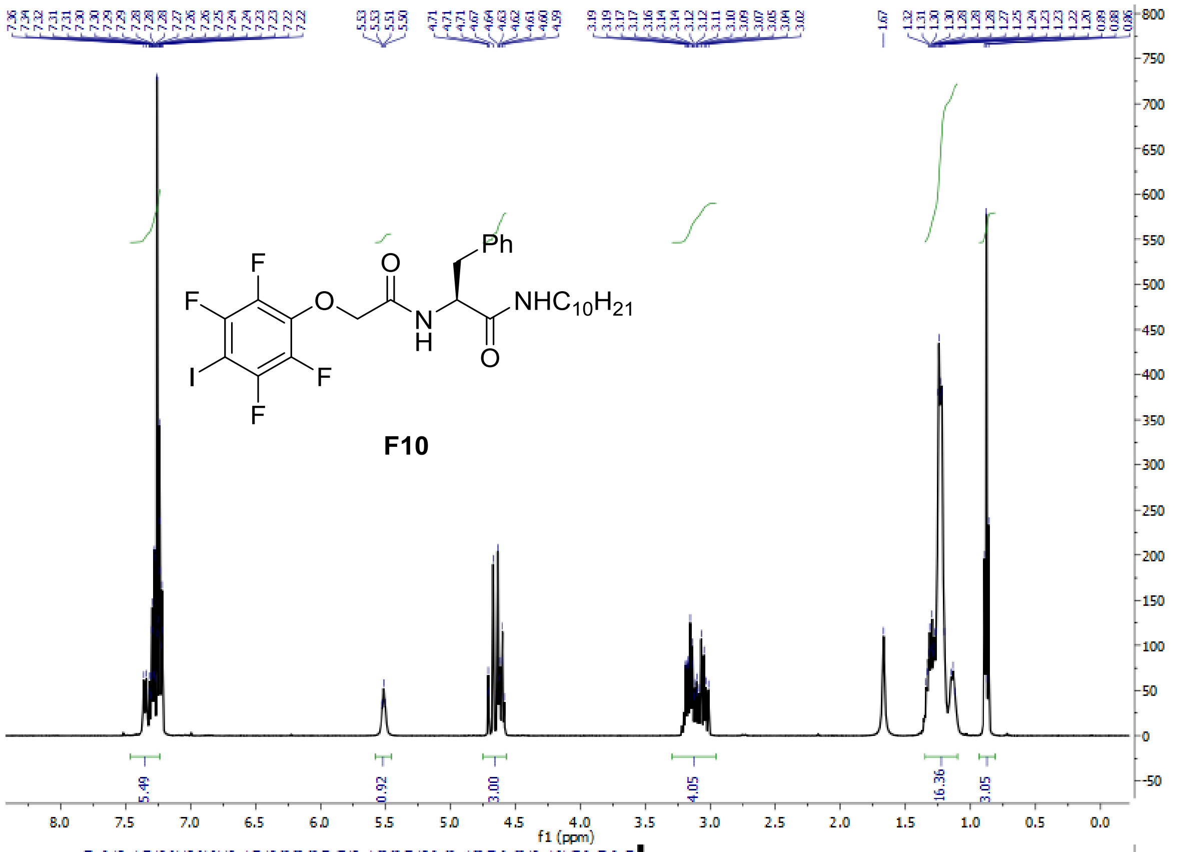

F10
f1 (ppm)


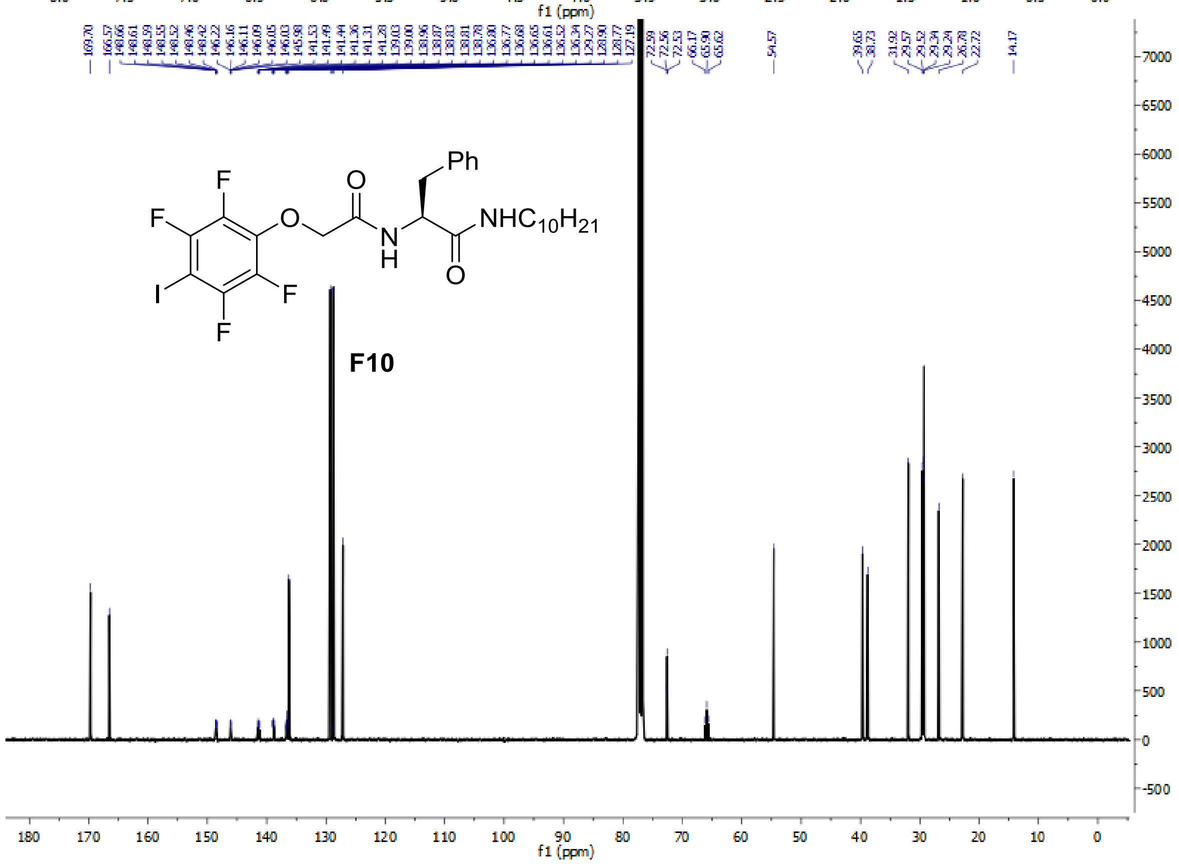

F10
f1 (ppm)

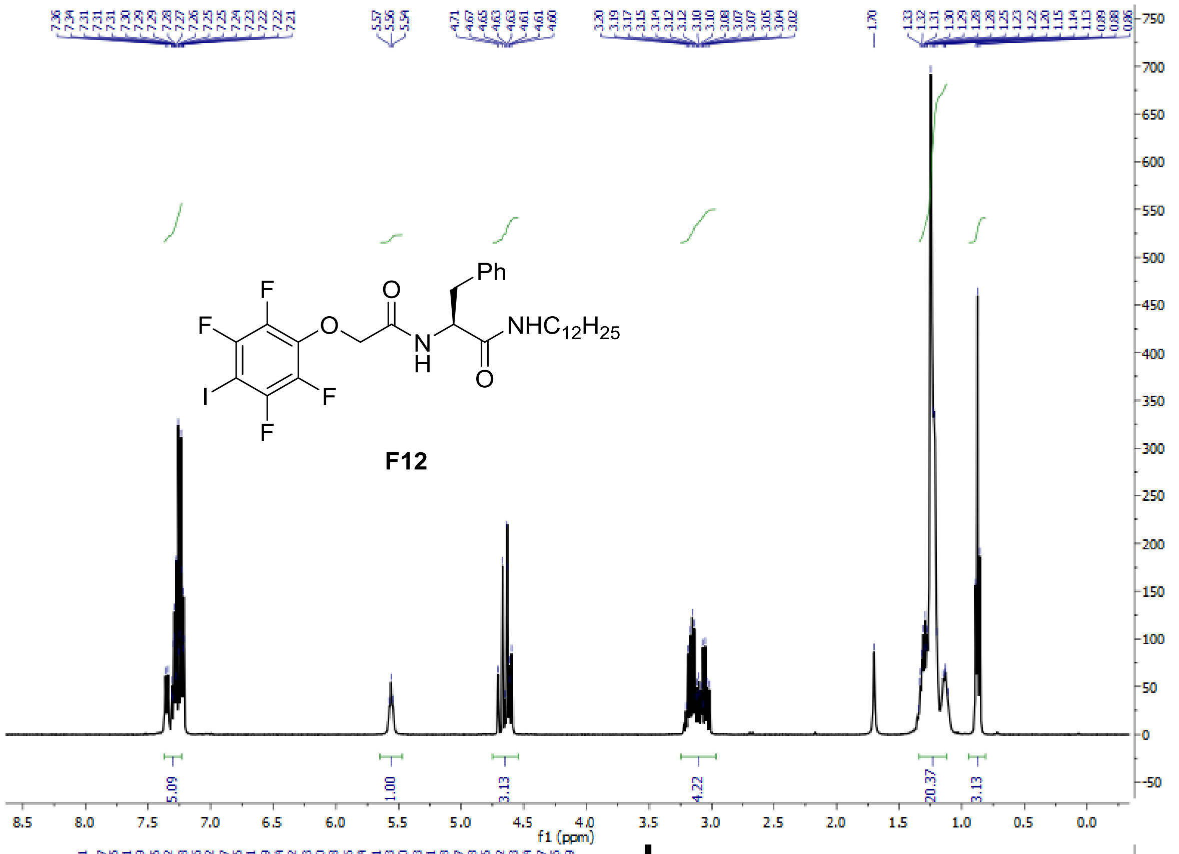
F12

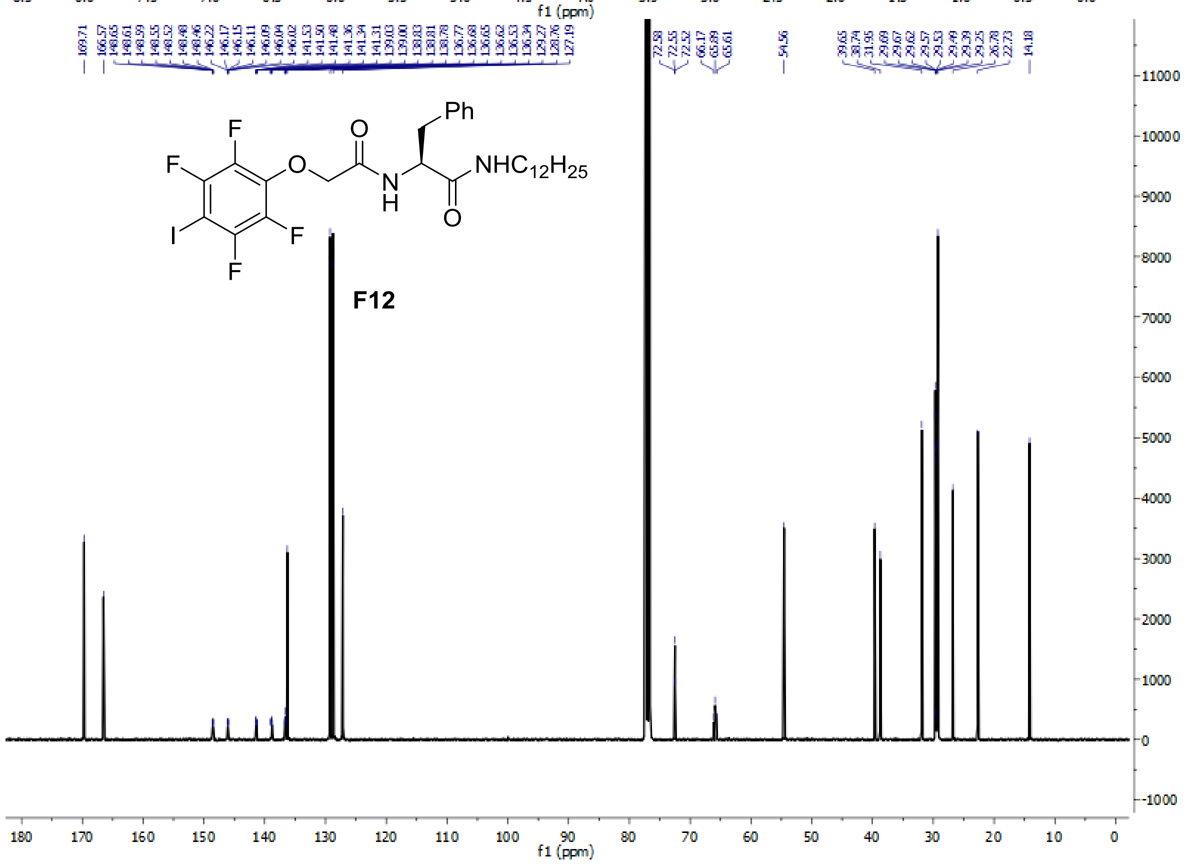
F12

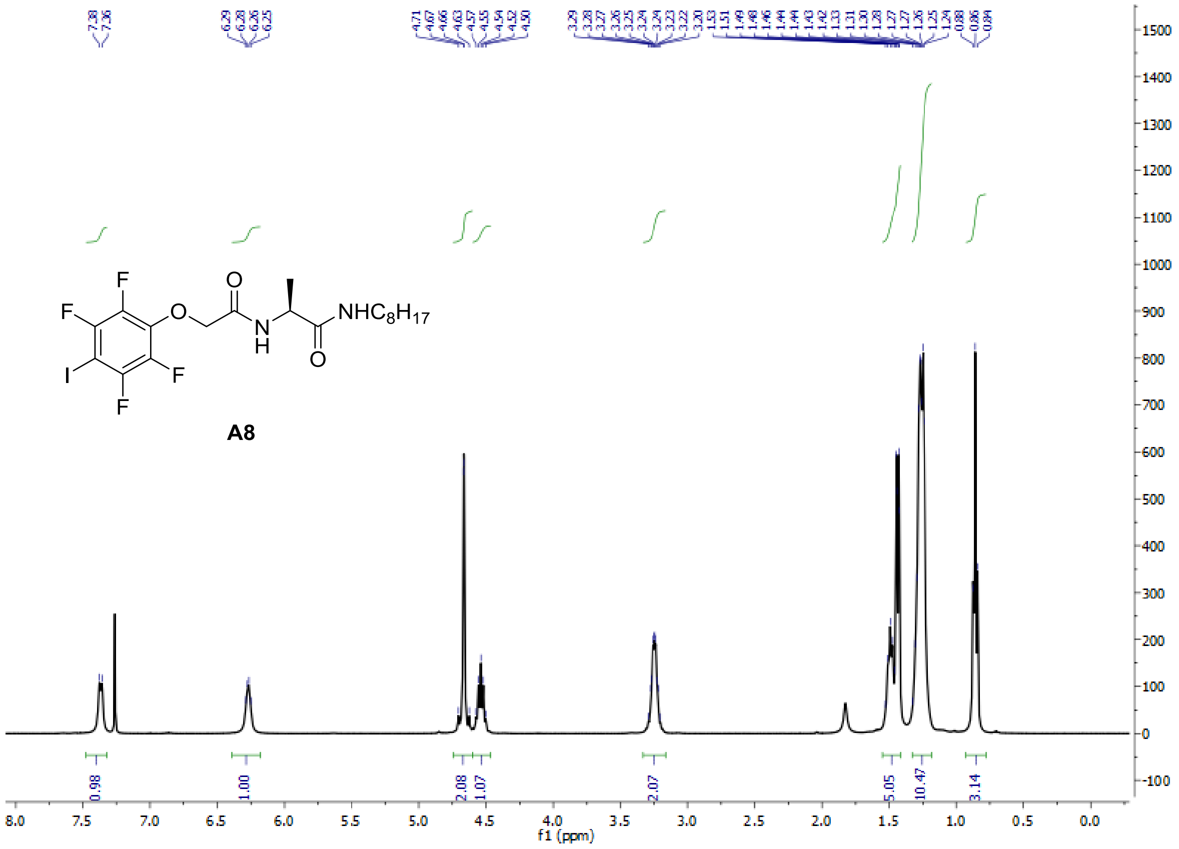

A8
f1 (ppm)
0.98
1.00
2.08
1.07
2.07
5.05
10.47
3.14


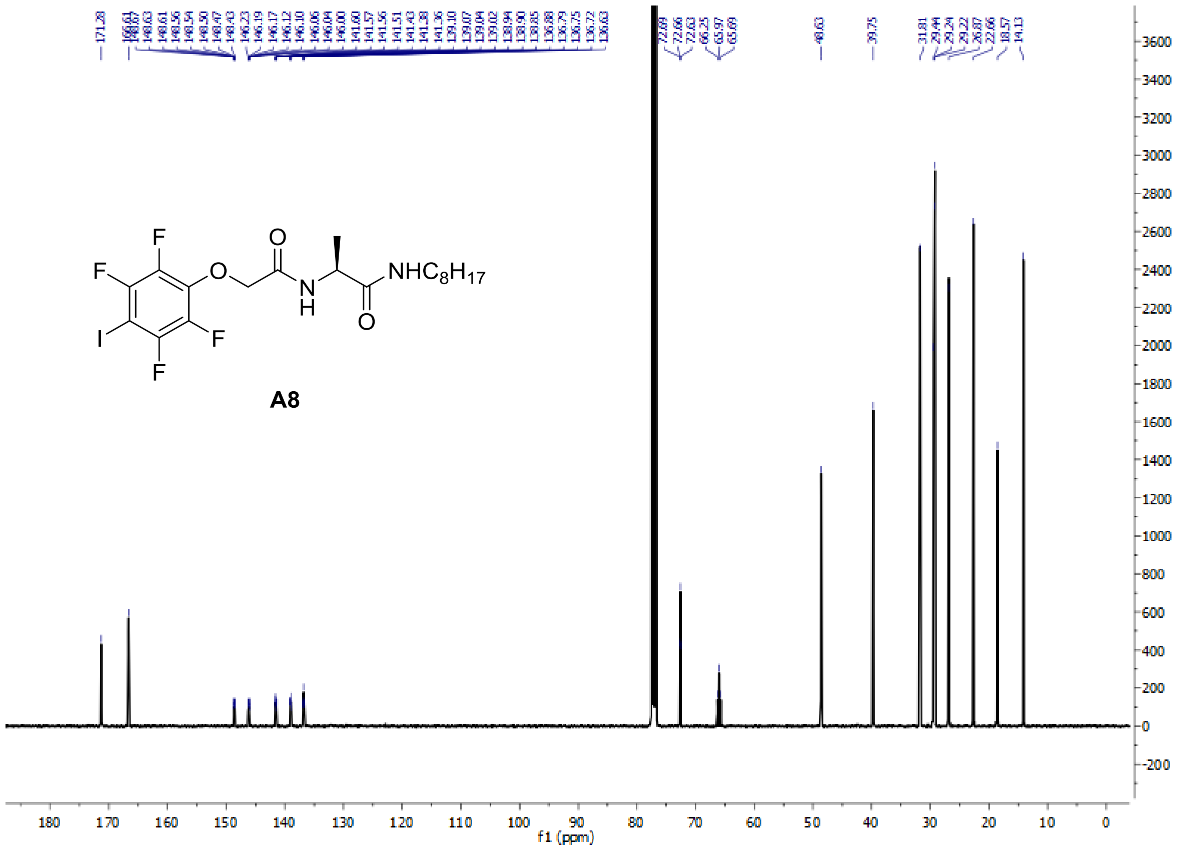

A8
f1 (ppm)
171.28
166.61
72.69
72.66
72.63
66.25
65.97
65.69
48.63
39.75
31.81
29.44
29.24
29.22
26.87
22.66
18.57
14.13

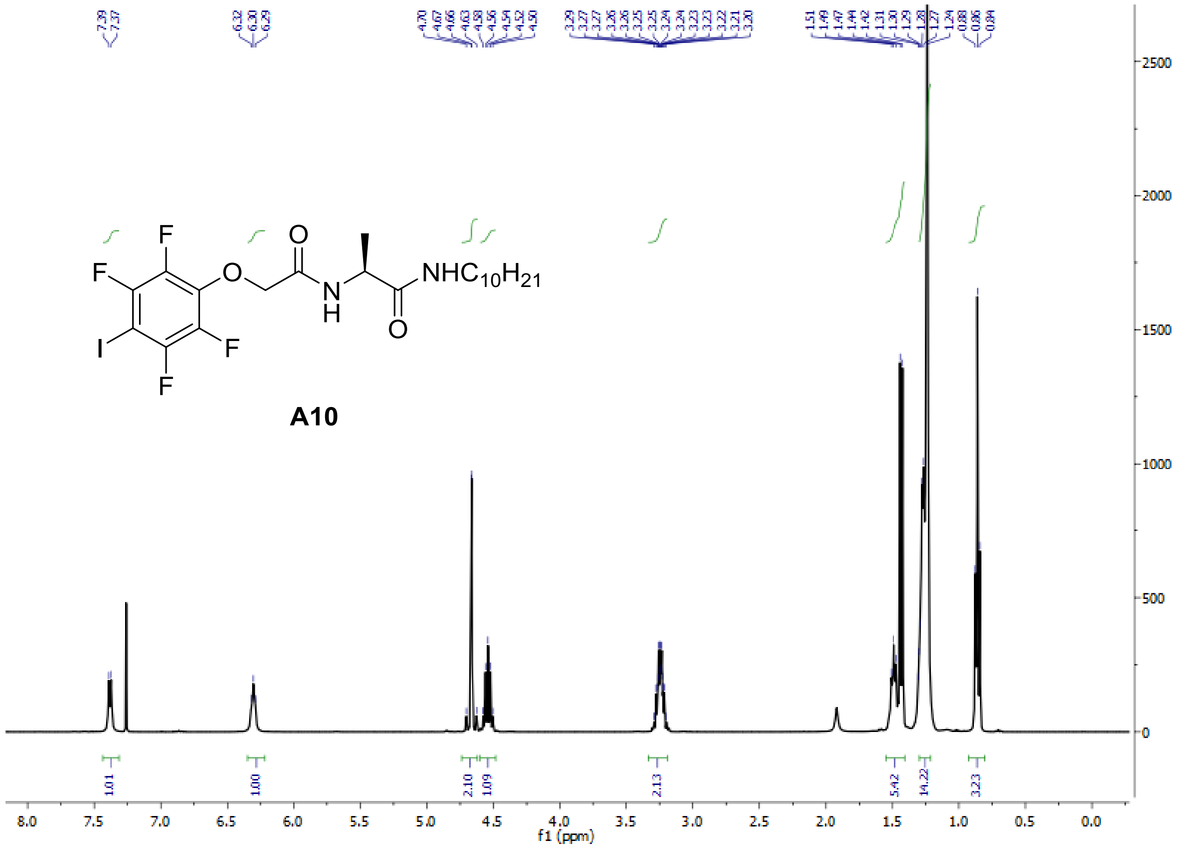
7.39
7.37
6.32
6.30
6.29
4.70
4.67
4.66
4.63
4.58
4.56
4.54
4.52
4.50
3.29
3.27
3.27
3.26
3.26
3.25
3.25
3.24
3.24
3.23
3.23
3.22
3.21
3.20
1.51
1.49
1.47
1.44
1.42
1.31
1.30
1.29
1.28
1.27
1.24
0.88
0.86
0.84
F
F
O
O
N
H
NHC10H21
I
F
F
O
A10
1.01
1.00
2.10
1.09
2.13
5.42
14.22
3.23
f1 (ppm)

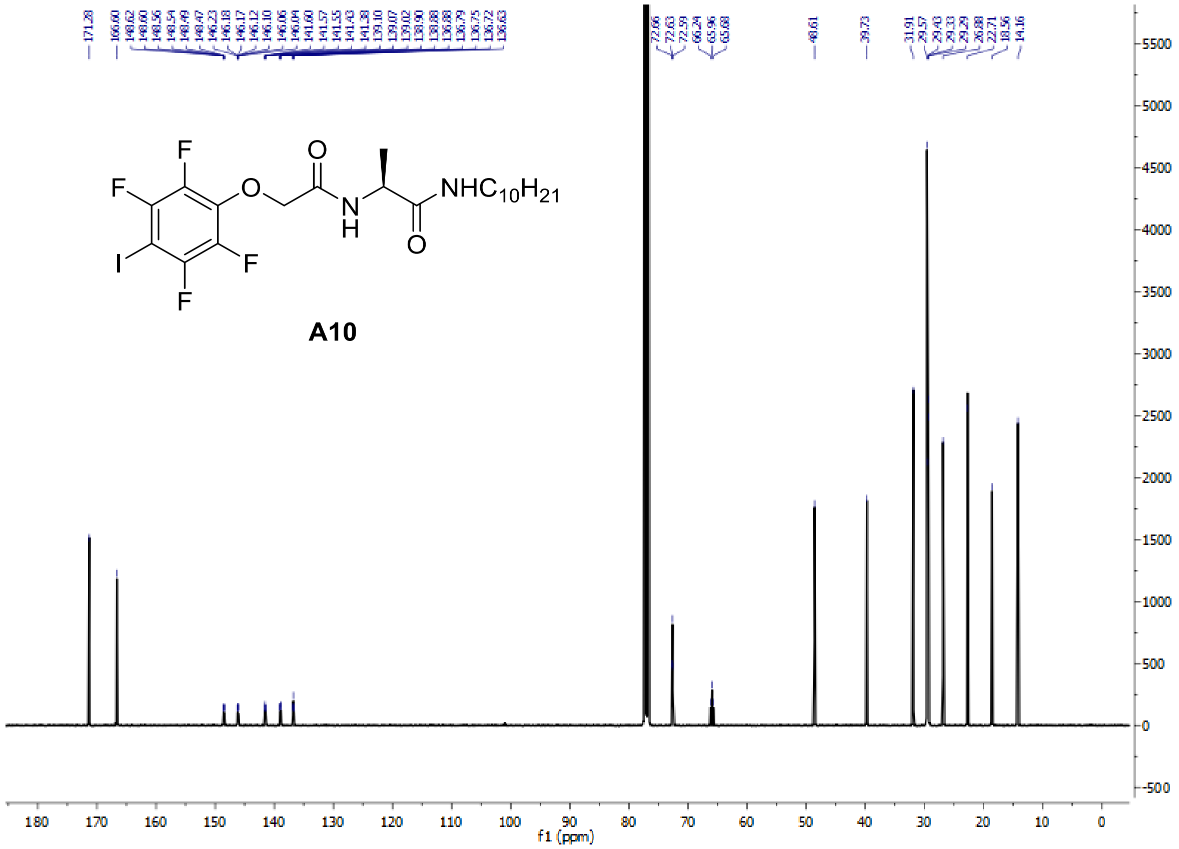
171.28
166.60
148.62
148.60
148.56
148.54
148.49
148.47
146.23
146.18
146.17
146.12
146.10
146.06
146.04
141.60
141.57
141.55
141.43
141.38
139.10
139.07
139.02
138.90
138.88
136.79
136.75
136.72
136.63
72.66
72.63
72.59
66.24
65.96
65.68
48.61
39.73
31.91
29.57
29.43
29.33
29.29
26.88
22.71
18.56
14.16
F
F
O
O
N
H
NHC10H21
I
F
F
O
A10
f1 (ppm)

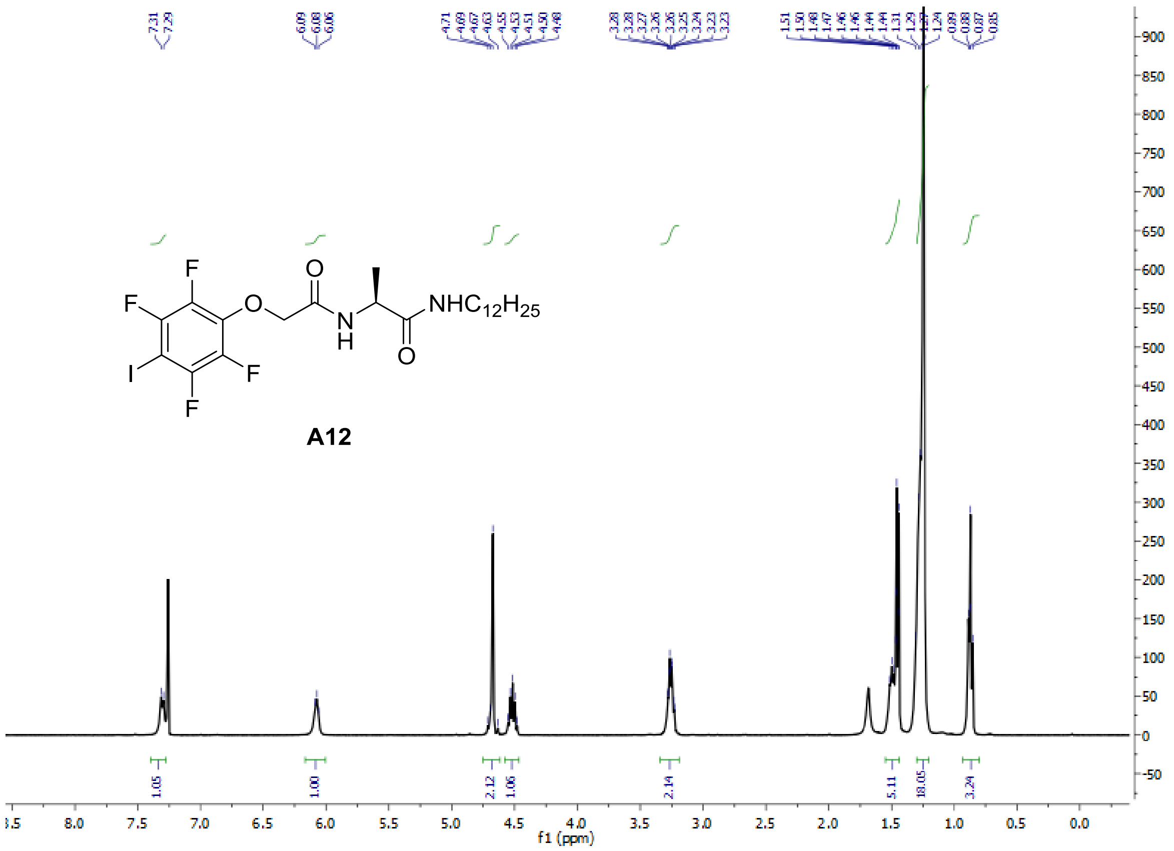
NHC12H25
A12
f1 (ppm)

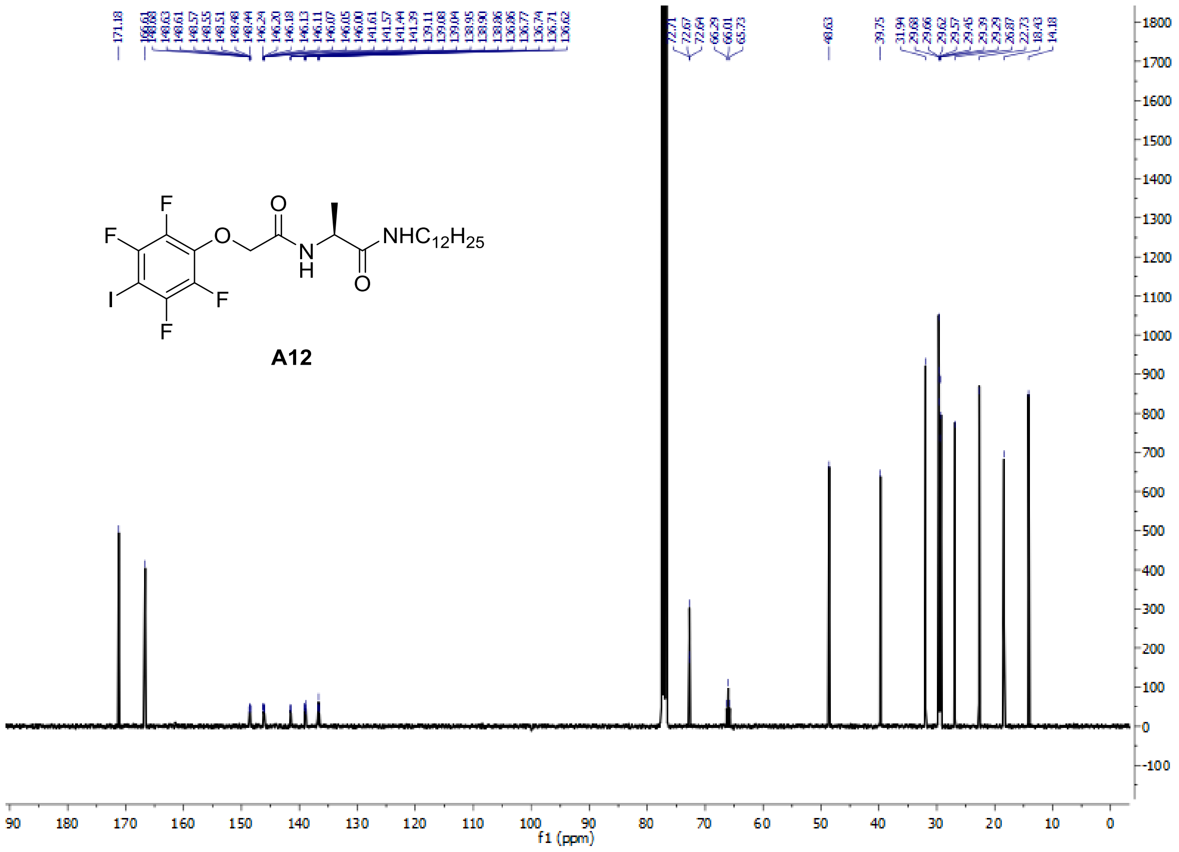
NHC12H25
A12
f1 (ppm)

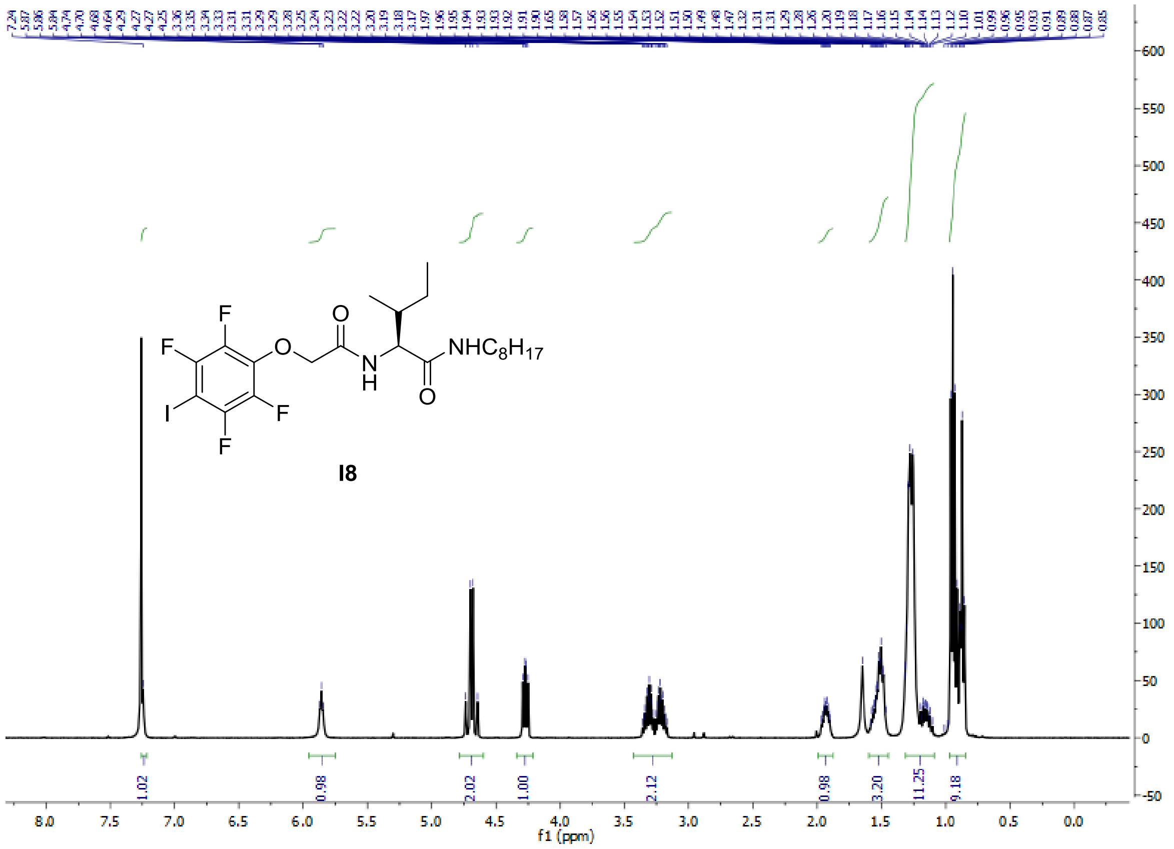
I8
f1 (ppm)

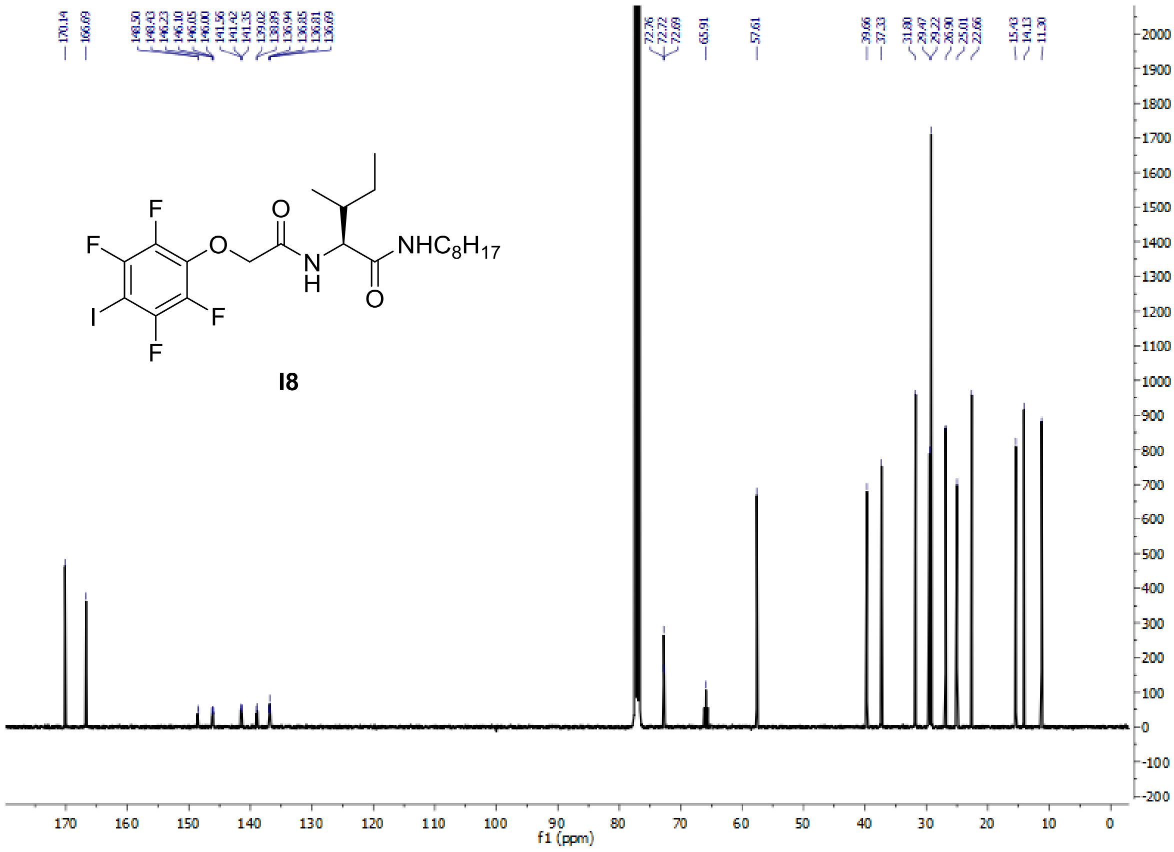
I8
f1 (ppm)

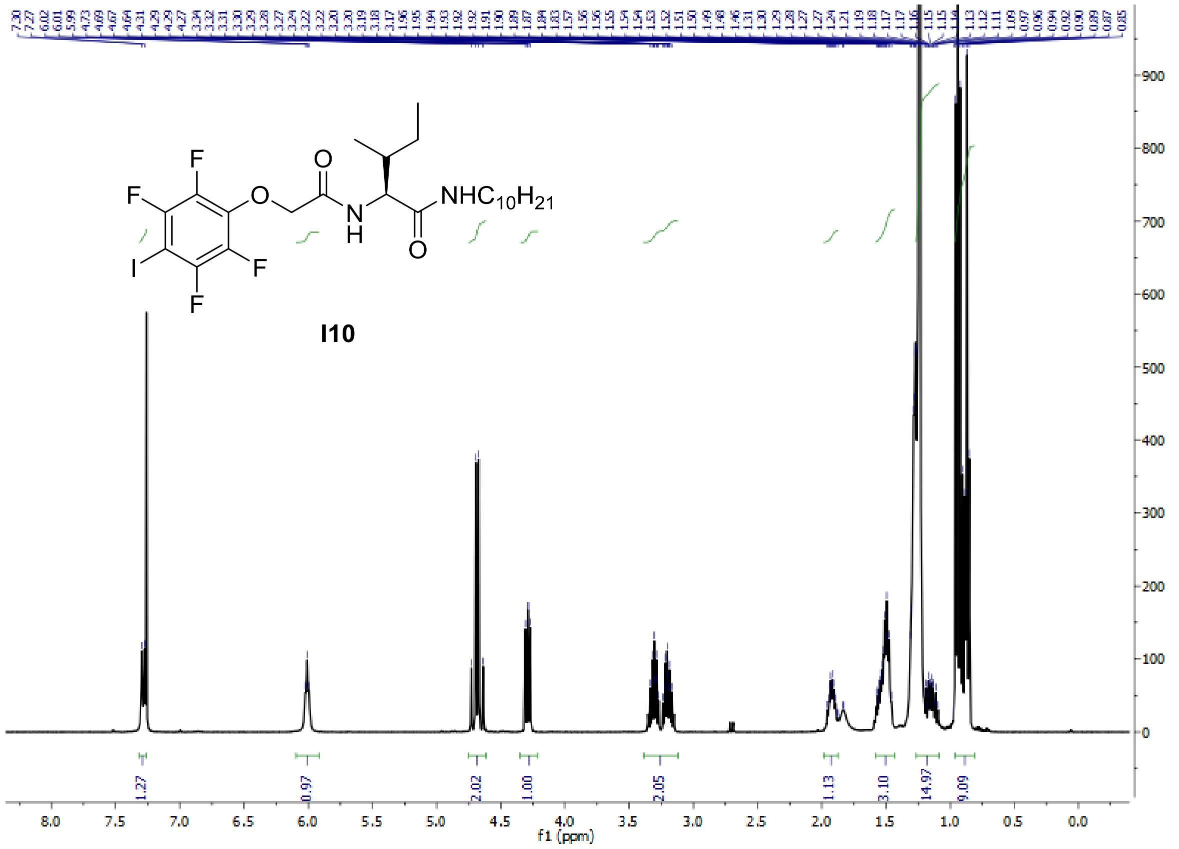
F
F
I
F
F
O
O
N
H
NHC10H21
O
I10
f1 (ppm)

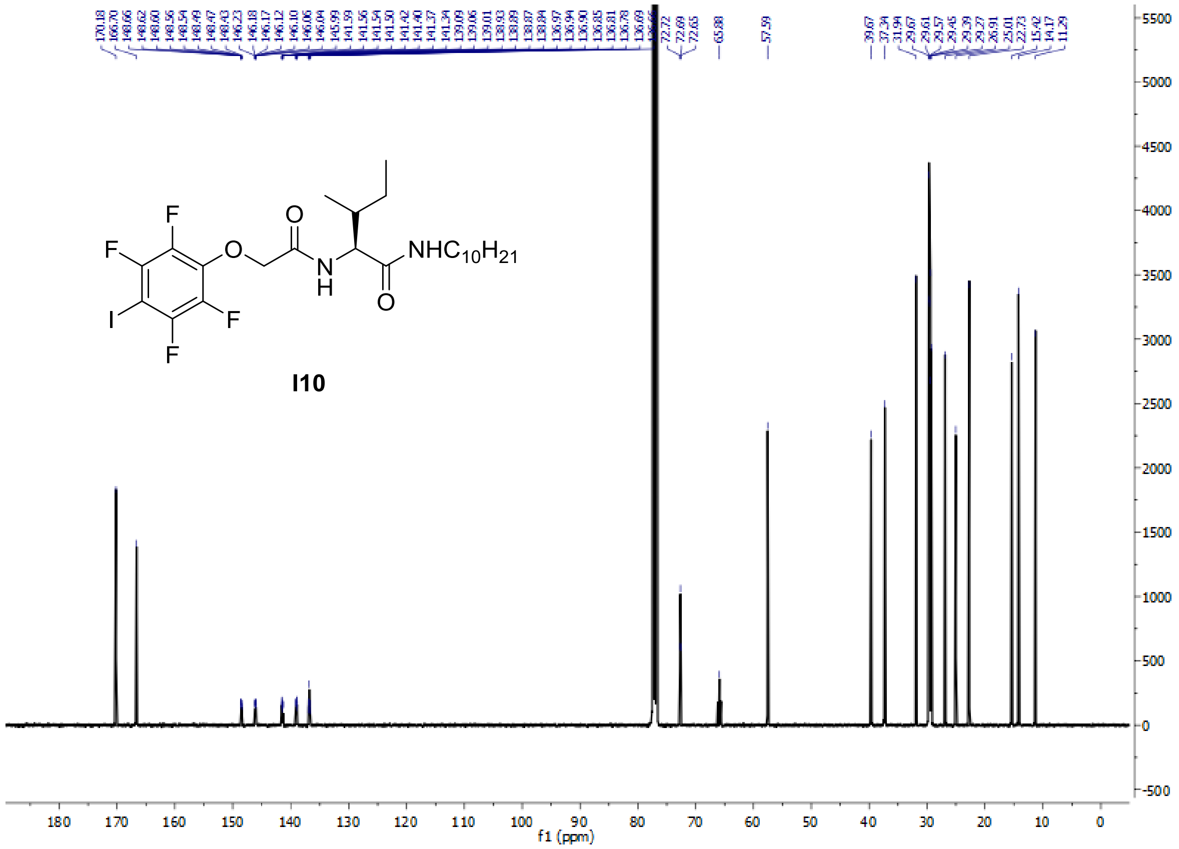
F
F
I
F
F
O
O
N
H
NHC10H21
O
I10
f1 (ppm)

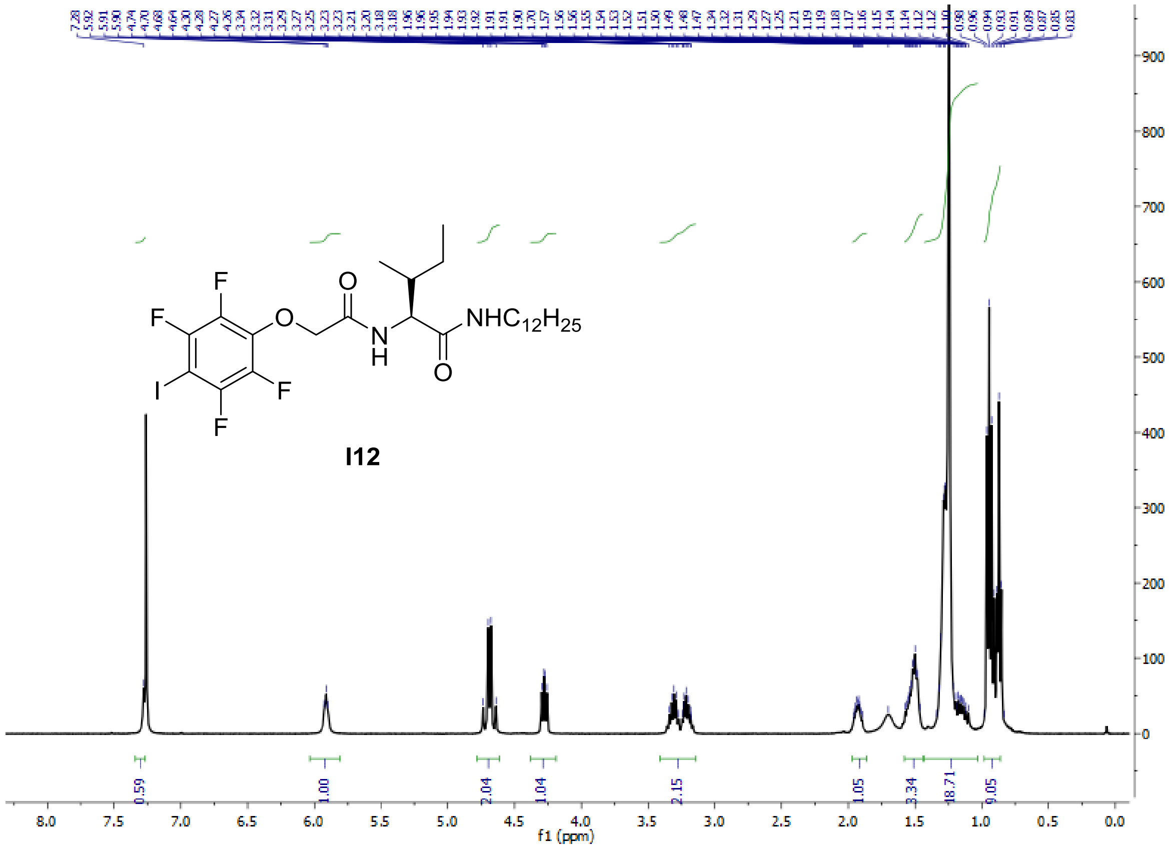
F
F
O
O
N
H
NHC12H25
O
I
F
F
I12
0.59
1.00
2.04
1.04
2.15
1.05
3.34
18.71
9.05
f1 (ppm)

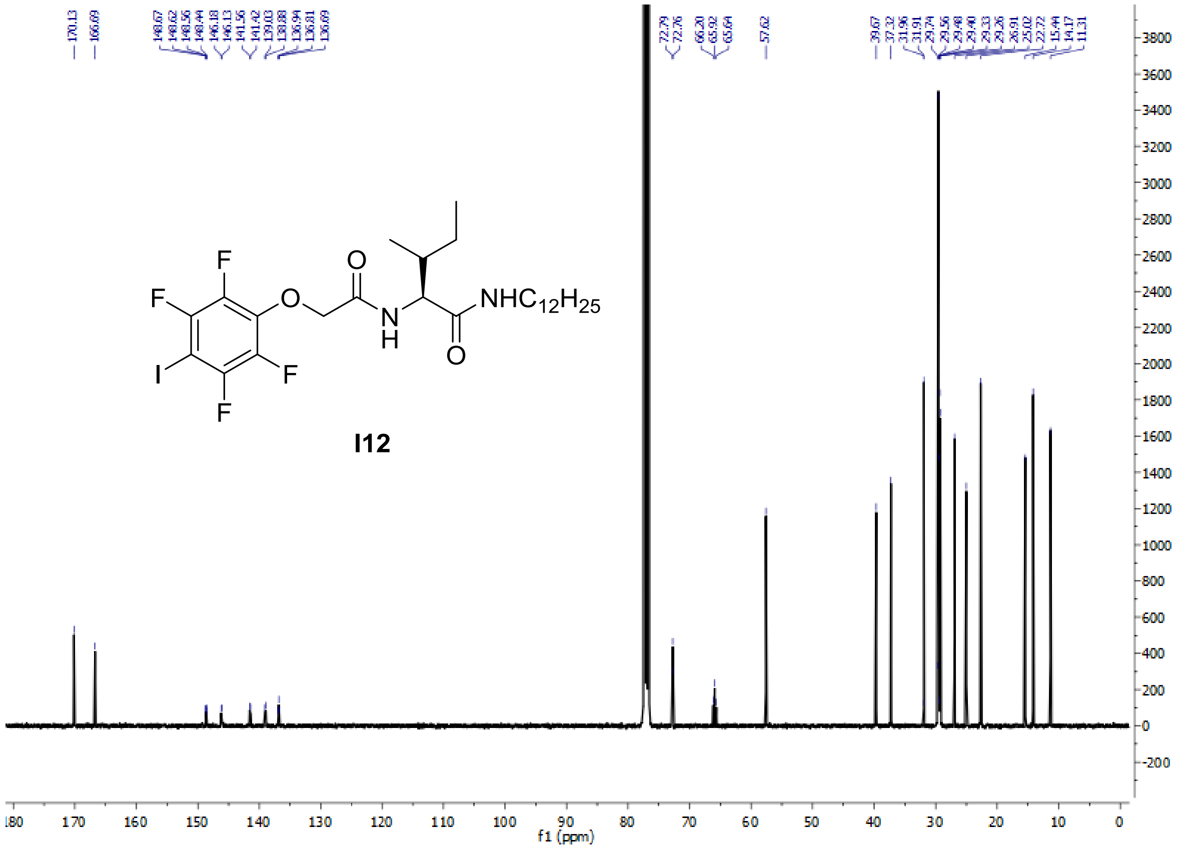
170.13
166.69
72.79
72.76
57.62
F
F
O
O
N
H
NHC12H25
O
I
F
F
I12
f1 (ppm)

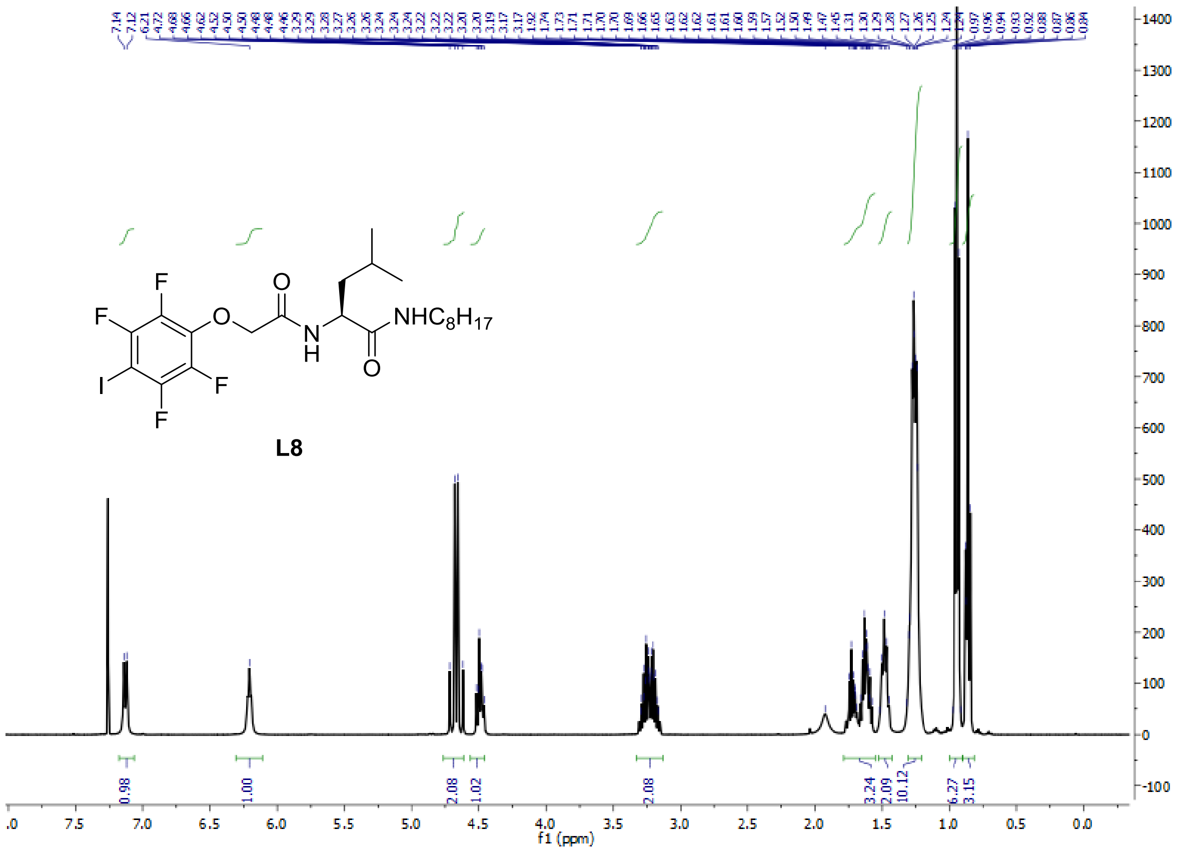
L8
NHC8H17
f1 (ppm)

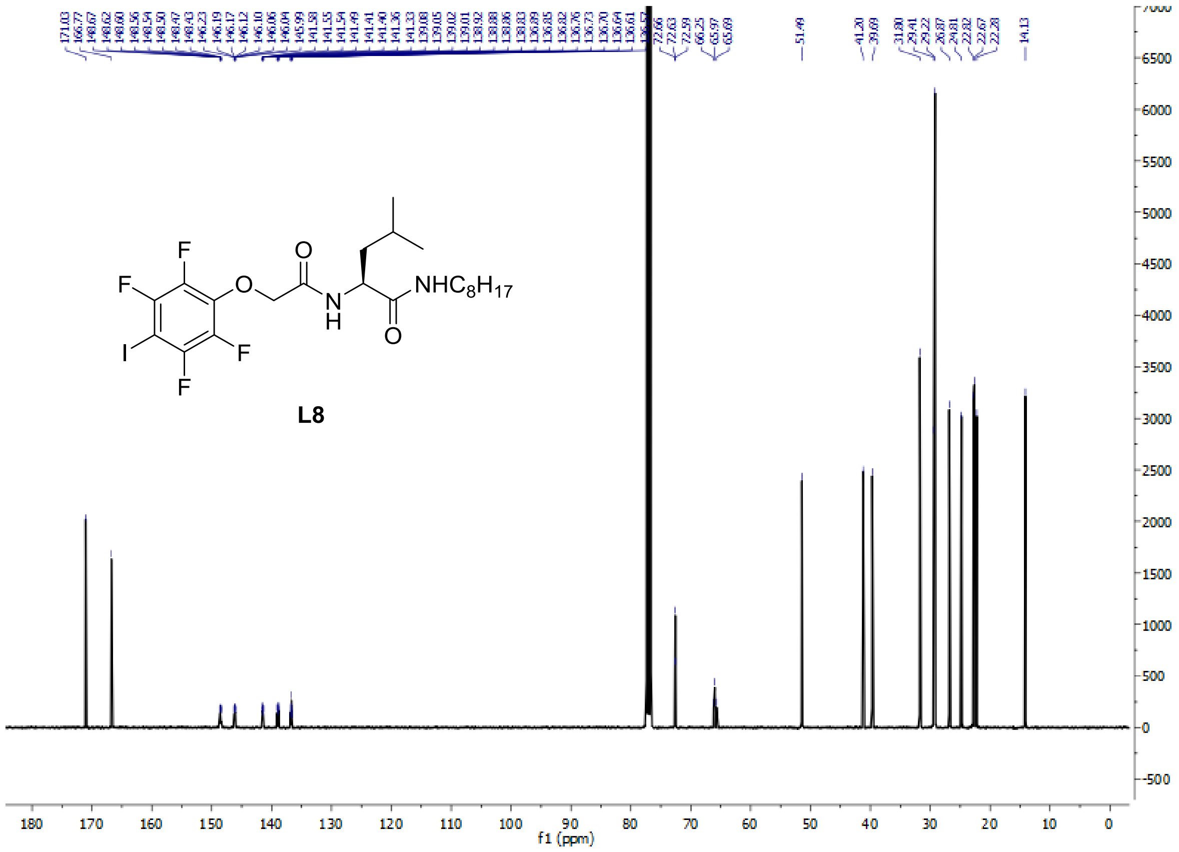
L8
NHC8H17
f1 (ppm)

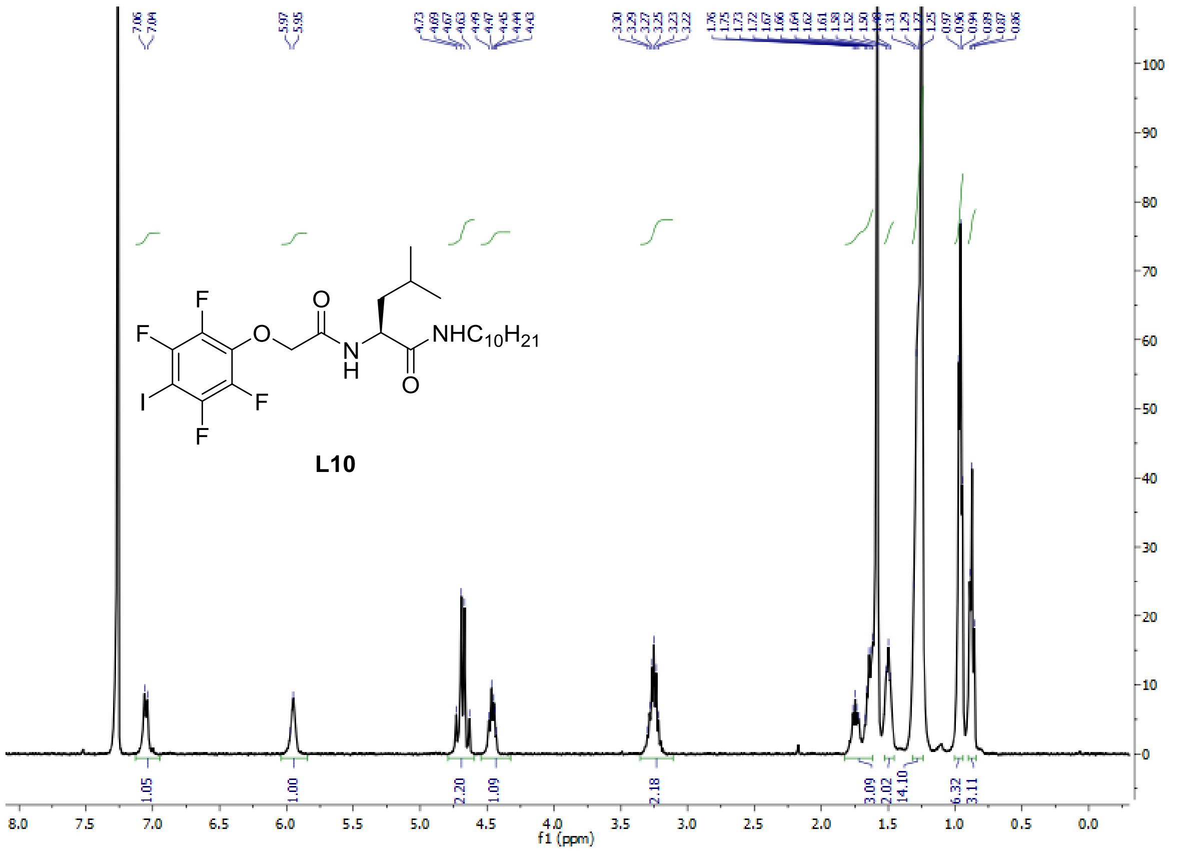
L10
f1 (ppm)

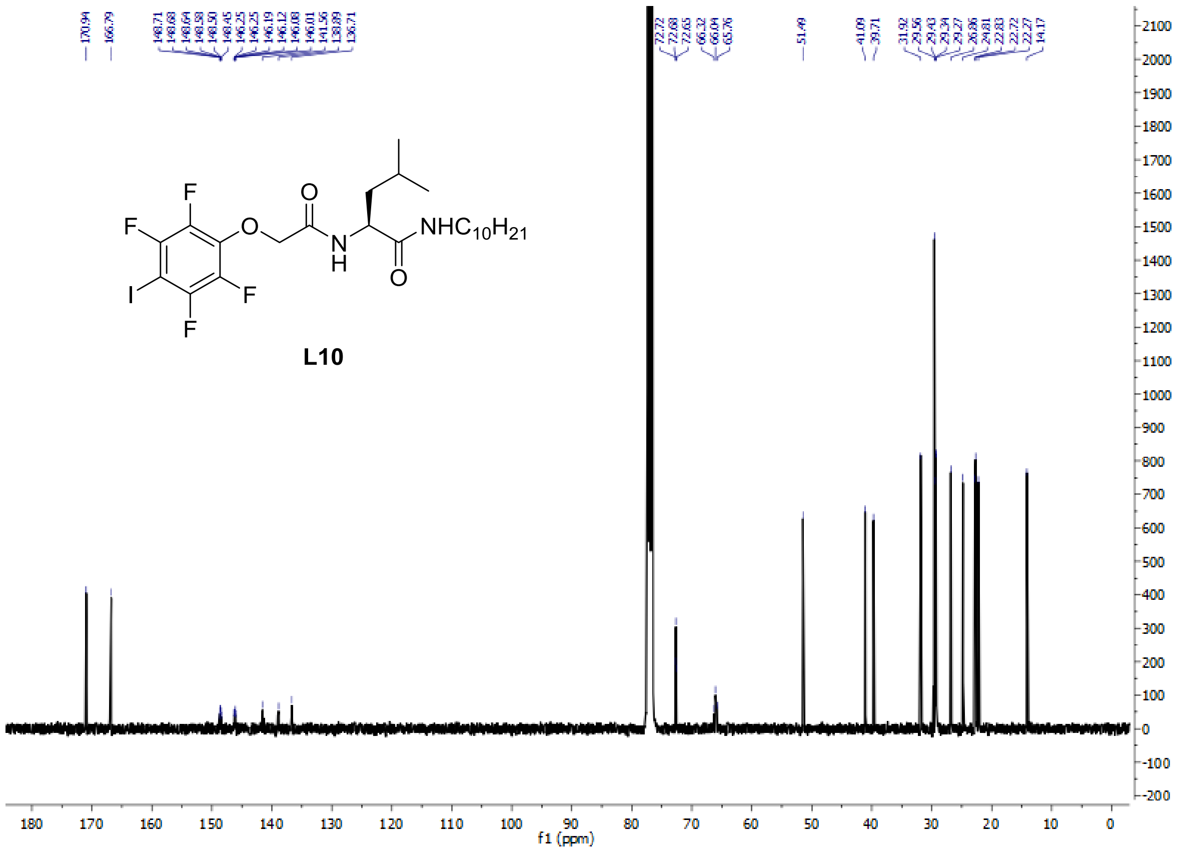
L10
f1 (ppm)

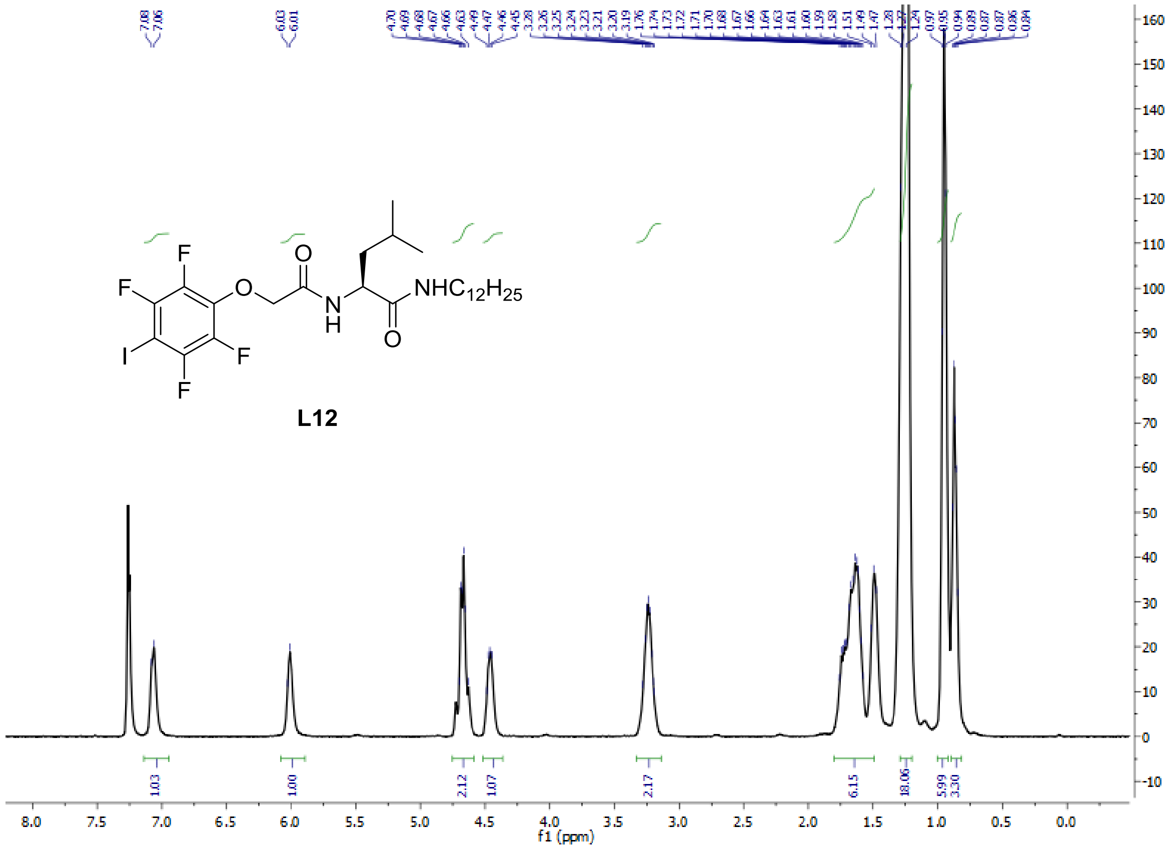

F
F
O
O
N
H
NHC12H25
O
I
F
F
L12
f1 (ppm)


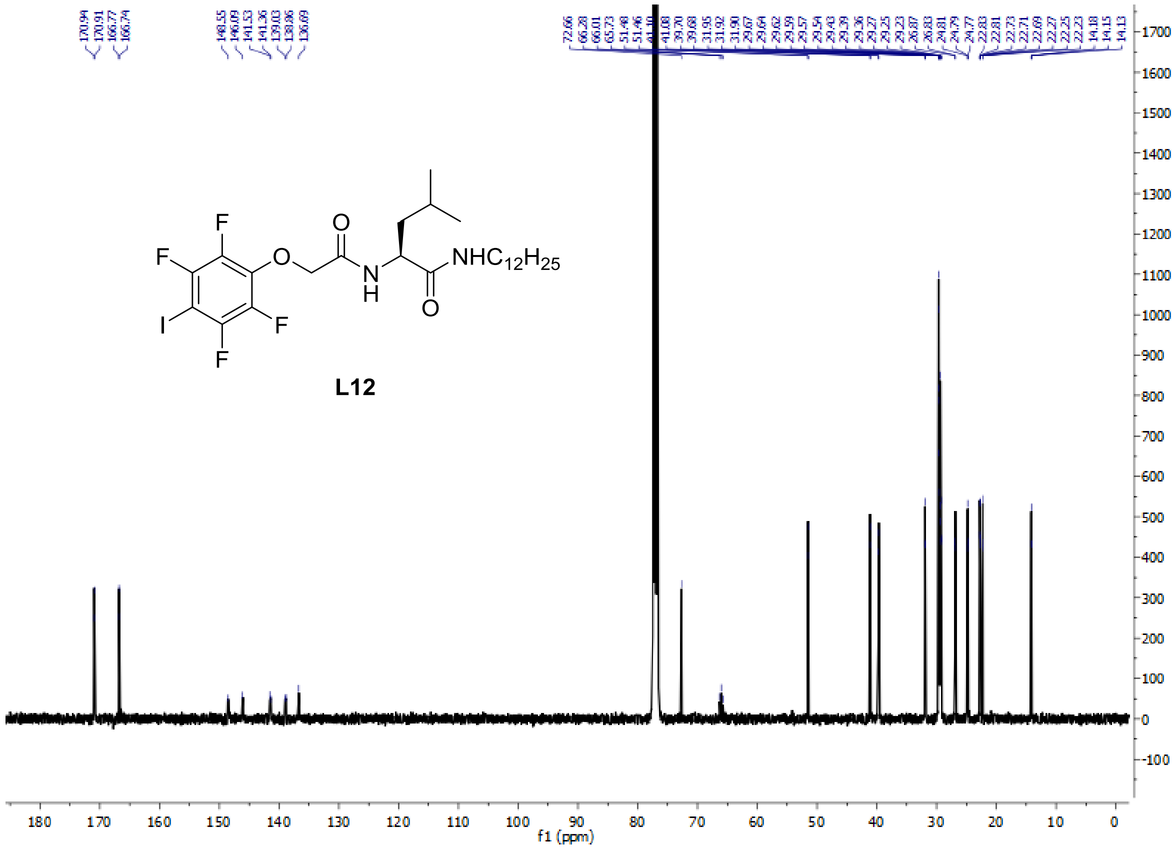

F
F
O
O
N
H
NHC12H25
O
I
F
F
L12
f1 (ppm)

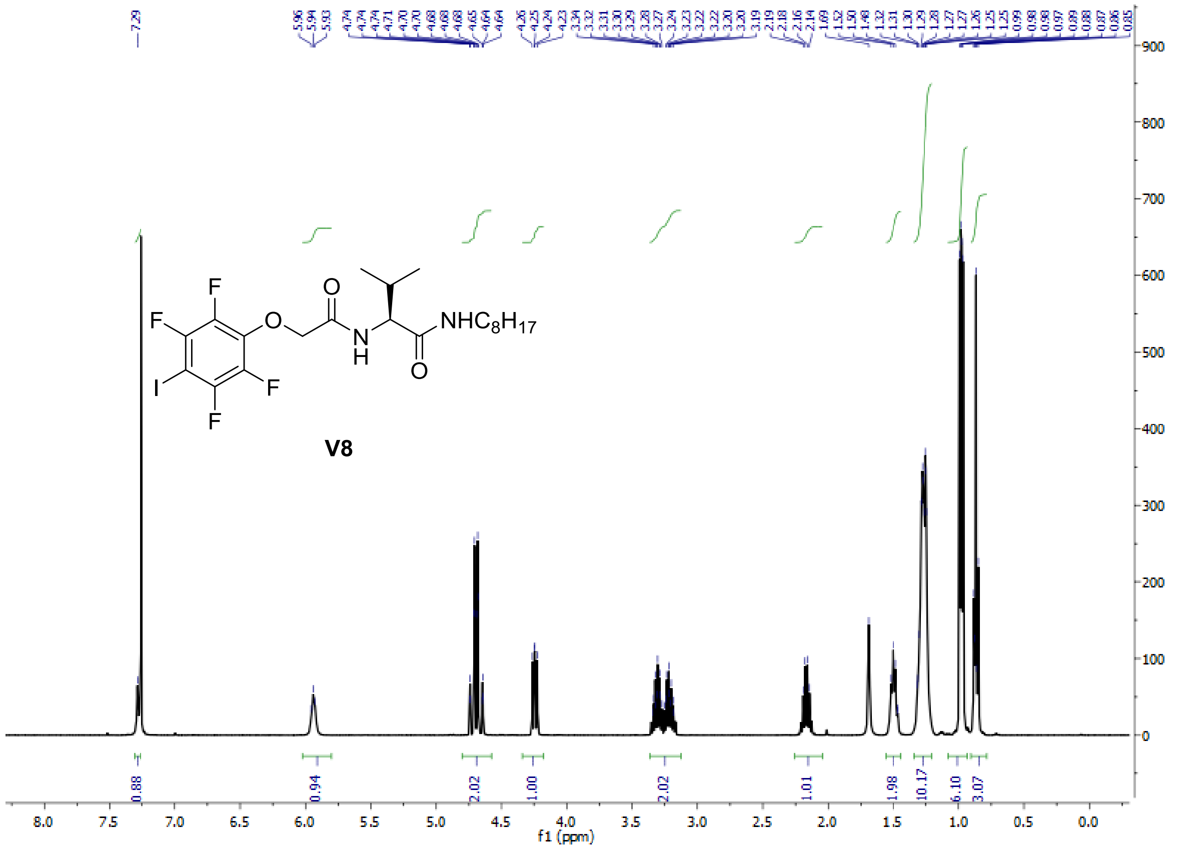

V8
f1 (ppm)


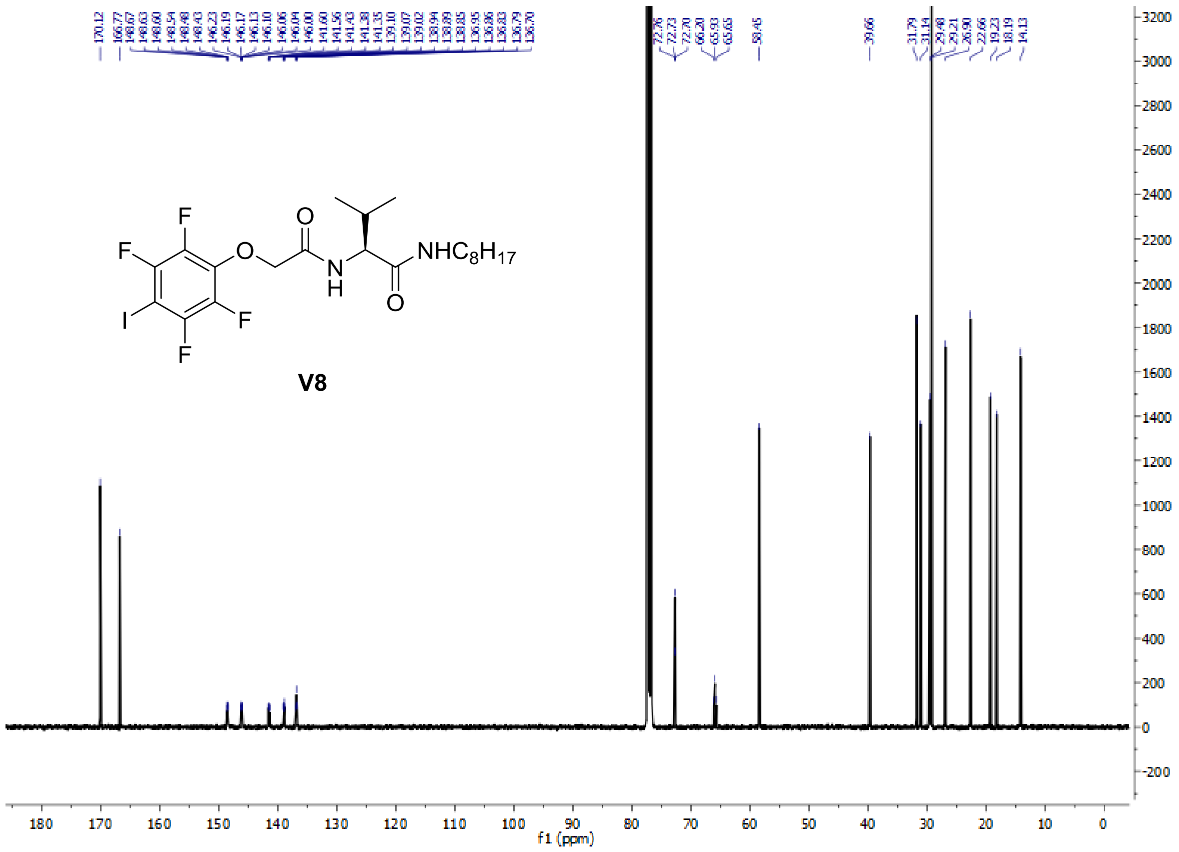

V8
f1 (ppm)

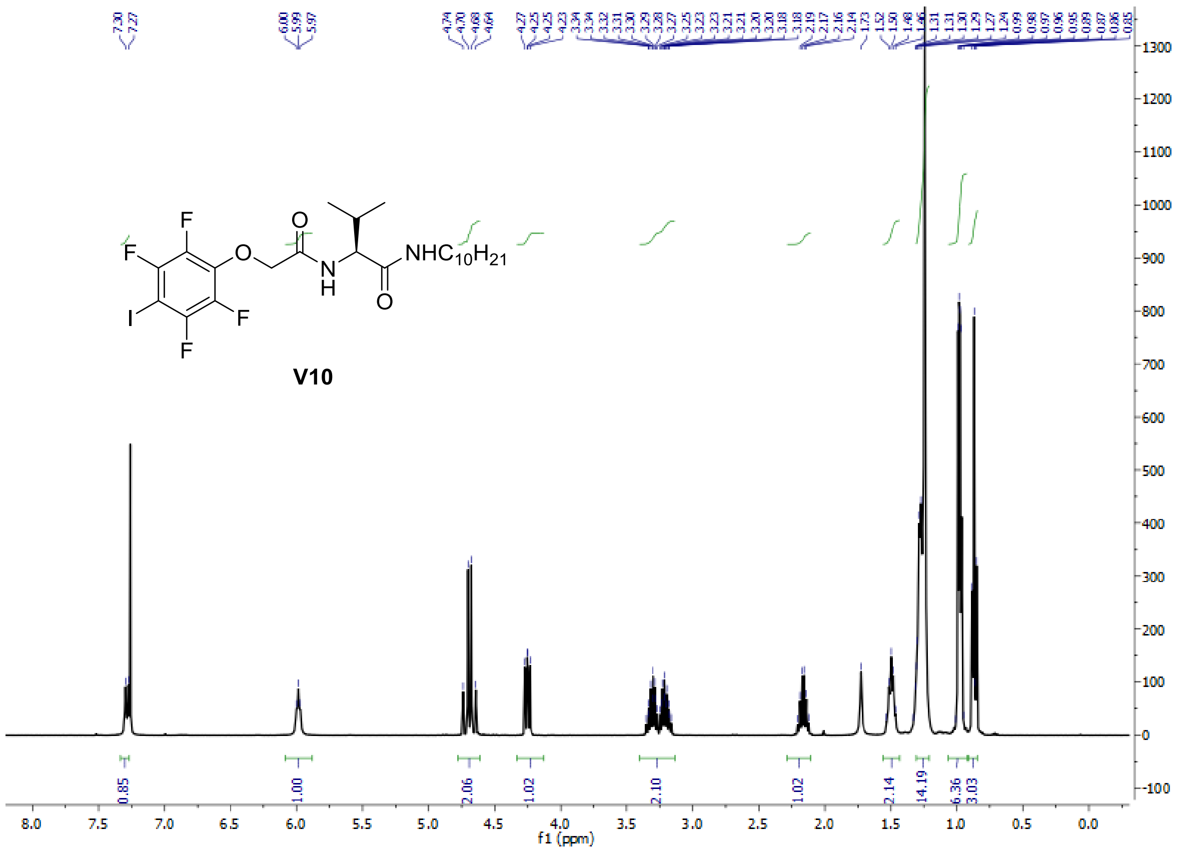

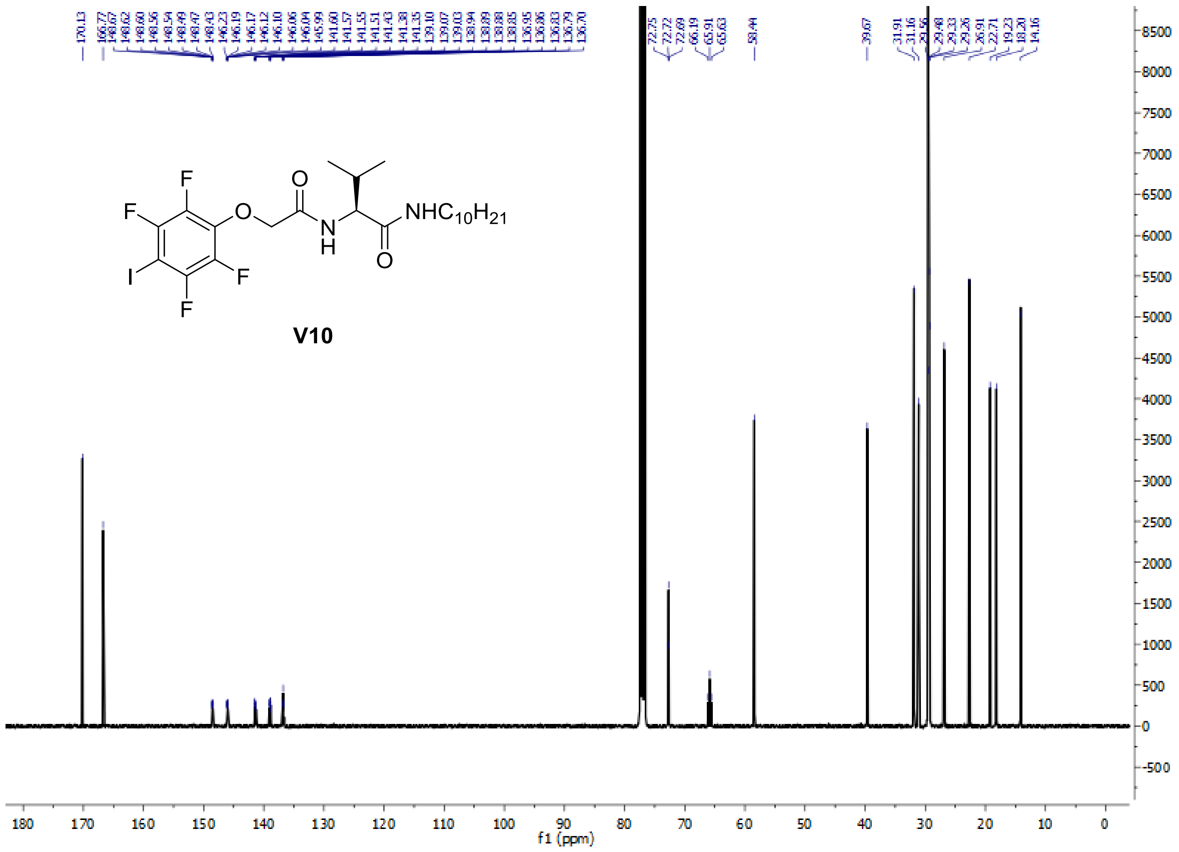

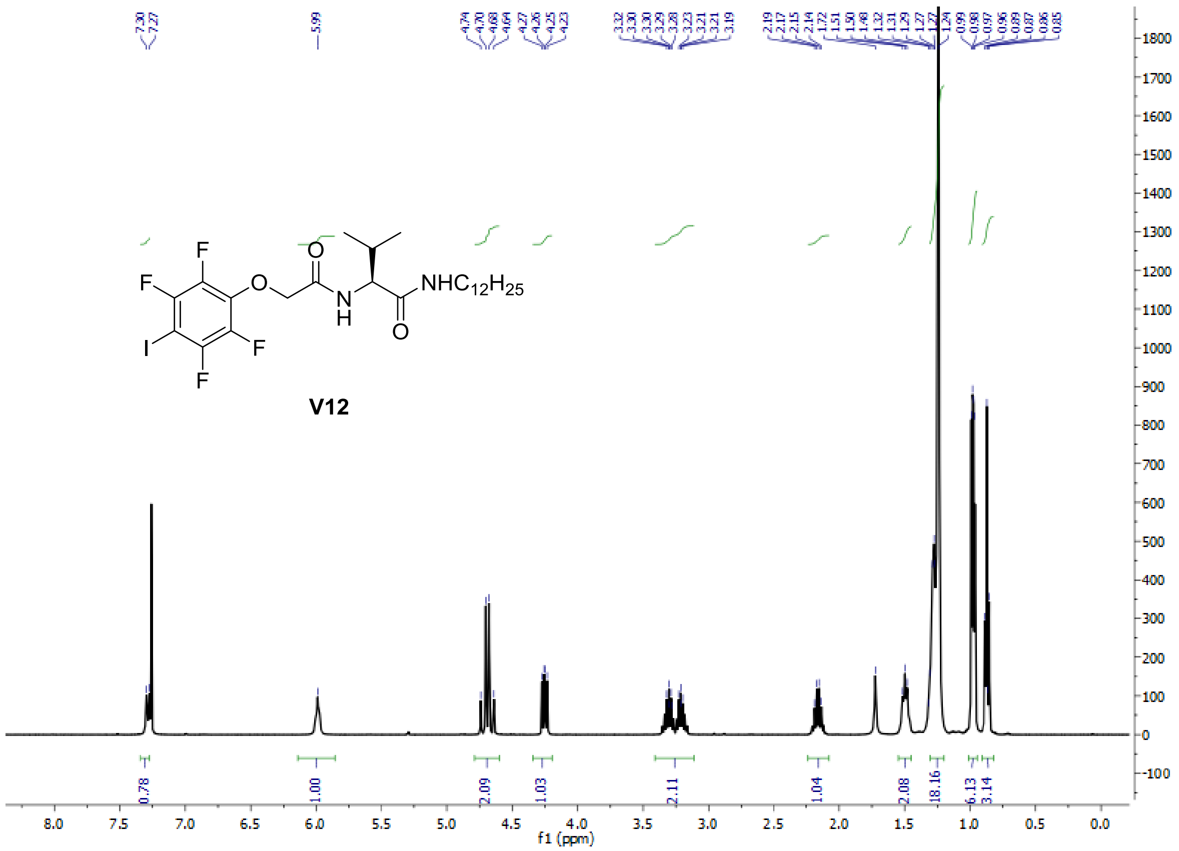

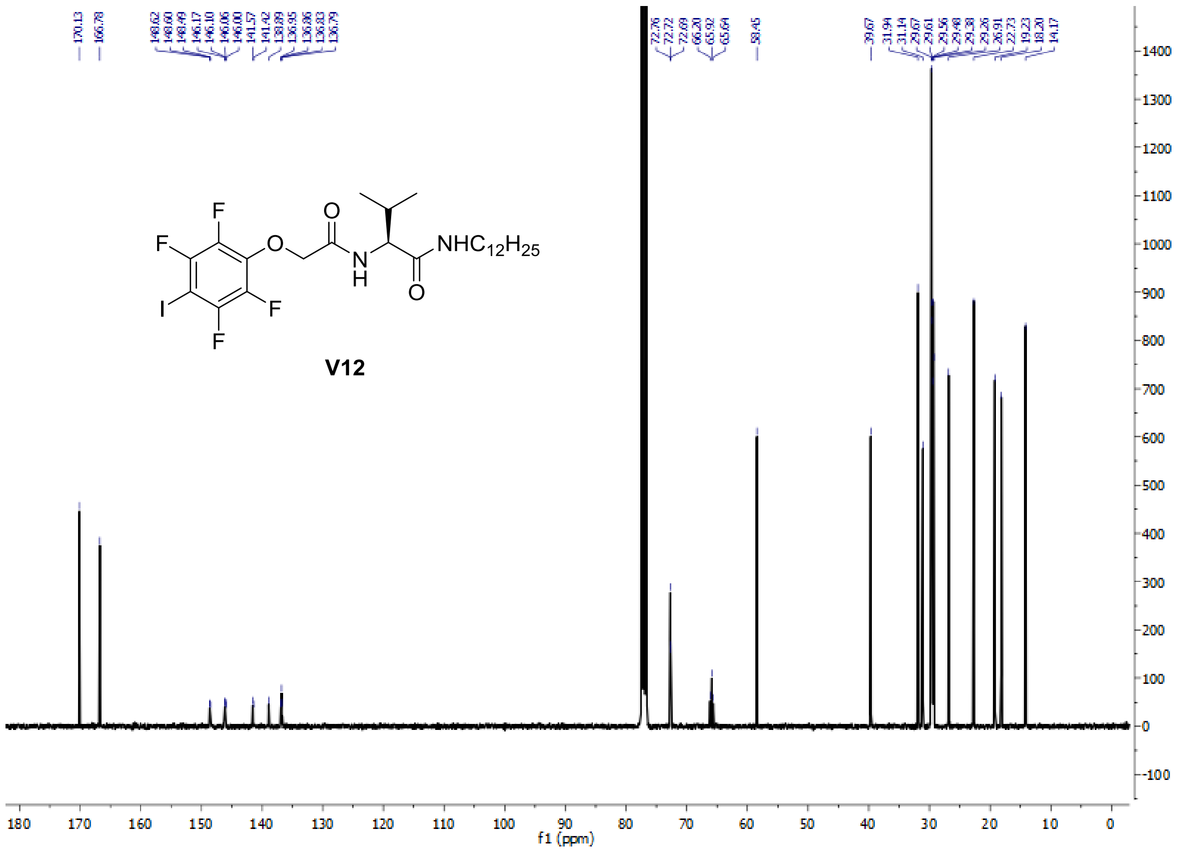

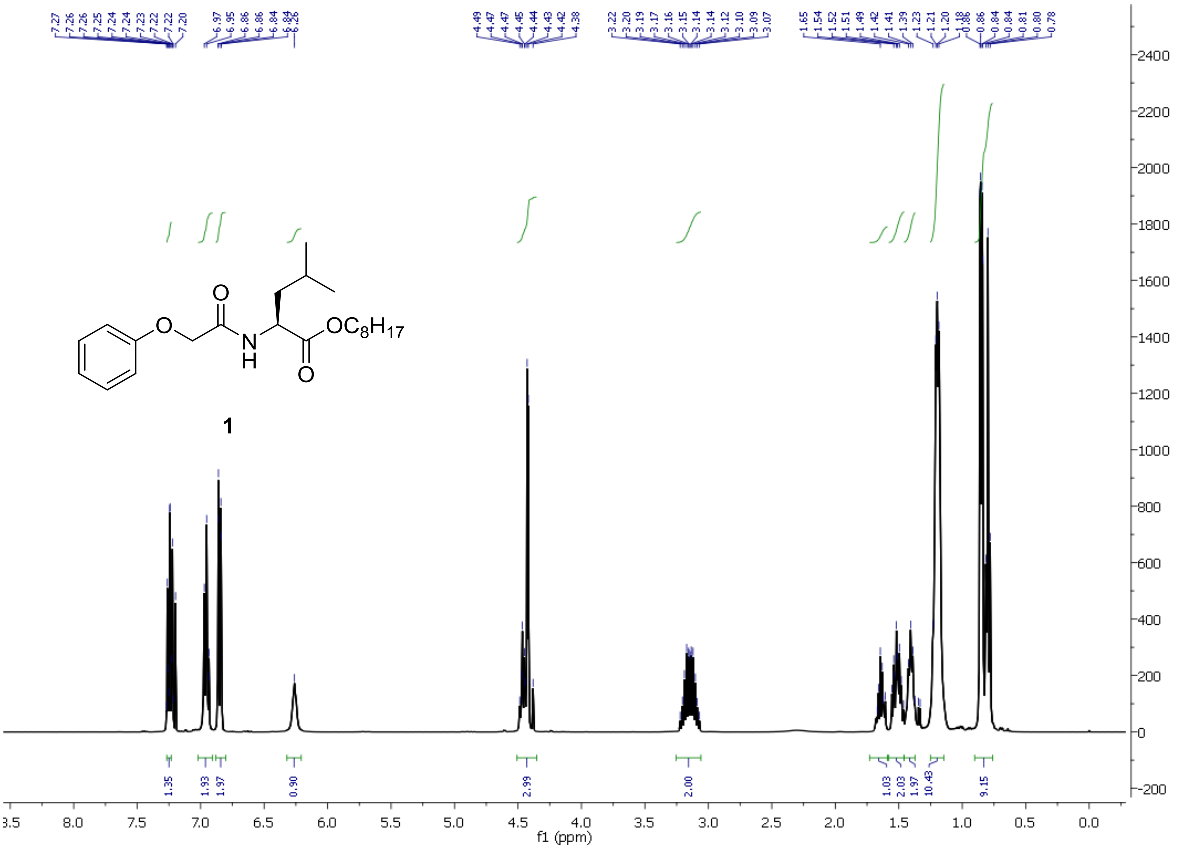

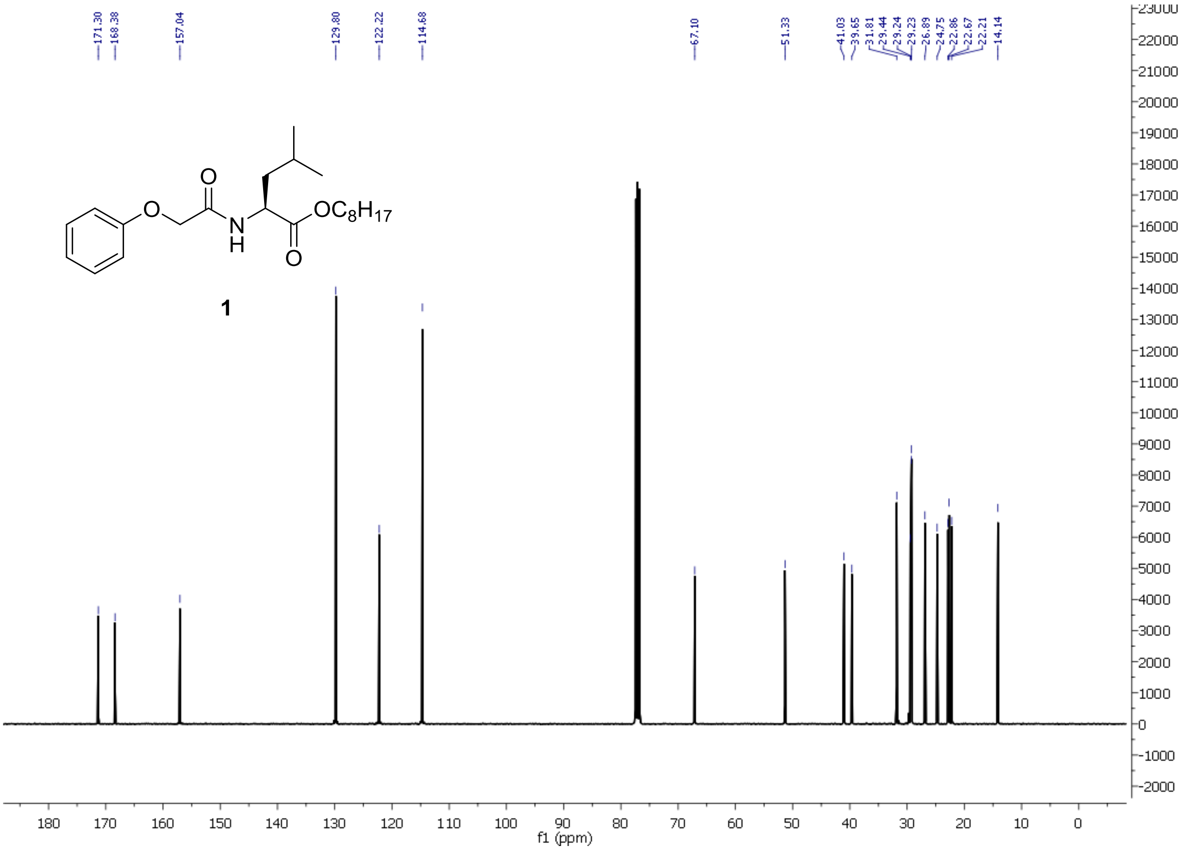

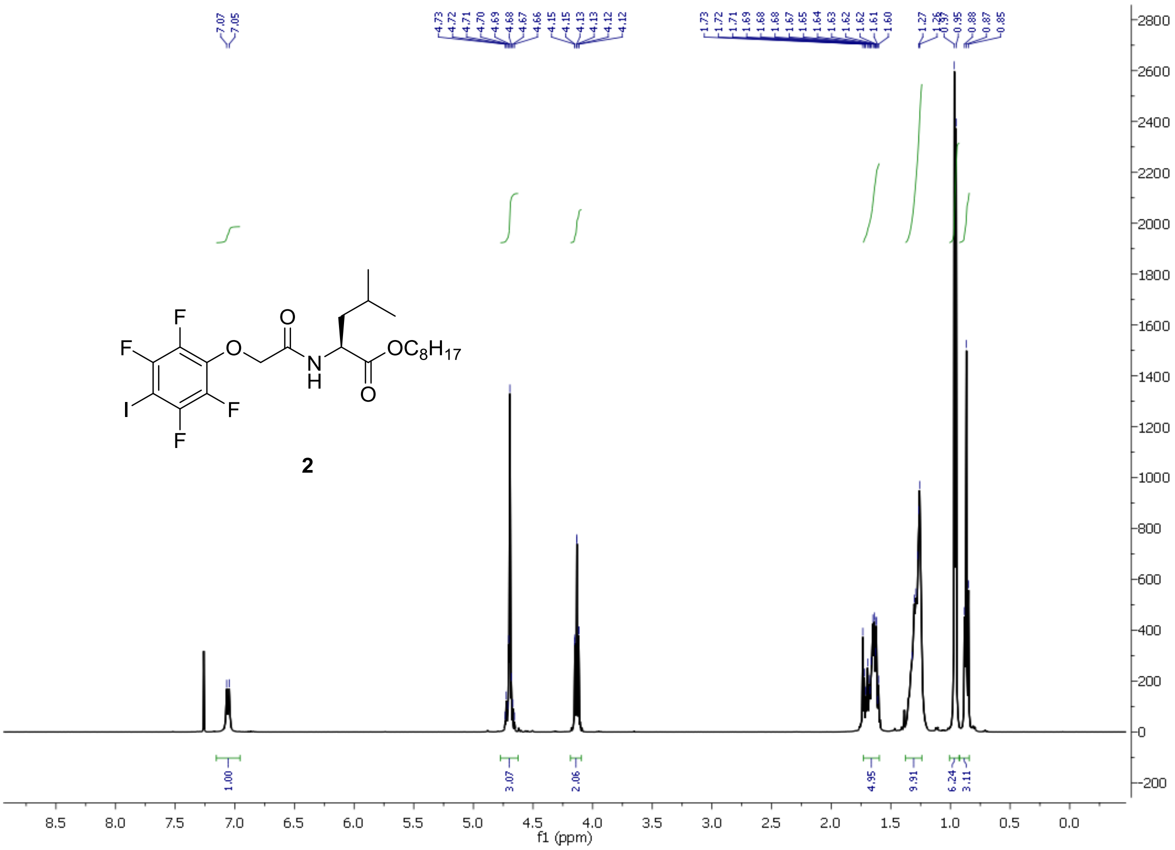

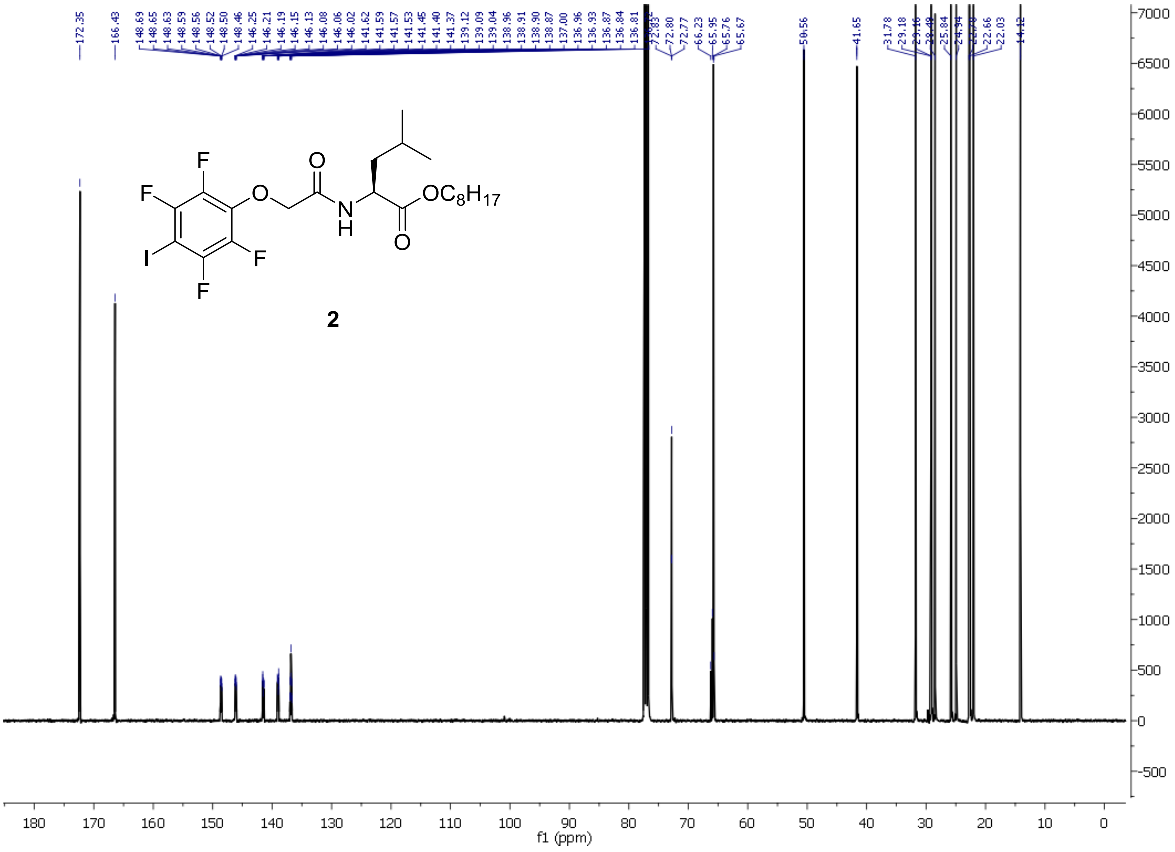

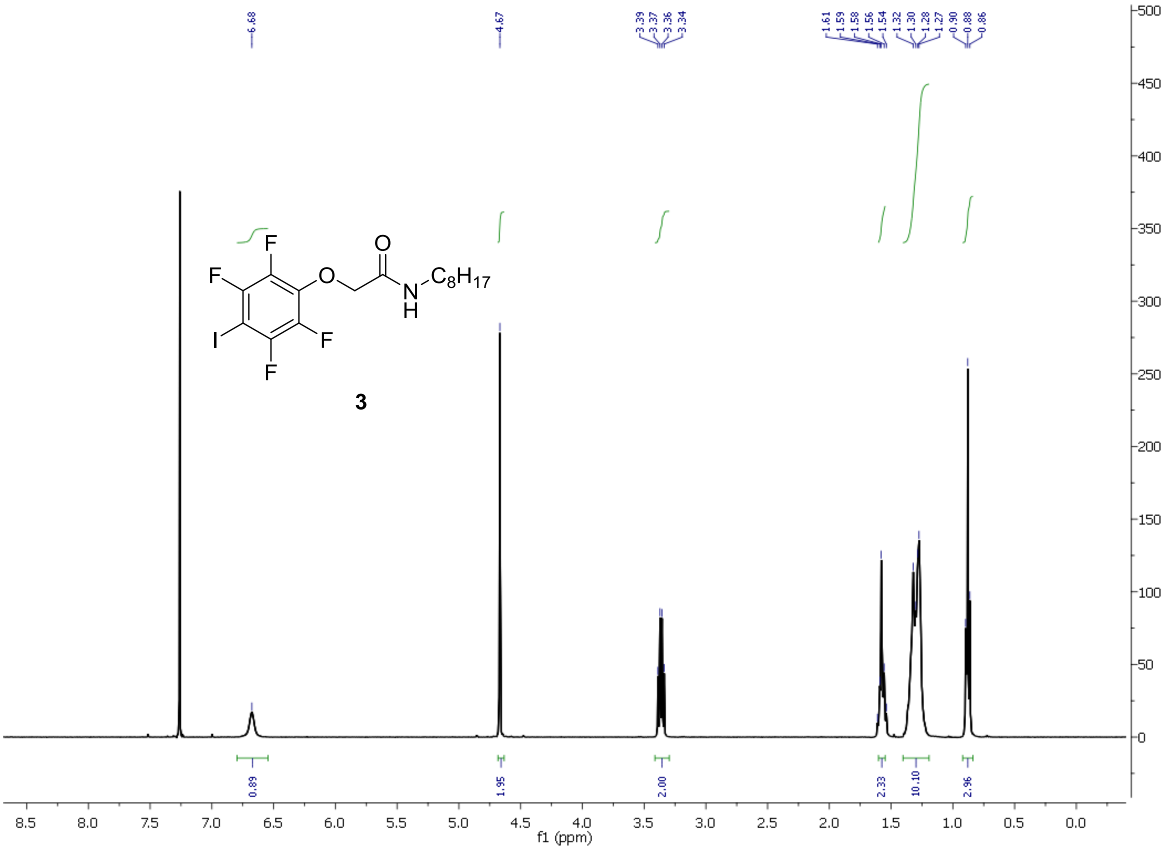

6.68
4.67
3.39
3.37
3.36
3.34
1.61
1.59
1.58
1.56
1.54
1.32
1.30
1.28
1.27
0.90
0.88
0.86
0.89
1.95
2.00
2.33
10.10
2.96
3
f1 (ppm)


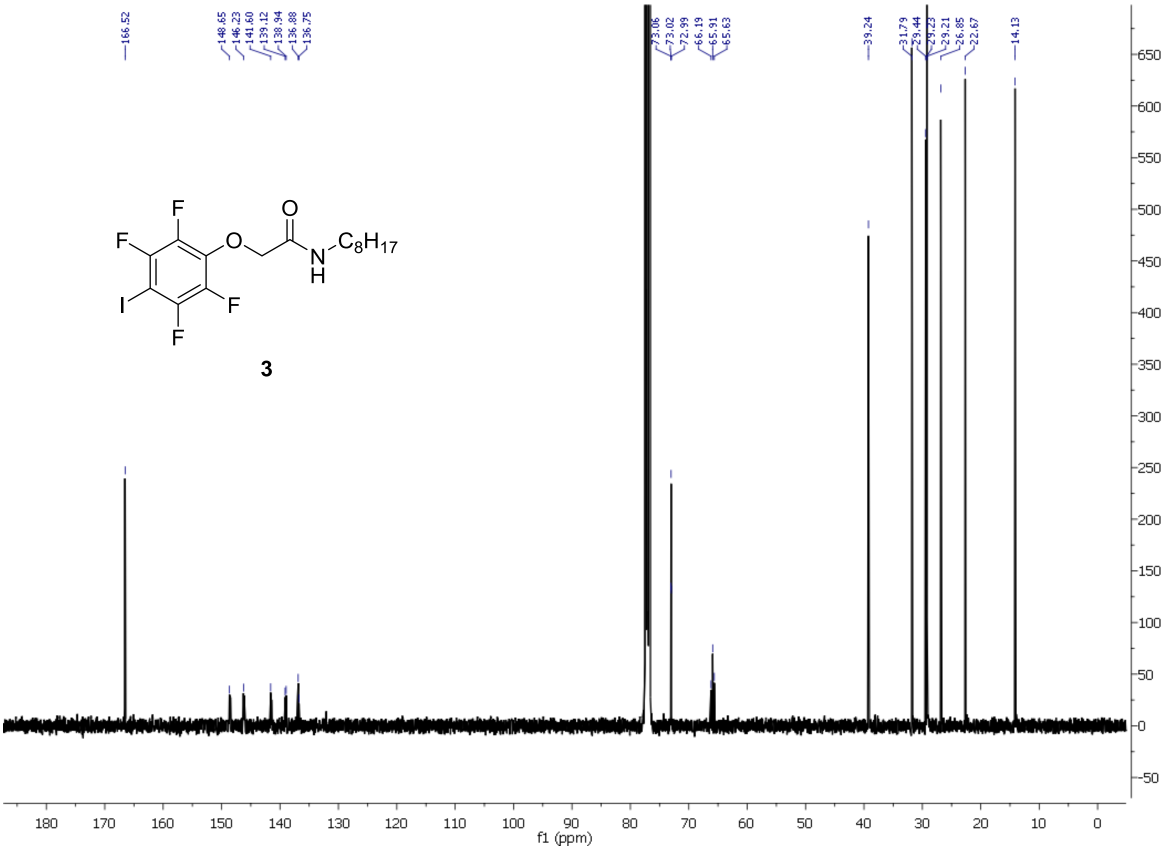

166.52
148.65
146.23
141.60
139.12
138.94
136.88
136.75
73.06
73.02
72.99
66.19
65.91
65.63
39.24
31.79
29.44
29.23
29.21
26.85
22.67
14.13
3
f1 (ppm)

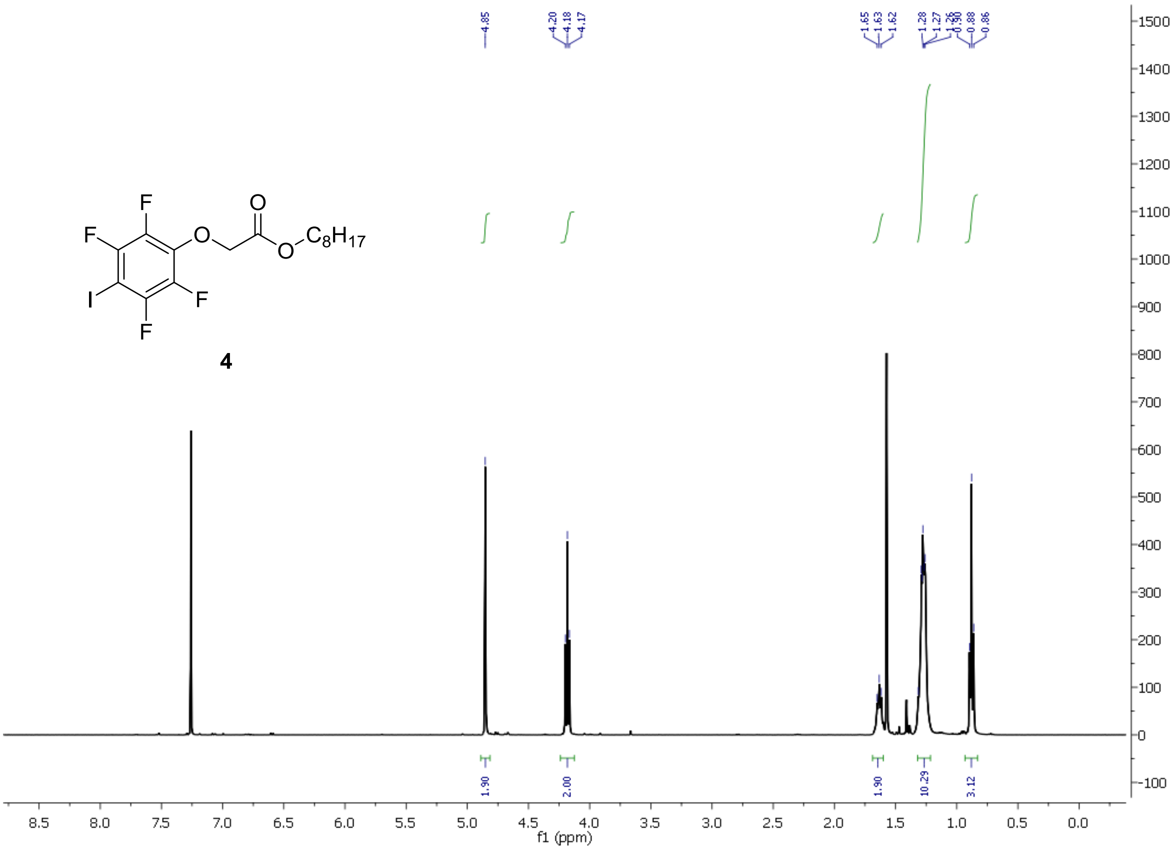
4.85
4.20
4.18
4.17
1.65
1.63
1.62
1.28
1.27
0.88
0.86
1.90
2.00
1.90
10.29
3.12
4
f1 (ppm)

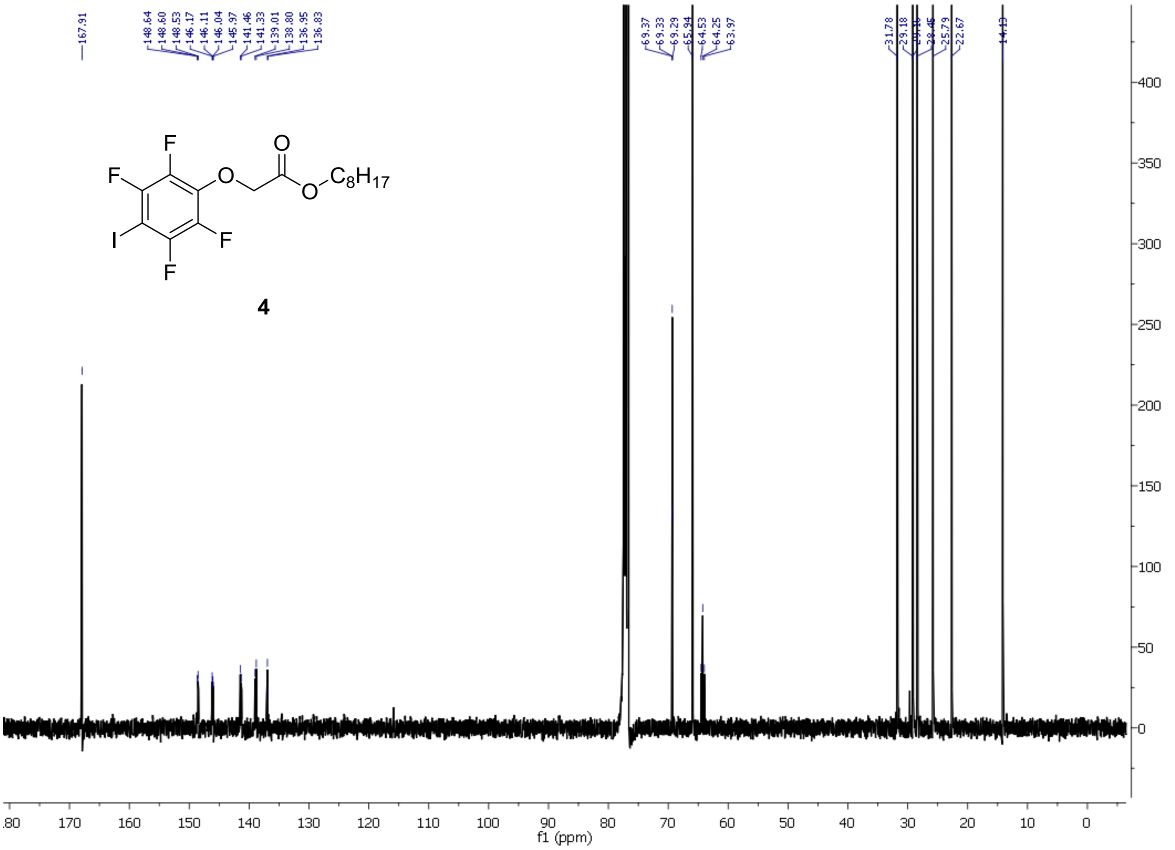
167.91
148.64
148.60
148.53
146.17
146.11
146.04
145.97
141.46
141.33
139.01
138.80
136.95
136.83
69.37
69.33
69.29
64.53
64.25
63.97
31.78
29.18
25.79
22.67
14.13
4
f1 (ppm)
